# The interaction of Multiple Sclerosis risk loci with Epstein-Barr virus phenotypes implicates the virus in pathogenesis

**DOI:** 10.1038/s41598-019-55850-z

**Published:** 2020-01-13

**Authors:** Ali Afrasiabi, Grant P. Parnell, Sanjay Swaminathan, Graeme J. Stewart, David R. Booth

**Affiliations:** Centre for Immunology and Allergy Research, Westmead Institute for Medical Research, University of Sydney, Sydney, Australia

**Keywords:** Gene expression, Genome-wide association studies, Autoimmunity, Genetics research, Risk factors

## Abstract

Translating the findings of genome wide association studies (GWAS) to new therapies requires identification of the relevant immunological contexts to interrogate for genetic effects. In one of the largest GWAS, more than 200 risk loci have been identified for Multiple Sclerosis (MS) susceptibility. Infection with Epstein-Barr virus (EBV) appears to be necessary for the development of Multiple Sclerosis (MS). Many MS risk loci are associated with altered gene expression in EBV infected B cells (LCLs). We have interrogated this immunological context to identify interaction between MS risk loci and EBV DNA copy number, intrinsic growth rate and EBV encoded miRNA expression. The EBV DNA copy number was associated with significantly more risk alleles for MS than for other diseases or traits. EBV miRNAs BART4-3p and BART3-5p were highly associated with EBV DNA copy number and MS risk loci. The poliovirus receptor (PVR) risk SNP was associated with EBV DNA copy number, PVR and miRNA expression. Targeting EBV miRNAs BART4-3p and BART3-5p, and the gene PVR, may provide therapeutic benefit in MS. This study also indicates how immunological context and risk loci interactions can be exploited to validate and develop novel therapeutic approaches.

## Introduction

Translation of genome wide association findings to clinical outcomes has been slow, especially due to the problems of linking association to causality, the pleiotropic nature of most genes, and identification of the relevant immunological contexts to interrogate for genetic effects. Genetic effects are nearly always on expression and exon splicing, since the most associated risk SNPs are rarely located in exons^1^. Interrogation of expression has indicated risk allele effects are highly dependent on immunological context. Even though risk genes may be predominantly expressed in immune cells, as in the risk genes for autoimmune diseases, or expressed in monocytes rather than lymphocytes, the risk allele in monocytes cultured with one stimulant may have opposite effects on expression compared to with another stimulant^2,3^. If the effect on expression that leads to pathogenic effect is to be determined the context in which this occurs *in vivo* needs to be determined.

Multiple Sclerosis genome wide associations have been identified from comparison of more than 100,000 individuals genotyped for more than a million alleles^4^. More than 200 independent statistically robust associations have been identified. These are mostly from genes predominantly expressed in particular immune cells^5^. Of these immune cells, B cells, T cells, NK cells and monocyte and monocyte derived cells are implicated in MS susceptibility from the risk gene expression. This is consistent with the recent generation of successful therapies, which target these cells generally^6^. Particularly notable are B cells, which are targeted specifically by antibodies specific for naive and memory B cells^7^. These therapies are useful for relapsing and progressive forms of MS, indicating the importance of B cells in both types of disease^8^.

The therapeutic benefit of reducing B cells may be due reducing the load of Epstein-Barr virus^9^. This virus infects B cells specifically, and has been demonstrated as necessary but not sufficient for development of MS^10^. A number of models of the mechanism for this have been proposed^11^. These require that EBV infected B cells evade immune control to produce immortalised clones targeting myelin antigens, and/or promoting T cell responses to myelin. The immune evasion itself may represent poor identification and killing of infected B cells, predominantly by Natural Killer and CD8 T cells^12^.

B cells infected with EBV at latency III result in lymphoblastoid cell lines (LCLs). We recently published evidence that response to EBV latency III infection contributed to MS susceptibility^13^. The number of MS risk SNPs associated with genes differentially expressed between infected and uninfected B cells; and the number of MS risk SNPs where the genotype was associated with proximal gene expression (called LCLeQTLs) are significantly more than would be expected by chance. Extending from the findings of Harley *et al*. (2017)^14^, we found that EBV transcription factor binding sites are highly over-represented among MS risk genes and among LCLeQTLs. The LCLeQTLs were also over-represented on the major LCL Infected B cell proliferation pathway. This pathway is controlled through signalling mediated by the EBV protein LMP1. The MS risk gene CD40 is a homologue of LMP1, and the risk gene TRAF3 binds to both CD40 and LMP1. The protective genotypes of CD40 and TRAF3 inhibit LCL proliferation on CD40L binding. These data indicate many genetic risk factors identified in genome wide association studies for MS susceptibility have roles consistent with a dysregulated response to EBV infection, and so in this way contribute to MS pathogenesis.

A caveat is that MS risk SNP LCL effects may be due to B cell processes contributing to disease, and independent of the role in EBV latency III. For example, the interaction between CD40/TRAF3/LMP1 would predict protective genotypes decrease EBV latency III proliferation, this proliferation may be independent of the pathogenic effect in MS of these genes. Further, although some 25% of MS risk loci were LCLeQTLs, we present evidence here that a similar percentage of risk SNPs for non-EBV associated traits are LCLeQTLs. Here we sought an EBV phenotype that might more specifically indicate if the risk SNP interactions with EBV were indicating a role for EBV in disease, and so identify therapeutic targets.

Extensive data is available for EBV DNA copy number, LCL intrinsic growth rate, host genotype (>85 million SNPs) and EBV and host expression in over 400 LCLs^15–18^. For most of the models of EBV involvement in MS susceptibility a key feature is the immortalisation of EBV infected B cells. EBV DNA copy number has been shown to predict immortalisation^19^, be necessary for initiation of late transcription EBV genes and cell lysis and proliferation of infection^20^. A genome wide association analysis from 19 populations and LCLs from 1736 individuals indicated host genetic variation was associated with EBV DNA copy number^18^.

In this study we aimed to determine if host genetic variation could alter the EBV phenotypes and increase EBV pathogenesis in MS. To do this we established MS risk loci interaction with EBV phenotypes by determination of the correlation of EBV DNA copy number, intrinsic growth rate and gene expression with MS risk gene expression, and the association with MS risk genotypes.

Collectively, these data support the hypothesis that EBV DNA copy number is correlated with expression of MS risk genes, associated with risk variants, and affected by these in a manner suggesting that targeting EBV DNA copy number would reduce EBV pathogenesis in MS. By reducing the interaction between the most associated host MS risk genes and EBV genes affecting DNA copy number this pathogenic process could be inhibited in MS.

## Results

### MS risk SNPs are not over-represented in LCLeQTLs compared to risk SNPs from other diseases

**Figure 1:**
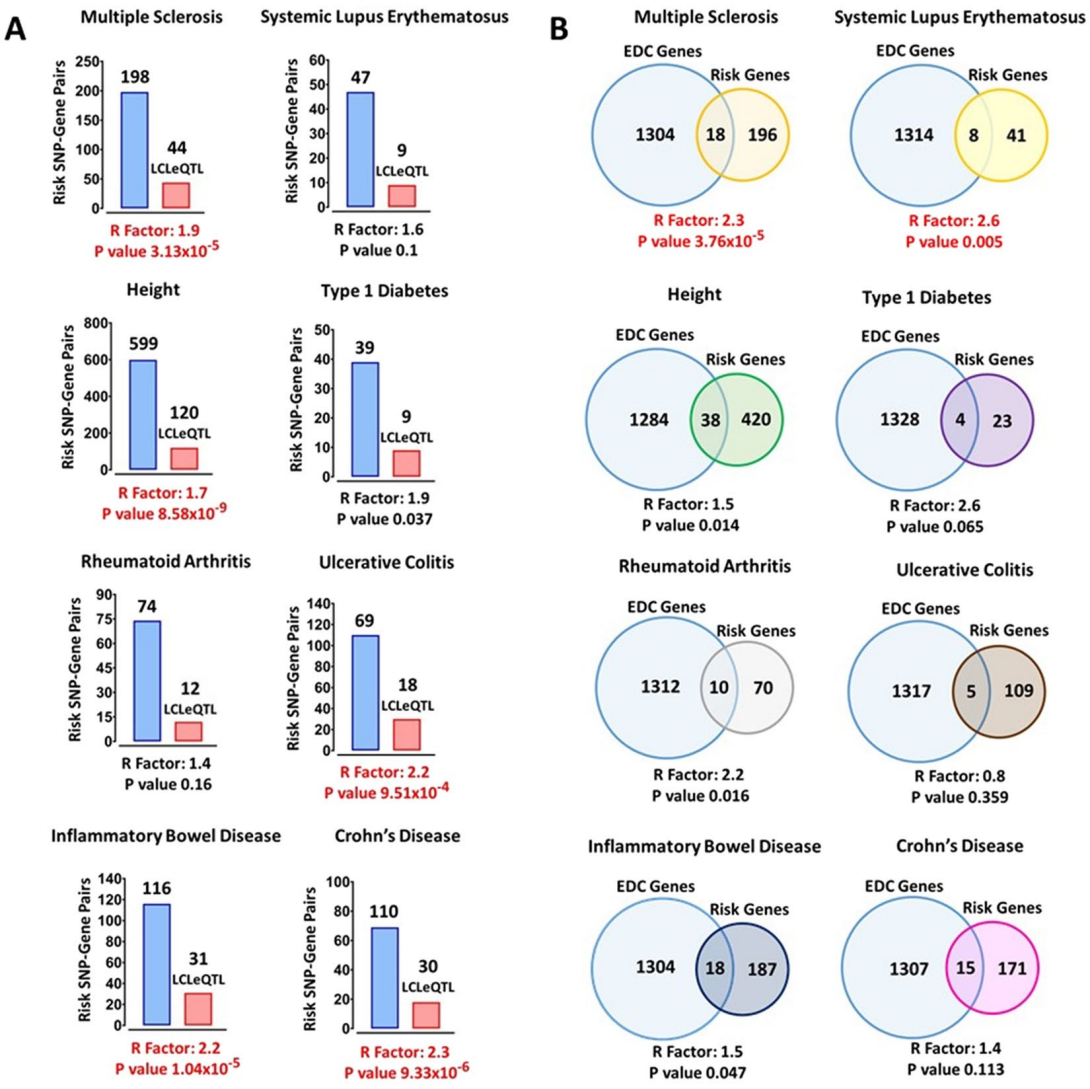
(**A**) Over-representation of disease/trait risk SNP-gene pairs that in eQTL in LCL (LCLeQTL) among all significant LCLeQTLs (FDR < 5%). (**B**) Over-representation of disease/trait risk loci (genes) whose expression was correlated with EBV DNA copy number in LCLs (FDR 5%). The Representation (R) Factor indicates the ratio of overlapping genes relative to expected number of overlapping genes by chance: >1 means more than by chance, <than 1 means less than by chance. The p value is based on the exact hypergeometric probability test. LCLeQTL: The SNP-Gene pair which is in eQTL in LCLs^13^. The red bar indicates the subset of all SNP-Gene pairs, blue bar, which are in eQTL in LCLs. The disease risk genes were identified in related GWAS (see supplementary Table 1 for references).

Earlier we had identified MS risk SNPs associated with expression of their proximal genes in LCLs (LCLeQTLs)^13^. Some 25% of MS risk SNPs were LCLeQTLs. To test if this was more common than for other autoimmune diseases, we investigated the risk SNPs for the EBV associated disease systemic lupus erethymatosus – SLE, other autoimmune diseases where EBV association is not established, Crohn’s Disease – CD; Ulcerative Colitis – UC; both as Inflammatory Bowel Disease – IBD; Rheumatoid Arthritis – RA, and Type 1 Diabetes – T1D. We also interrogated the risk SNPs for height, based on a statistically powerful GWAS has been completed for it^21^. We determined the LCLeQTLs for each of these conditions (Supplementary Table 1). At a overrepresentation p < 0.01, for MS, IBD, UC, CD and height there was an excess of condition risk loci among LCLeQTLs, but not for SLE, RA, and T1D (Fig. 1A). This parameter on its own is not supporting an association of risk loci with EBV infection.

### EBV LCL Phenotypes

**Figure 2:**
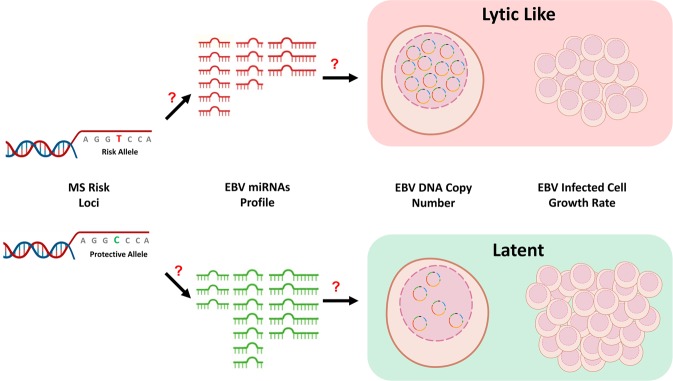
Schema of project to determine if MS risk genetic background alters EBV phenotypes (EBV miRNA expression, DNA copy number and intrinsic growth rate) in lymphocytic cell lines. Figure created using the BioRender tool.

Risk SNPs could be associated with proximal gene expression in EBV infected B cells because they affect gene expression and so EBV proliferation, but also because they affect proliferation itself (eg through such processes as energy usage, cell differentiation, cell division, apoptosis), or activation of B cells specifically. To distinguish between an EBV link and the other causes of risk loci association with gene expression in LCLs we investigated risk gene interactions with EBV phenotypes by combining publicly available datasets of EBV miRNA and host gene expression in LCLs, host genotype for these LCLs, and EBV DNA copy number and intrinsic growth rate (IGR) for these LCLs (Fig. 2). EBV DNA copy number is inversely associated with LCL intrinsic growth rate (Supplementary Fig. 1). The former is stable for particular LCLs and GWAS for EBV DNA copy number indicate host genotype interactions^22^. We reasoned that MS risk loci could affect EBV DNA copy number and other EBV phenotypes to alter EBV pathogenic states, with loci for non EBV diseases and traits being less likely to do so. If MS risk loci were over-represented in genes/SNPs affecting these EBV phenotypes, compared to non-EBV diseases, it would support the role of at least some of these interactions in MS pathogenesis.

### MS risk genes are over-represented among host genes whose expression is correlated with EBV DNA copy number compared to risk genes for other diseases

We identified host gene expression correlated with EBV DNA copy number at a false discovery rate (FDR) of less than 5% (EDC genes) in 433 LCLs (Supplementary Table 2). Risk genes for MS were highly over-represented (p < 10^−5^), SLE genes also (p < 0.005), but the other conditions only slightly or not at all (Fig. 1B). If the association between MS risk loci and EBV DNA copy number was driving differences in MS susceptibility, the association should be genotype dependent i.e. the risk SNP should be associated with the quantitative trait of the phenotype.

### MS risk SNPs associated with EBV phenotypes

**Figure 3:**
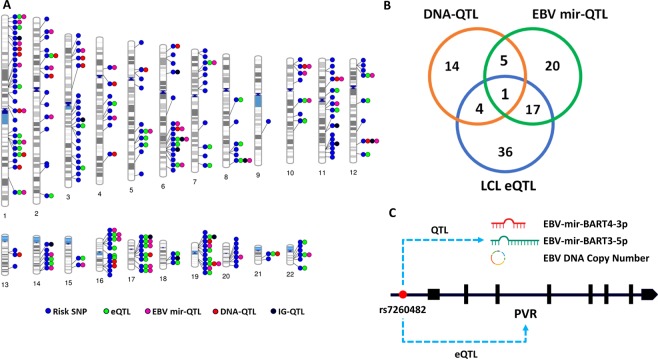
(**A**) MS risk SNP genotype effects on each trait; DNA-QTL (genotype effect on EBV DNA copy number), EBV mir-QTL (genotype effect on EBV miRNA expression level), IG-QTL (genotype effect on LCL intrinsic growth rate) and LCLeQTL (genotype effect on host genes within a 1 mega base window from the SNP). (**B**) The overlap between the MS risk SNPs which are DNA-QTLs, EBV mir-QTLs and LCLeQTLs. (**C**) The rs7260482 SNP as is associated with EBV DNA copy number, EBV miRNA expression (BART4-3p and BART3-5p) and expression level of the proximal MS risk gene (PVR).

We then interrogated the association of MS risk SNP genotypes with EBV DNA copy number (DNA-QTL), EBV miRNA expression (EBV mir-QTL), and LCL intrinsic growth rate (IG-QTL) (Fig. 3A). We had previously shown they were also over-represented amongst LCLeQTLs^13^. 24 SNPs were associated with EBV DNA copy number, 43 with EBV miRNA expression, and 58 were LCLeQTLs in this dataset (Fig. 3B and Supplementary Table 3–7). Many MS risk SNPs were associated with multiple EBV phenotypes. One SNP, rs7260482, near gene PVR, was associated with EBV DNA copy number, EBV miRNA expression (BART4-3p and BART3-5p) and PVR expression level (Fig. 3C). The highly statistically significant interaction for these three parameters suggests a pathogenic role in MS. We then sought to refine the nature of the interaction. The genotype association with expression and EBV DNA copy number was calculated through testing the differences for each trait in protective (PP), heterozygous (PR) and risk (RR) genotypes using the linear model test. Correlations were tested using Spearman’s Rank-Order correlation test.

### The PVR MS risk SNP is associated with EBV DNA copy number, EBV miRNA expression and PVR expression in LCLs

**Figure 4:**
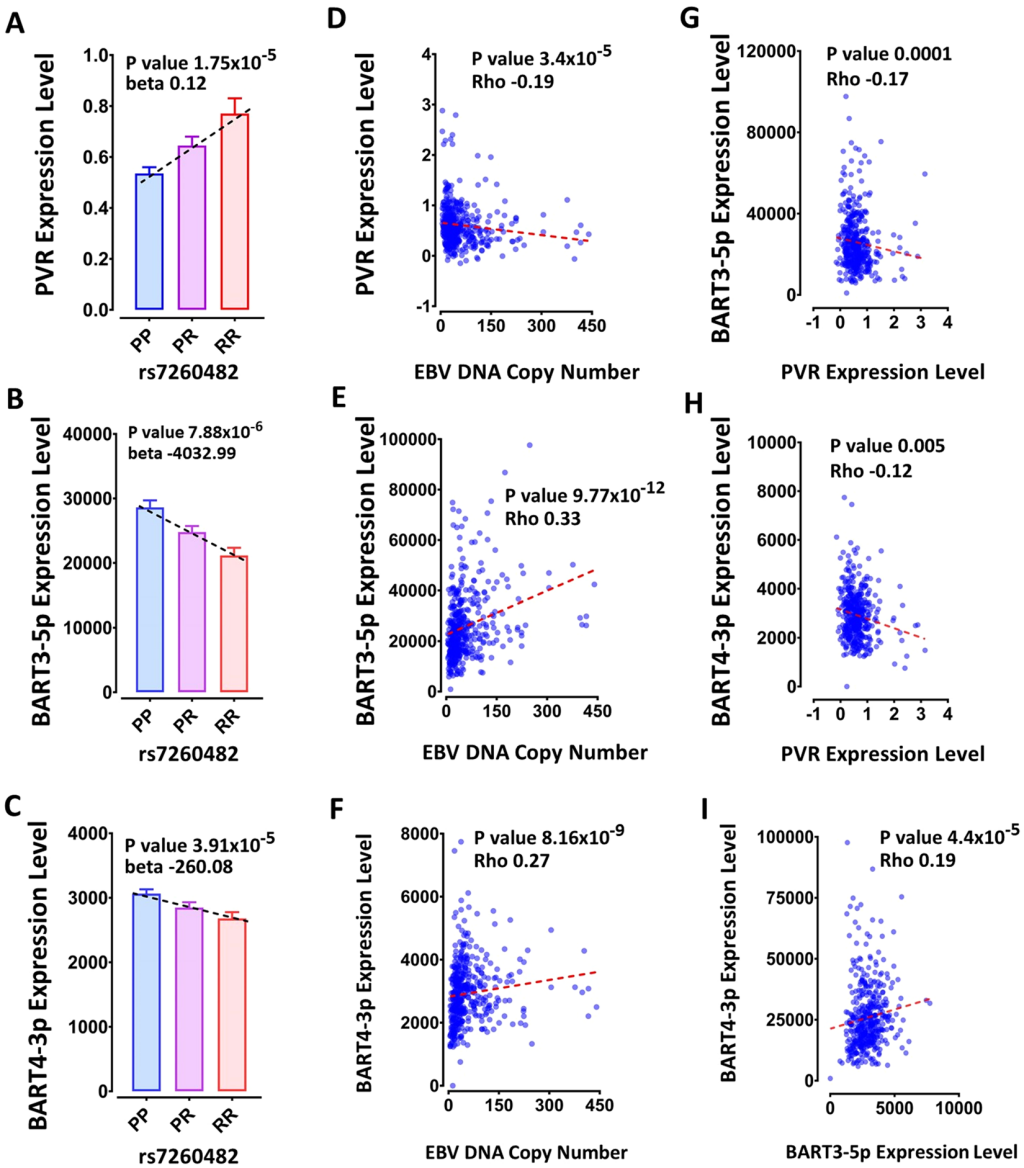
(**A**) Association of MS risk SNP rs7260482 genotype with expression of proximal gene PVR, and EBV miRNAs (**B**) BART3-5p and (**C**) BART4-3p. Correlation of EBV DNA copy number with (**D**) PVR, (**E**) BART3-5p and (**F**) BART4-3p expression levels. (**G-I**) Correlation between PVR, BART3-5p and BART4-3p expression levels. PP - protective homozygous, PR - heterozygote genotype, RR - risk homozygous SNP genotype. The genotype association with expression was calculated through testing the differences in the expression level in PP, PR and RR genotypes using the linear model test. Correlations were tested using Spearman’s Rank-Order correlation test.

**Figure 5:**
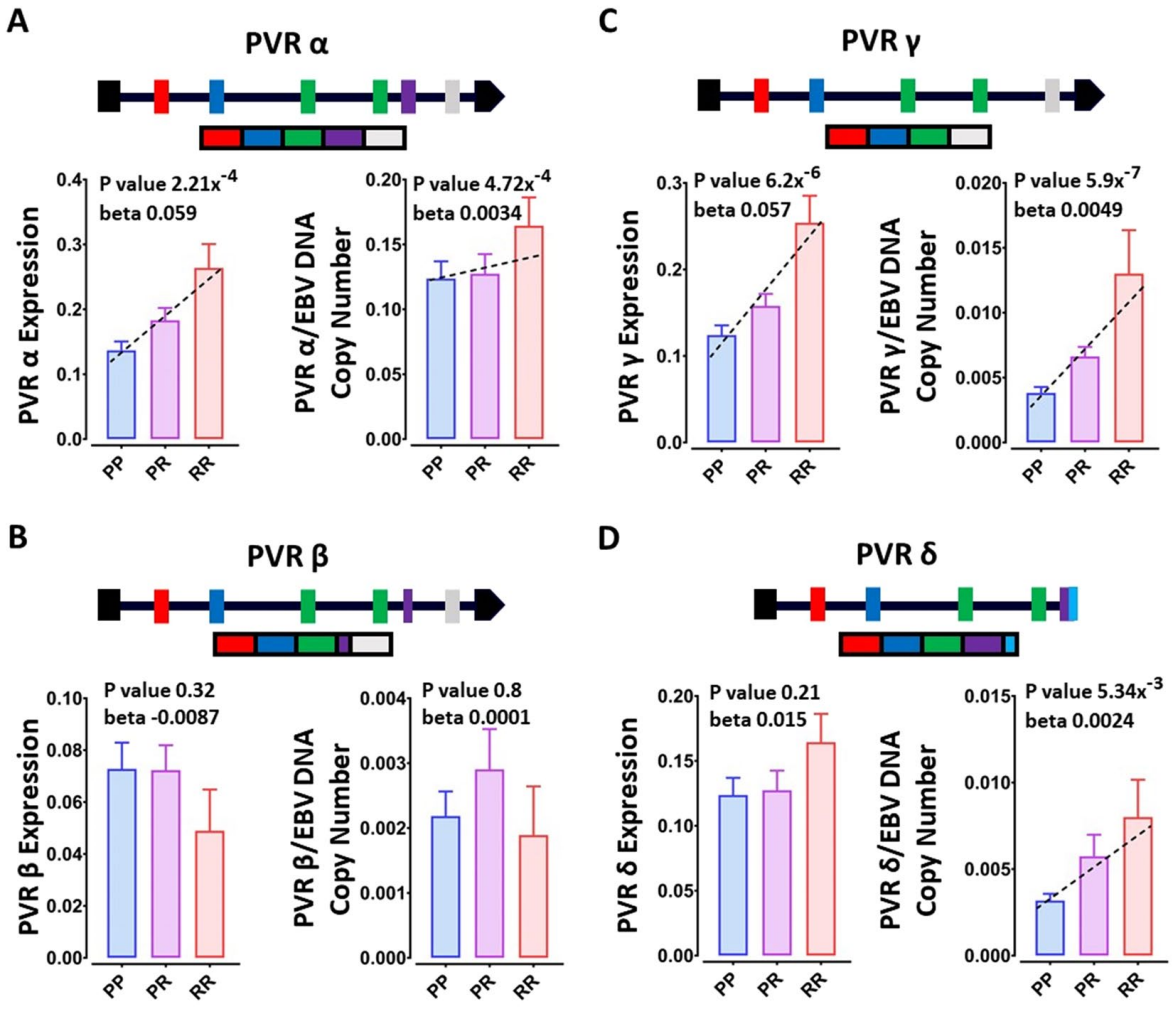
The rs7260482 SNP genotype effect on PVR isoforms expression level; PVRα (**A**), PVRβ (**B**), PVRγ (**C**) and PVRδ (**D**).

PVR expression was negatively correlated with expression of BART3-5p and BART4-3p, consistent with a possible direct role of these miRNAs in regulating PVR expression (Fig. 4). Further, the risk allele of the PVR risk SNP, rs7260482 was associated with higher expression of PVR (p = 1.75 × 10^−5^), and lower expression of EBV miRNAs BART3-5p (p = 7.88 × 10^−6^) and BART4-3p (p = 3.91 × 10^−5^), consistent with reduced regulation by these two EBV miRNAs dependent on the risk genotype i.e. BART3-5p and BART4-3p downregulation of PVR is protective. Lower PVR expression was correlated with higher EBV DNA copy number (p = 3.4 × 10^−5^, R = −0.19), as was higher BART expression, suggesting lower DNA copy number is protective in the context of LCLs. PVR has many isoforms coding for proteins with different functions^23^. From RNAseq data^16^ we determined that the rs7260482 risk allele was significantly associated with higher expression of the α (p = 2.21 × 10^−4^) and γ (p = 6.2 × 10^−6^) PVR isoforms, but not the β and δ isoforms (Fig. 5). For the risk allele, α, γ and δ isoforms were also associated with higher EBV DNA copy number. These isoforms encode both soluble and membrane bound proteins, suggesting similar roles in EBV infection for each isoform.

### Is high EBV DNA copy number protective in MS?

**Figure 6:**
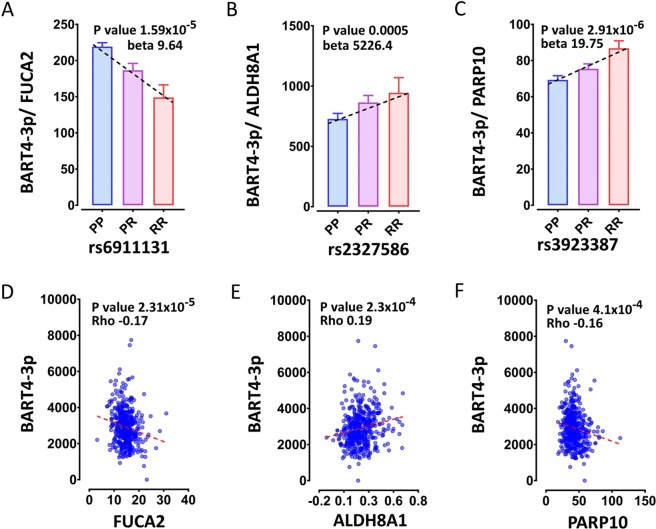
The genotype effect of MS risk SNPs may be associated with increased or decreased ratio of BART4-3p/risk gene expression (**A–C**); and the proximal risk gene may be associated with increased or decreased BART4-3p expression (**D–F**).

**Figure 7:**
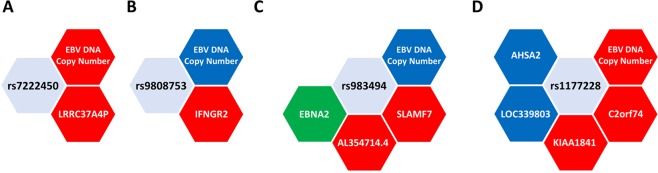
Five MS risk SNPs (including rs7260482, Fig. 3, 4) were EBV DNA-QTLs and were LCLeQTLs. (**A**) The risk allele of rs7222450 is associated with increased expression of its proximal host gene LRRC37A4P and increased EBV DNA copy number; (**B**) the risk allele of rs9808753 is associated with lower EBV DNA copy number and higher expression of its proximal host gene IFNGR2; (**C**) the risk allele of rs983494 is associated with higher expression of proximal host genes SLAMF7 and AL354714.4, lower EBV DNA copy number and is colocated with an EBNA2 binding site (green); (**D**) the risk allele of rs1177228 is associated with lower expression of its proximal host genes AHSA2 and LOC339803, higher expression of its proximal genes KIAA1841 and C2ORF74, and higher EBV DNA copy number. Red and blue show positive and negative correlations, respectively.

To determine if there was a consistent association between the risk alleles of MS risk loci with lower EBV DNA copy number and lower expression of BART3-5p and BART4-3p we investigated the genotype association of the other risk loci with each of these and with other EBV miRNAs. Overall, out of 86 MS risk SNP-EBV miRNA pairs, the risk allele was associated with lower EBV miRNA expression level in 49 cases, and higher in 37. In addition, out of 24 DNA-QTL, the risk allele was associated lower EBV DNA copy number in 14 cases, and higher in 10. The ratio of the miRNA with the risk gene expression was used to show the genotype dependency of the dynamics of interaction between MS risk genes/EBV miRNAs pairs. The risk allele was associated with higher miRNA expression of BART4-3p for 3 LCLeQTLs. For risk genes FUCA2 and PARP10 expression decreased as BART4-3p expression increased, consistent with a direct inhibition of the gene mRNAs by the EBV miRNAs (Fig. 6). The risk alleles were associated with higher expression of these genes, and resistance to BART inhibition, and with lower EBV DNA copy number. This was consistent with higher BART and lower EBV DNA copy number being protective. However, for MS risk gene ALDH8A1 expression increased with increasing BART4-3p expression, and risk allele was associated with higher BART4-3p expression and was not significant with EBV DNA copy number. Five SNPs were associated with both EBV DNA copy number and EBV miRNAs. For two, the risk allele was associated with lower EBV DNA copy number and lower expression of BART miRNAs and higher expression of BHRF1-1 miRNA. The risk allele of three were associated with higher EBV DNA copy number and higher BART3-5p and BART4-3p expression (Fig. 7). Again, this is consistent with a complex association of EBV DNA copy number with pathogenesis.

**Figure 8:**
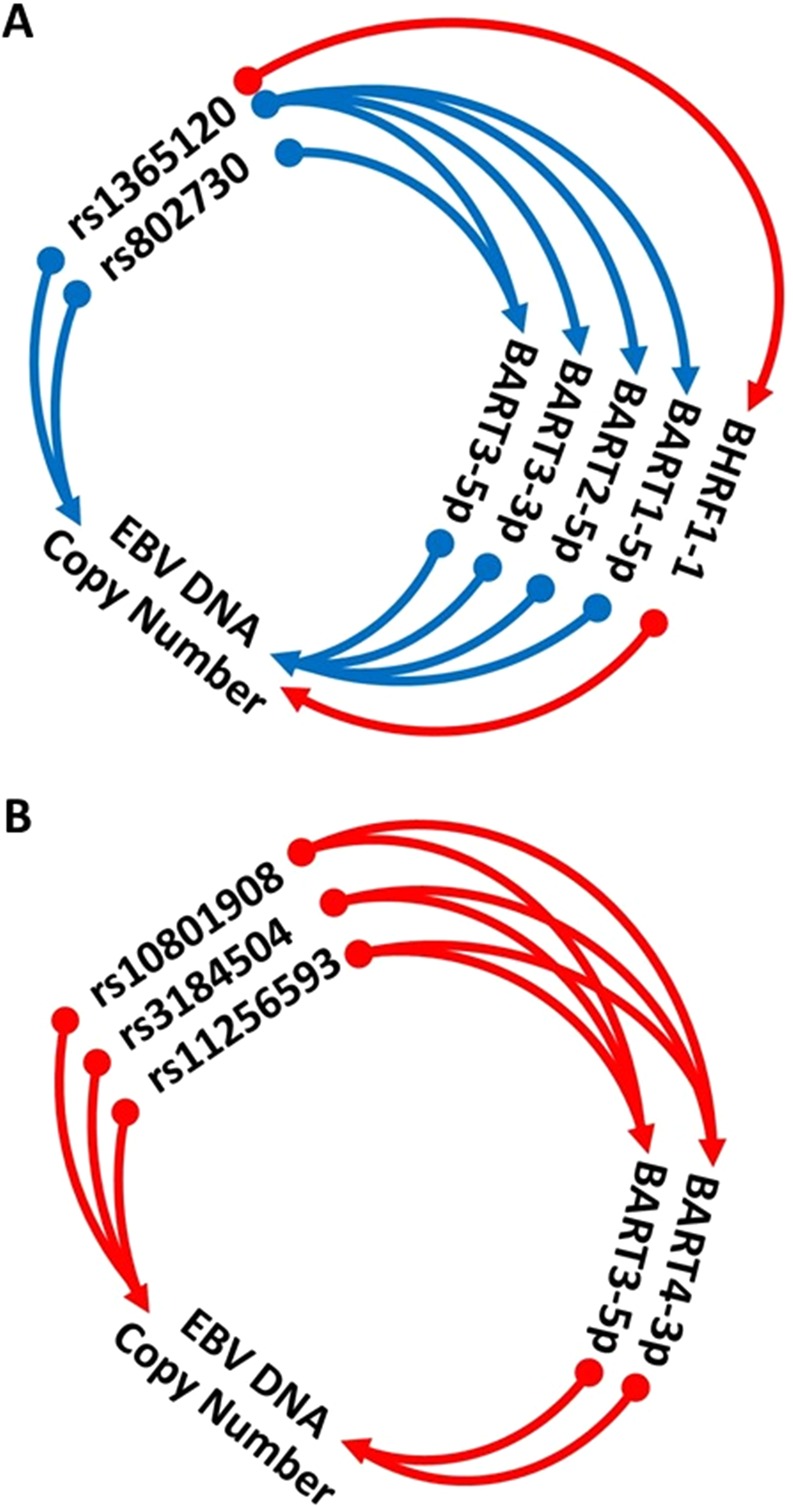
Six risk SNPs (including rs7260482, Fig. 3, 4) were both EBV miRNA-QTLs and DNA-QTLs. (**A**) SNPs rs1365120 and rs802730 were not associated with expression of their proximal genes (ie not LCLeQTLs) but the risk allele was associated with reduced EBV DNA copy number, reduced expression of four EBV miRNAs, and increased expression of one miRNA and expression of these miRNAs were correlated with EBV DNA copy number. (**B**) The risk allele of SNPs rs10801908, rs3184504 and rs11256593 were associated with higher expression of two miRNAs and higher EBV DNA copy number. Red and blue arrows show positive and negative correlations, respectively.

Four risk SNPs were associated with EBV DNA copy number and gene expression, but not EBV miRNAs. This would suggest that they are not regulated by EBV miRNAs, but are interacting with the regulation of EBV DNA copy number through their effect on risk gene expression and with other EBV host genes (Fig. 8). The risk allele is associated with higher EBV DNA copy number for two SNPs, which are associated with higher or lower expression of neighbouring genes. The risk allele for the other two is associated with lower EBV DNA copy number and higher expression of the proximal risk genes. One was also colocated with an EBNA2 binding site. Although EBV DNA copy number, IGR and EBV miRNAs were correlated with each other, particularly BART4-3p (Supplementary Fig. 1), associations of MS risk loci with IGR was below the FDR 5% threshold we have used here.

## Discussion

We found that of host genes whose expression was correlated with EBV DNA copy number in LCLs, MS and SLE risk genes were highly over-represented, whereas risk genes for diseases and traits not as associated with EBV were only slightly or not over-represented. The conditions tested were other autoimmune diseases: type 1 diabetes, rheumatoid arthritis, Crohn’s disease, inflammatory bowel diseases collectively, and Crohn’s disease and ulcerative colitis specifically; and height. Of the MS risk genes correlated with EBV DNA copy number, nearly half of their MS risk SNPs were associated with expression of EBV miRNAs and/or the proximal host genes to the risk SNPs. This suggests the function of many of these genes affects EBV phenotype, the risk SNP modifies this effect, and the EBV genes whose expression is most correlated with the expression of the risk genes, and associated with the risk SNPs, are important targets for controlling EBV in MS and SLE, and potentially other diseases driven by EBV. These are ebv-miR-BART4-3p and ebv-miR-BART5-3p. The utility of targeting these EBV miRNAs for therapy needs to be further investigated using laboratory approaches such as controlling EBV and experimental autoimmune encephalomyelitis in the humanised mouse^24^, and in LCLs from people with MS.

Disease risk SNPs for many of the above traits were over-represented among SNPs associated with proximal gene expression in LCLs (LCLeQTLs). This is likely due to the role of these SNP/genes in non-EBV specific functions that affect the diseases/traits. Such functions include energy production for proliferating/differentiating cells, contribution to cell division and controlling immune cell differentiation^13^. However, risk loci which are implicated in affecting EBV DNA copy number can be distinguished from risk loci affecting LCLs for such EBV independent processes. As these risk loci (EBV DNA-QTLs) can then be seen to be over-represented in diseases known to be affected by EBV, we can infer that the processes affecting EBV DNA copy number are likely to be important in disease. This is consistent with the finding of Harley *et al*.^14^, that EBV transcription factor EBNA2 binding sites are over-represented in several autoimmune disease risk genes. Consequently, agents affecting these processes (EBV DNA copy number, EBNA2 binding) may be of therapeutic benefit.

It is not clear if higher or lower EBV DNA copy number in LCLs is itself an indication of MS risk and progression. Increasing DNA copy number is necessary for initiation of lysis^20^, but the importance of EBV DNA copy number in latency III (as measured here), or even the importance of latency versus lysis in disease, is difficult to determine. In lysis, cells are more susceptible to immune control; but lysis is also necessary for EBV proliferation in the host, at least due to non-immortalised cell proliferation. The direction of risk allele effect is not informative here, as risk SNPs can be associated with higher or lower EBV DNA copy number compared to protective alleles.

There are no therapies currently specifically targeting EBV available in the clinic^6^. The efficacy of immune modifying therapies for MS are limited, and may substantially improve if they are combined with EBV therapies. Nucleic acid based regulation of human genes is now available in the clinic for a number of conditions^25^. We are unaware of any examples for viral control in the clinic, but viral targets are especially attractive since normal host cellular machinery can be left intact, minimising adverse reactions.

Of particular interest as a novel therapeutic target is the gene PVR, coding for the polio virus receptor. It is a protein of the immunoglobulin family with diverse roles^23^. Its expression and risk loci are very highly associated with EBV DNA copy number and the expression of EBV miRNAs BART3-5p and BART4-3p. PVR regulates cell adhesion, so aggregation and trafficking. This may be especially important in the secondary lymphoid organ B cell follicles, where EBV infected B cells may evade immune selection for deletion. This process has previously been suggested as pathogenic in MS^11^. PVR also controls cell mediated killing of infected cells. PVR is a ligand partner for the MS risk gene DNAM1 (CD226), and for TIGIT. The association between the MS risk genotype with EBV DNA copy numbers for three PVR isoforms, coding for soluble, insoluble and variable cytoplasmic domains; is supportive of a functional role for this protein in variable susceptibility to MS due to variation in host EBV immunoregulation. Laquinimod, a candidate MS therapy so far unsuccessful in clinical trials^26^, targets PVR. Although it was not designed to affect EBV infection specifically, it may be possible to make molecular alterations to it to alter its interactions with the PVR/DNAM1/TIGIT and so improve its effect on EBV. Another therapeutic possibility against EBV is the poliovirus vaccine, which has a natural tropism for PVR, and so may be useful in inhibiting its interactions.

### Future work

To further investigate the utility of targeting EBV DNA copy number it would be useful to know if LCLs generated from B cells from people with MS or SLE have different phenotypic traits compared to controls, and if transcriptomic interactions between EBV and host genomes are different. In particular, it would be interesting to know if altered EBV DNA copy number was a trait of LCLs in latency III (LCLs) and/or on induction of lysis. These data also provide in principle support for EBV control in EBV driven cancers through using these LCL EBV phenotypes as readouts of intervention success, especially if there is an excess of somatic mutations that alter the phenotypes, including their balance.

### Limitations

LCLs are generated using EBV strain B95-8, which lacks several genes, including many EBV miRNAs^27^, so that the interactions between host genes and EBV genes measured in these cell lines may be crucially different from that observed *in vivo* and in disease. Polymorphisms in viral genes can affect phenotypic outcomes^28^. Further, interactions may vary between individuals, due to different genotypes, and in LCLs generated from people with MS or SLE. However, an advantage of working with LCLs is that the utility of interventions such as EBV miRNA knockdowns can be assessed *in vitro*. They could also be assessed in the humanised mouse model of EBV. Finally, it may be that the harmful consequences of dysregulated control of EBV indicated by the susceptibility genes is a precursor to the development of harmful immortal B cell clones, whose pathogenicity is no longer dependent on EBV infection or its control. It is notable though, that titres of antibodies to EBV proteins are associated with increased and new T2 lesions, and with conversion from Clinically Isolated Syndrome to MS^29^, suggesting that control of EBV contributes to ongoing pathogenesis. Finally, there is the ‘horse has bolted’ argument, that the effect of EBV on MS susceptibility leads to development of MS, but control of EBV after diagnosis will not alter the course of disease.

## Conclusion

We believe this paper presents compelling new evidence from the interaction between MS risk loci and EBV DNA copy number that host genetic variation affects EBV infection. The over-representation of host genes associated with the EBV associated autoimmune diseases MS and SLE among these DNA-QTLs further implicates EBV in their disease pathogenesis. These data open up new approaches to controlling EBV that may be of therapeutic value. Specific molecular interactions between the expression of EBV miRNAs BART4-3p and BART3-5p, the MS risk gene PVR, and other MS risk loci with EBV DNA copy number and gene expression suggest they are useful targets for controlling EBV in MS. Our findings are consistent with an EBV susceptibility signature contributing to risk of developing MS and SLE. These data provide strong support for further investigations into targeting the implicated EBV genes and processes to treat MS and other EBV associated diseases such as SLE, and cancers.

## Methods

### Risk SNP-Gene pair eQTLs Overrepresentation

The risk SNPs lists were extracted from the latest and highest statistically powered GWAS studies for Multiple Sclerosis (MS)^4^, Systemic lupus erythematosus (SLE)^30^, Height^21^, Rheumatoid Arthritis^31^, Type 1 Diabetes (T1D)^32^, Inflammatory Bowel Disease (IBD), Ulcerative Colitis (UC) and Crohn’s Disease (CD)^33^. The GTEx portal V7^34^ was used to extract SNP-Gene pairs eQTL. The intragenic SNPs were paired with the genes they were located within, and intergenic SNPs paired with the two proximal genes, located upstream and downstream, respectively, of the SNP. In total, eQTL data for 6064731 SNP-Gene pairs were available on the GTEx portal. From those, 721884 SNP-Gene pairs were significant with p values of less than 0.05. We identified 198, 47, 599, 74, 39, 116, 69 and 110 risk SNP-Gene pairs for MS, SLE, Height, RA, T1D, IBD, UC and CD, respectively. Then we tested the overrepresentation of the significant risk SNP-Gene pair eQTLs for each disease compared to the expected significant SNP-Gene pair eQTLs in the LCL context using an exact hypergeometric probability test^35^.

### Host genes associated with EBV DNA Copy Number

The estimated EBV DNA copy number^18^ and host gene expression^16^ for 433 LCL samples were obtained. The Spearman’s rank-order correlation test was performed to test the association of 23722 host genes expression with estimated EBV DNA copy number. We then filtered out the associated genes with a false discovery rate (FDR) of less than 5% using the Benjamini Hochberg test. The final list were termed EDC genes, which comprise 1322 host genes highly associated with EBV DNA copy number. The risk gene lists for MS, SLE, Height, RA, T1D, IBD, UC and CD were obtained from related GWAS studies, in the same way as the risk SNP lists described above. Overrepresentation of the risk genes for each disease among the EDC genes were tested using an exact hypergeometric probability test^35^.

### Quantification of the EBV miRNA expression profile in the Geuvadis LCL cohort

Small RNA sequencing reads from the Geuvadis Project LCL cohort^16^ were download and mapped to EBV miRNA sequences obtained from the miRBase database^36^ using the mirdeep2 software package^37^.

### Quantitative Trait Loci calculations

In this study we measured the association of MS risk SNPs with EBV traits (DNA copy number, LCL intrinsic growth rate and EBV miRNAs expression) using a quantitative trait loci (QTL) approach. In other words, we performed linear model analyses to test the differences in effects of protective, heterozygous and risk genotypes of every single MS risk SNP on EBV traits. The MatrixEQTL R package^38^ was used for calculating the genotype effects on each trait; DNA-QTL (genotype effect on EBV DNA copy number), EBV mir-QTL (genotype effect on EBV miRNAs expression level), IG-QTL (genotype effect on LCL intrinsic growth rate) and LCL eQTL (genotype effect on host genes within a 1 mega base window from the SNP). The host gene expression was tested here as a host trait which may explain the MS risk SNPs associations with EBV traits. Gender and ethnicity were considered as covariances in the QTL analyses. From 201 MS risk SNPs, 196 SNP genotypes were available for LCL samples from 1000 genomes project phase 3^15^ and HapMap 3^39^. The LCL intrinsic growth rate for 529 genotyped LCL samples were extracted from^17^. Also, the estimated EBV DNA copy number for 1753 genotyped LCL samples were obtained^18^. The normalized and processed 23722 host genes expression data for 445 LCL samples were obtained from the Geuvadis study^16^. Then the Benjamini Hochberg test was performed to filter out the QTLs with FDR less than 5%. Then we visualized the DNA-QTL, EBV mir-QTL, IG-QTL and LCL eQTL loci as chromosomal ideogram using PhenoGram webtool^40^.

## Supplementary information

Supplementary Information

## Data Availability

The datasets generated during and/or analysed during the current study are available from the corresponding author on reasonable request.
