# Supplementary material for: The interaction of Multiple Sclerosis risk loci with Epstein-Barr virus phenotypes implicates the virus in pathogenesis
Source: Sci Rep. 2020 Jan 13;10:193. doi: 10.1038/s41598-019-55850-z (PMC6957475; doi:10.1038/s41598-019-55850-z)
Supplement: Supplementary file 1 — Supplementary Information [file 41598_2019_55850_MOESM1_ESM.pdf]

# The interaction of Multiple Sclerosis risk loci with Epstein-Barr virus phenotypes implicates the virus in pathogenesis

Ali Afrasiabi[1], Grant P. Parnell[1], Sanjay Swaminathan[1], Graeme J. Stewart[1], David R. Booth[1]*

Affiliations

[1] Centre for Immunology and Allergy Research, Westmead Institute for Medical Research, University of Sydney, Australia

* Corresponding author: Professor David R. Booth. Email: david.booth@sydney.edu.au

## Supplementary Information

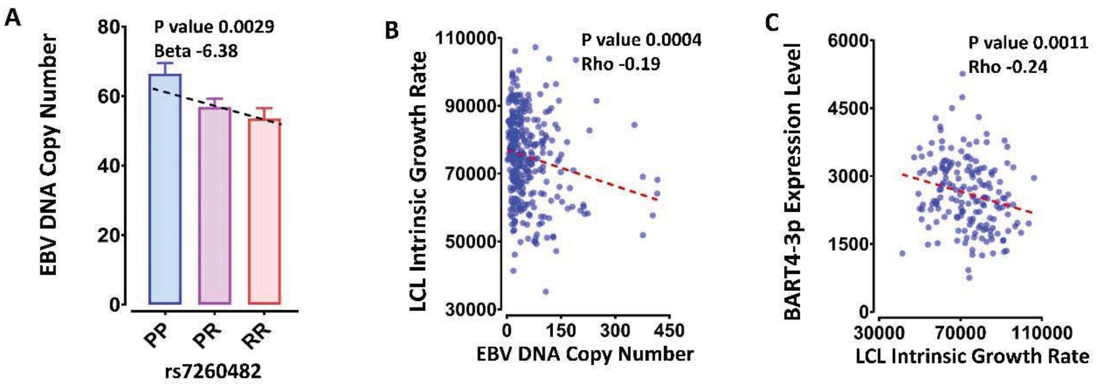


**Supplementary Figure 1:** (A) The PVR risk SNP rs7260482 genotype effect on EBV DNA copy number level. (B) EBV DNA copy number association with LCL intrinsic growth rate and (C) BART4-3p and LCL intrinsic growth rate correlation.

**Supplementary Table 1:** LCLeQTLs at FDR less than 5% for Multiple Sclerosis, Systemic Lupus Erethymatosus, Crohn's Disease, Ulcerative Colitis, Inflammatory Bowel Disease, Rheumatoid Arthritis, Type 1 Diabetes and Height. The LCLeQTLs for risk SNPs and their proximal genes were extracted from GTEx V7.

**Supplementary Table 1:** LCLeQTLs with FDR less than 5% for Multiple Sclerosis, Systemic Lupus Erethymatosus, Crohn’s Disease, Ulcerative Colitis, Inflammatory Bowel Disease, Rheumatoid Arthritis, Type 1 Diabetes and Height. The LCLeQTLs for risk SNPs and their proximal genes were extracted from GTEx V7.

| SNP (rs ID) | Variant Call Format (VCF) | Gene Name | Gene Ensembl ID | slope | pval_nominal | Disease/Trait |
|---|---|---|---|---|---|---|
| rs7954567 | 12_6491125_G_A_b37 | AC005840.1 | ENSG00000111321 | -0.235075 | 0.0454086 | Crohn’s Disease |
| rs10521318 | 16_86011337_C_T_b37 | AC092723.1 | ENSG00000269667 | -0.704635 | 0.00131011 | Crohn’s Disease |
| rs6545800 | 2_25118885_C_T_b37 | ADCY3 | ENSG00000138031 | -0.420112 | 7.22E-08 | Crohn’s Disease |
| rs7282490 | 21_45615741_G_A_b37 | AP001057.1 | ENSG00000232124 | 0.215473 | 0.031109 | Crohn’s Disease |
| rs2188962 | 5_131770805_C_T_b37 | C5orf56 | ENSG00000197536 | 0.20139 | 0.0121974 | Crohn’s Disease |
| rs10781499 | 9_139266405_G_A_b37 | CARD9 | ENSG00000187796 | -0.316039 | 0.00367951 | Crohn’s Disease |
| rs3091316 | 17_32593974_G_A_b37 | CCL2 | ENSG00000108691 | 0.343899 | 0.0225208 | Crohn’s Disease |
| rs1569723 | 20_44742064_C_A_b37 | CD40 | ENSG00000101017 | 0.121923 | 0.0272152 | Crohn’s Disease |
| rs11010067 | 10_35295431_C_G_b37 | CUL2 | ENSG00000108094 | -0.210407 | 0.00323466 | Crohn’s Disease |
| rs2155219 | 11_76299194_G_T_b37 | EMSY | ENSG00000158636 | -0.128727 | 0.0442355 | Crohn’s Disease |
| rs1363907 | 5_96252803_G_A_b37 | ERAP2 | ENSG00000164308 | 1.17758 | 2.33E-49 | Crohn’s Disease |
| rs4246215 | 11_61564299_G_T_b37 | FADS2 | ENSG00000134824 | 0.376861 | 0.000593905 | Crohn’s Disease |
| rs2231884 | 11_65656564_C_T_b37 | FIBP | ENSG00000172500 | 0.41356 | 4.57E-08 | Crohn’s Disease |
| rs9557195 | 13_99956622_T_C_b37 | GPR183 | ENSG00000169508 | 0.195307 | 0.0280524 | Crohn’s Disease |
| rs7773324 | 6_382559_G_A_b37 | IRF4 | ENSG00000137265 | 0.209586 | 0.045481 | Crohn’s Disease |
| rs864745 | 7_28180556_T_C_b37 | JAZF1 | ENSG00000153814 | -0.315652 | 0.00459772 | Crohn’s Disease |
| rs3764147 | 13_44457925_A_G_b37 | LACC1 | ENSG00000179630 | -0.730002 | 6.47E-08 | Crohn’s Disease |
| rs6863411 | 5_141513204_A_T_b37 | NDFIP1 | ENSG00000131507 | 0.333268 | 0.000380645 | Crohn’s Disease |
| rs1728918 | 2_27635463_A_G_b37 | NRBP1 | ENSG00000115216 | -0.21719 | 0.0309303 | Crohn’s Disease |
| rs16967103 | 15_38899190_T_C_b37 | RASGRP1 | ENSG00000172575 | 0.387154 | 0.00201659 | Crohn’s Disease |
| rs670523 | 1_155878732_A_G_b37 | RIT1 | ENSG00000143622 | 0.119933 | 0.010309 | Crohn’s Disease |
| rs1569723 | 20_44742064_C_A_b37 | RPL13P2 | ENSG00000213820 | -0.313917 | 0.0365064 | Crohn’s Disease |
| rs10486483 | 7_26892440_G_A_b37 | SKAP2 | ENSG00000005020 | 0.232077 | 0.0286064 | Crohn’s Disease |
| rs6716753 | 2_231097129_T_C_b37 | SP140 | ENSG00000079263 | -0.645479 | 8.33E-10 | Crohn’s Disease |
| rs1517352 | 2_191931464_A_C_b37 | STAT4 | ENSG00000138378 | -0.241904 | 0.0327836 | Crohn’s Disease |
| rs9525625 | 13_43018030_T_C_b37 | TNFSF11 | ENSG00000120659 | -0.387095 | 0.000068 | Crohn’s Disease |
| rs9557195 | 13_99956622_T_C_b37 | UBAC2 | ENSG00000134882 | 0.295694 | 0.00703947 | Crohn’s Disease |
| rs1819333 | 6_167373547_T_G_b37 | Z94721.1 | ENSG00000227598 | 0.402163 | 0.00110725 | Crohn’s Disease |
| rs10761659 | 10_64445564_A_G_b37 | ZNF365 | ENSG00000138311 | -0.348324 | 0.00617857 | Crohn’s Disease |
| rs12946510 | 17_37912377_C_T_b37 | RP11-94L15.2 | ENSG00000264198 | 0.391955 | 0.000000449 | Crohn’s Disease |
| rs6988484 | 8_49413780_T_C_b37 | AC026904.1 | ENSG00000253140 | -0.44372 | 0.000217519 | Height |
| rs17081935 | 4_57823476_C_T_b37 | AC069307.1 | ENSG00000269949 | 0.481958 | 0.00182777 | Height |
| rs1797625 | 3_112826415_A_T_b37 | AC078785.1 | ENSG00000240057 | -0.509043 | 0.000126934 | Height |
| rs7162542 | 15_84514290_C_G_b37 | ADAMTSL3 | ENSG00000156218 | 0.257139 | 0.00726289 | Height |
| rs2247870 | 5_90151589_G_A_b37 | ADGRV1 | ENSG00000164199 | -0.252581 | 0.0413482 | Height |
| rs2857693 | 6_31588384_G_T_b37 | AIF1 | ENSG00000204472 | 0.395949 | 0.000913274 | Height |
| rs798497 | 7_2795957_A_G_b37 | AMZ1 | ENSG00000174945 | 0.405068 | 0.00224231 | Height |
| rs4974480 | 3_134178562_A_T_b37 | ANAPC13 | ENSG00000129055 | 0.698472 | 1.28E-20 | Height |
| rs12347744 | 9_97575273_C_T_b37 | AOPEP | ENSG00000148120 | 0.705736 | 0.000469931 | Height |
| rs17807185 | 7_77308295_A_G_b37 | APTR | ENSG00000214293 | 0.29817 | 0.00231921 | Height |
| rs10767838 | 11_30347927_A_G_b37 | ARL14EP | ENSG00000152219 | -0.654813 | 2.77E-14 | Height |
| rs4803468 | 19_41922352_A_G_b37 | BCKDHA | ENSG00000248098 | -0.231178 | 0.0418478 | Height |
| rs1341278 | 6_81038921_T_G_b37 | BCKDHB | ENSG00000083123 | 0.555541 | 0.0104101 | Height |
| rs2164747 | 12_104344836_A_G_b37 | C12orf73 | ENSG00000204954 | -0.363374 | 0.00164299 | Height |
| rs227724 | 17_54778817_A_T_b37 | C17orf67 | ENSG00000214226 | -0.213033 | 0.0495165 | Height |
| rs897080 | 2_44774202_C_T_b37 | CAMKMT | ENSG00000143919 | -0.442176 | 0.00328557 | Height |
| rs5757318 | 22_39275656_A_T_b37 | CBX6 | ENSG00000183741 | -0.300249 | 0.0321886 | Height |
| rs915506 | 10_97805074_G_A_b37 | CCNJ | ENSG00000107443 | 0.260063 | 0.00214091 | Height |
| rs7985356 | 13_115027462_T_A_b37 | CDC16 | ENSG00000130177 | 0.48776 | 0.000000882 | Height |
| rs42039 | 7_92244422_C_T_b37 | CDK6 | ENSG00000105810 | -0.235807 | 0.0276284 | Height |
| rs1582931 | 5_122657199_G_A_b37 | CEP120 | ENSG00000168944 | -0.286248 | 0.0144635 | Height |
| rs692964 | 18_13094132_G_A_b37 | CEP192 | ENSG00000101639 | -0.597417 | 7.54E-14 | Height |
| rs11648796 | 16_792190_A_G_b37 | CIAO3 | ENSG00000103245 | 0.328276 | 0.0211296 | Height |
| rs13113518 | 4_56399648_T_C_b37 | CLOCK | ENSG00000134852 | 0.240896 | 0.00721302 | Height |
| rs989393 | 9_101743336_T_C_b37 | COL15A1 | ENSG00000204291 | -0.383149 | 0.015856 | Height |
| rs2046158 | 1_86330770_C_T_b37 | COL24A1 | ENSG00000171502 | -0.280457 | 0.0470829 | Height |
| rs209918 | 1_40777842_A_G_b37 | COL9A2 | ENSG00000049089 | -0.426146 | 0.000153154 | Height |
| rs12474201 | 2_46921285_G_A_b37 | CRIPT | ENSG00000119878 | 0.285032 | 0.00465152 | Height |
| rs8052560 | 16_88777242_C_A_b37 | CTU2 | ENSG00000174177 | 0.257397 | 0.0116665 | Height |
| rs12871822 | 13_49201040_T_G_b37 | CYSLTR2 | ENSG00000152207 | 0.295634 | 0.00788042 | Height |
| rs11677466 | 2_232982257_A_T_b37 | DIS3L2 | ENSG00000144535 | -0.330453 | 0.0202704 | Height |
| rs4746769 | 10_70196580_T_C_b37 | DNA2 | ENSG00000138346 | -0.189464 | 0.00117241 | Height |
| rs6694089 | 1_172083881_G_A_b37 | DNM3 | ENSG00000197959 | -0.24525 | 0.0220592 | Height |
| rs11880992 | 19_2176403_G_A_b37 | DOT1L | ENSG00000104885 | 0.142089 | 0.026586 | Height |
| rs10283100 | 8_120596023_A_G_b37 | ENPP2 | ENSG00000136960 | -0.384817 | 0.0407997 | Height |
| rs6921207 | 6_131327956_G_A_b37 | EPB41L2 | ENSG00000079819 | 0.351359 | 0.000600815 | Height |
| rs10843390 | 12_29496991_C_T_b37 | ERGIC2 | ENSG00000087502 | -0.266248 | 0.000634067 | Height |
| rs6838153 | 4_122720999_A_G_b37 | EXOSC9 | ENSG00000123737 | 0.587588 | 3.76E-14 | Height |
| rs9766 | 17_40852841_G_A_b37 | EZH1 | ENSG00000108799 | 0.137863 | 0.0253632 | Height |
| rs3958122 | 4_1693931_C_T_b37 | FAM53A | ENSG00000174137 | -0.325189 | 0.0156816 | Height |
| rs17330192 | 6_17589375_T_C_b37 | FAM8A1 | ENSG00000137414 | -0.153455 | 0.0465033 | Height |
| rs11861084 | 16_89875710_A_C_b37 | FANCA | ENSG00000187741 | -0.197749 | 0.000704447 | Height |
| rs7154721 | 14_92427348_T_C_b37 | FBLN5 | ENSG00000140092 | 0.312144 | 0.0203946 | Height |
| rs6658763 | 1_146692373_C_T_b37 | FMO5 | ENSG00000131781 | 0.426937 | 0.039964 | Height |
| rs10401193 | 19_19591066_A_G_b37 | GATAD2A | ENSG00000167491 | 0.280096 | 0.0187228 | Height |
| rs6879260 | 5_179731014_T_C_b37 | GFPT2 | ENSG00000131459 | 0.263558 | 0.039312 | Height |
| rs798497 | 7_2795957_A_G_b37 | GNA12 | ENSG00000146535 | 0.862417 | 3.24E-15 | Height |
| rs389663 | 6_117868051_T_C_b37 | GOPC | ENSG00000047932 | 0.26303 | 0.00623981 | Height |
| rs6439168 | 3_129050943_A_G_b37 | H1FX-AS1 | ENSG00000206417 | 0.38648 | 0.00443865 | Height |
| rs9434723 | 1_9292282_G_A_b37 | H6PD | ENSG00000049239 | -0.222175 | 0.0258611 | Height |
| rs7870753 | 9_99201585_A_G_b37 | HABP4 | ENSG00000130956 | 0.545146 | 0.00000907 | Height |
| rs9404952 | 6_29804165_G_A_b37 | HLA-G | ENSG00000204632 | 0.298146 | 0.00797475 | Height |
| rs8017130 | 14_23759156_A_G_b37 | HOMEZ | ENSG00000215271 | 0.322496 | 0.00409023 | Height |
| rs3800461 | 6_34616322_G_C_b37 | ILRUN | ENSG00000196821 | 0.375143 | 0.00941159 | Height |
| rs1346490 | 19_7244233_A_C_b37 | INSR | ENSG00000171105 | 0.198382 | 0.0440386 | Height |
| rs552707 | 7_28205303_T_C_b37 | JAZF1 | ENSG00000153814 | -0.386706 | 0.00323706 | Height |
| rs2211866 | 21_39688107_A_G_b37 | KCNJ15 | ENSG00000157551 | 0.270562 | 0.00445067 | Height |
| rs6137287 | 20_21180259_C_T_b37 | KIZ | ENSG00000088970 | 0.318593 | 0.00294931 | Height |
| rs1415701 | 6_130345835_G_A_b37 | L3MBTL3 | ENSG00000198945 | -0.215031 | 0.0498333 | Height |
| rs7740107 | 6_130374461_T_A_b37 | L3MBTL3 | ENSG00000198945 | -0.588153 | 0.000002 | Height |
| rs12871822 | 13_49201040_T_G_b37 | LINC00462 | ENSG00000233610 | 0.356594 | 0.000162995 | Height |
| rs4973429 | 2_232377818_G_T_b37 | LINC00471 | ENSG00000181798 | -0.802434 | 4.79E-17 | Height |
| rs6594336 | 5_108073085_T_C_b37 | LINC01023 | ENSG00000272523 | -0.389393 | 0.000266432 | Height |
| rs6887276 | 5_127378294_C_G_b37 | LINC01184 | ENSG00000245937 | -0.437332 | 0.000101145 | Height |
| rs12597498 | 16_990815_C_T_b37 | LMF1 | ENSG00000103227 | 0.501526 | 0.00000389 | Height |
| rs2806561 | 1_23504795_A_G_b37 | LUZP1 | ENSG00000169641 | -0.162585 | 0.0247237 | Height |
| rs7544462 | 1_37962756_A_C_b37 | MEAF6 | ENSG00000163875 | -1.01088 | 0.00000194 | Height |
| rs11616067 | 12_116393174_A_G_b37 | MED13L | ENSG00000123066 | 0.22758 | 0.0473953 | Height |
| rs12987566 | 2_172152646_C_T_b37 | METTL8 | ENSG00000123600 | -0.255729 | 0.00568283 | Height |
| rs429433 | 8_8747894_A_G_b37 | MFHAS1 | ENSG00000147324 | -0.572216 | 0.0187846 | Height |
| rs6420435 | 16_82184201_A_C_b37 | MPHOSPH6 | ENSG00000135698 | -0.397423 | 0.000309106 | Height |
| rs12323101 | 13_33143406_G_A_b37 | N4BP2L2 | ENSG00000244754 | 0.203943 | 0.00142821 | Height |
| rs4624820 | 5_141681788_G_A_b37 | NDFIP1 | ENSG00000131507 | 0.18034 | 0.0262689 | Height |
| rs1950500 | 14_24830850_T_C_b37 | NFATC4 | ENSG00000100968 | 0.214925 | 0.0286128 | Height |
| rs301901 | 5_37046626_A_G_b37 | NIPBL | ENSG00000164190 | -0.120936 | 0.00800213 | Height |
| rs4605213 | 17_49244747_G_C_b37 | NME2 | ENSG00000243678 | -0.382978 | 0.000000328 | Height |
| rs6435143 | 2_203194256_A_C_b37 | NOP58 | ENSG00000055044 | 0.122414 | 0.0186173 | Height |
| rs540652 | 2_169707428_C_T_b37 | NOSTRIN | ENSG00000163072 | -0.463338 | 0.0000759 | Height |
| rs757081 | 11_17351683_C_G_b37 | NUCB2 | ENSG00000070081 | -0.491796 | 0.0000151 | Height |
| rs7701414 | 5_131585958_A_G_b37 | P4HA2 | ENSG00000072682 | 0.357266 | 0.00199823 | Height |
| rs6911389 | 6_144079629_T_G_b37 | PHACTR2 | ENSG00000112419 | -0.284885 | 0.0149025 | Height |
| rs2425163 | 20_34432670_A_G_b37 | PHF20 | ENSG00000025293 | -0.157147 | 0.0441019 | Height |
| rs11783655 | 8_145037573_T_A_b37 | PLEC | ENSG00000178209 | -0.388741 | 0.00000894 | Height |
| rs11880124 | 19_42683791_A_G_b37 | POU2F2 | ENSG00000028277 | -0.253002 | 0.0341035 | Height |
| rs6920372 | 6_109723939_G_A_b37 | PPIL6 | ENSG00000185250 | 0.273249 | 0.0382296 | Height |
| rs4350272 | 10_25056118_A_G_b37 | PRTFDC1 | ENSG00000099256 | -0.29577 | 0.0312686 | Height |
| rs7849585 | 9_139111870_G_T_b37 | QSOX2 | ENSG00000165661 | 0.346387 | 0.000262107 | Height |
| rs8058684 | 16_53515118_G_A_b37 | RBL2 | ENSG00000103479 | -0.174083 | 0.012522 | Height |
| rs2306596 | 4_39343940_C_A_b37 | RFC1 | ENSG00000035928 | 0.167418 | 0.0193148 | Height |

| | | | | | | |
|---|---|---|---|---|---|---|
| rs2581830 | 3_53134098_T_C_b37 | RFT1 | ENSG00000163933 | -0.24716 | 0.000049 | Height |
| rs926438 | 1_25753638_C_T_b37 | RHCE | ENSG00000188672 | 0.455759 | 0.00000387 | Height |
| rs7853235 | 9_86660782_T_C_b37 | RMI1 | ENSG00000178966 | 0.140577 | 0.00445419 | Height |
| rs12882130 | 14_103878774_C_G_b37 | RPL10AP1 | ENSG00000244691 | -0.468199 | 0.000305688 | Height |
| rs1562975 | 4_109408608_G_A_b37 | RPL34-AS1 | ENSG00000234492 | 0.244121 | 0.0496918 | Height |
| rs6600365 | 1_41556253_C_T_b37 | SCMH1 | ENSG00000010803 | 0.283309 | 0.0021492 | Height |
| rs11799609 | 1_243618317_G_T_b37 | SDCCAG8 | ENSG00000054282 | 0.455752 | 0.0000145 | Height |
| rs1171615 | 10_61469090_C_T_b37 | SLC16A9 | ENSG00000165449 | 0.250995 | 0.0361003 | Height |
| rs2815379 | 1_67510474_A_G_b37 | SLC35D1 | ENSG00000116704 | -0.277793 | 0.00124179 | Height |
| rs11618507 | 13_30172751_G_T_b37 | SLC7A1 | ENSG00000139514 | -0.255865 | 0.00113959 | Height |
| rs10883563 | 10_102684380_C_A_b37 | SLF2 | ENSG00000119906 | -0.392076 | 0.0000202 | Height |
| rs11047239 | 12_24207780_C_G_b37 | SOX5 | ENSG00000134532 | -0.296592 | 0.0202086 | Height |
| rs3763631 | 9_35808334_C_G_b37 | SPAG8 | ENSG00000137098 | 0.246067 | 0.0309862 | Height |
| rs3782089 | 11_65336819_C_T_b37 | SSSCA1-AS1 | ENSG00000260233 | 0.539365 | 0.00883504 | Height |
| rs17806888 | 3_67416322_T_C_b37 | SUCLG2 | ENSG00000172340 | -0.3717 | 0.0459535 | Height |
| rs7899004 | 10_104341435_T_C_b37 | SUFU | ENSG00000107882 | 0.349001 | 0.000002 | Height |
| rs318095 | 17_46974734_T_C_b37 | SUMO2P17 | ENSG00000248278 | 0.894857 | 7.27E-14 | Height |
| rs10948222 | 6_45244415_T_C_b37 | SUPT3H | ENSG00000196284 | -0.527921 | 0.0000105 | Height |
| rs7534365 | 1_149876124_T_C_b37 | SV2A | ENSG00000159164 | -0.309404 | 0.0168661 | Height |
| rs6485978 | 11_12678415_T_C_b37 | TEAD1 | ENSG00000187079 | 0.245514 | 0.015111 | Height |
| rs992157 | 2_219154781_G_A_b37 | TMBIM1 | ENSG00000135926 | 0.37081 | 0.000000414 | Height |
| rs929637 | 7_12276522_G_T_b37 | TMEM106B | ENSG00000106460 | -0.232343 | 0.0135494 | Height |
| rs960006 | 16_4911195_T_C_b37 | UBN1 | ENSG00000118900 | 0.130442 | 0.0285296 | Height |
| rs2028067 | 17_30239698_T_C_b37 | UTP6 | ENSG00000108651 | -0.391143 | 0.00000409 | Height |
| rs9816693 | 3_38047954_G_C_b37 | VILL | ENSG00000136059 | 0.283727 | 0.0190744 | Height |
| rs199515 | 17_44856641_G_C_b37 | WNT3 | ENSG00000108379 | -0.518914 | 0.000141498 | Height |
| rs3790086 | 16_69887707_C_G_b37 | WWP2 | ENSG00000198373 | 0.224784 | 0.00802291 | Height |
| rs11779459 | 8_123980551_C_T_b37 | ZHX2 | ENSG00000178764 | -0.177862 | 0.028252 | Height |
| rs7870753 | 9_99201585_A_G_b37 | ZNF367 | ENSG00000165244 | 0.196951 | 0.0160556 | Height |
| rs4953951 | 2_136187345_C_T_b37 | ZRANB3 | ENSG00000121988 | -0.446376 | 0.000770343 | Height |
| rs8103068 | 19_17522869_T_C_b37 | CTD-2521M24.9 | ENSG00000269640 | -0.701352 | 4.6E-13 | Height |
| rs10521318 | 16_86011337_C_T_b37 | AC092723.1 | ENSG00000269667 | -0.704635 | 0.00131011 | Inflammatory Bowel Disease |
| rs6545800 | 2_25118885_C_T_b37 | ADCY3 | ENSG00000138031 | -0.420112 | 7.22E-08 | Inflammatory Bowel Disease |
| rs798502 | 7_2789880_A_C_b37 | AMZ1 | ENSG00000174945 | 0.443672 | 0.000804977 | Inflammatory Bowel Disease |
| rs7282490 | 21_45615741_G_A_b37 | AP001057.1 | ENSG00000232124 | 0.215473 | 0.031109 | Inflammatory Bowel Disease |
| rs2188962 | 5_131770805_C_T_b37 | C5orf56 | ENSG00000197536 | 0.20139 | 0.0121974 | Inflammatory Bowel Disease |
| rs10781499 | 9_139266405_G_A_b37 | CARD9 | ENSG00000187796 | -0.316039 | 0.00367951 | Inflammatory Bowel Disease |
| rs3091316 | 17_32593974_G_A_b37 | CCL2 | ENSG00000108691 | 0.343899 | 0.0225208 | Inflammatory Bowel Disease |
| rs727088 | 18_67530439_G_A_b37 | CD226 | ENSG00000150637 | 0.331287 | 0.00168229 | Inflammatory Bowel Disease |
| rs1569723 | 20_44742064_C_A_b37 | CD40 | ENSG00000101017 | 0.121923 | 0.0272152 | Inflammatory Bowel Disease |
| rs7240004 | 18_46395022_A_G_b37 | CTIF | ENSG00000134030 | -0.24535 | 0.0228475 | Inflammatory Bowel Disease |
| rs11010067 | 10_35295431_C_G_b37 | CUL2 | ENSG00000108094 | -0.210407 | 0.00323466 | Inflammatory Bowel Disease |
| rs2930047 | 5_10695526_T_C_b37 | DAP | ENSG00000112977 | -0.527924 | 0.000000165 | Inflammatory Bowel Disease |
| rs2155219 | 11_76299194_G_T_b37 | EMSY | ENSG00000158636 | -0.128727 | 0.0442355 | Inflammatory Bowel Disease |
| rs1363907 | 5_96252803_G_A_b37 | ERAP2 | ENSG00000164308 | 1.17758 | 2.33E-49 | Inflammatory Bowel Disease |
| rs4246215 | 11_61564299_G_T_b37 | FADS2 | ENSG00000134824 | 0.376861 | 0.000593905 | Inflammatory Bowel Disease |
| rs798502 | 7_2789880_A_C_b37 | GNA12 | ENSG00000146535 | 0.894658 | 1.41E-16 | Inflammatory Bowel Disease |
| rs9557195 | 13_99956622_T_C_b37 | GPR183 | ENSG00000169508 | 0.195307 | 0.0280524 | Inflammatory Bowel Disease |
| rs6927022 | 6_32612397_A_G_b37 | HLA-DQA1 | ENSG00000196735 | -0.770393 | 1.58E-14 | Inflammatory Bowel Disease |
| rs12103 | 1_1247494_T_C_b37 | INTS11 | ENSG00000127054 | -0.326775 | 0.000601023 | Inflammatory Bowel Disease |
| rs3764147 | 13_44457925_A_G_b37 | LACC1 | ENSG00000179630 | -0.730002 | 6.47E-08 | Inflammatory Bowel Disease |
| rs6863411 | 5_141513204_A_T_b37 | NDFIP1 | ENSG00000131507 | 0.333268 | 0.000380645 | Inflammatory Bowel Disease |
| rs1728918 | 2_27635463_A_G_b37 | NRBP1 | ENSG00000115216 | -0.21719 | 0.0309303 | Inflammatory Bowel Disease |
| rs35675666 | 1_8021973_G_T_b37 | PARK7 | ENSG00000116288 | 0.163399 | 0.032923 | Inflammatory Bowel Disease |
| rs1569723 | 20_44742064_C_A_b37 | RPL13P2 | ENSG00000213820 | -0.313917 | 0.0365064 | Inflammatory Bowel Disease |
| rs6716753 | 2_231097129_T_C_b37 | SP140 | ENSG00000079263 | -0.645479 | 8.33E-10 | Inflammatory Bowel Disease |
| rs1517352 | 2_191931464_A_C_b37 | STAT4 | ENSG00000138378 | -0.241904 | 0.0327836 | Inflammatory Bowel Disease |
| rs2382817 | 2_219151218_A_C_b37 | TMBIM1 | ENSG00000135926 | 0.433986 | 7.71E-12 | Inflammatory Bowel Disease |
| rs9557195 | 13_99956622_T_C_b37 | UBAC2 | ENSG00000134882 | 0.295694 | 0.00703947 | Inflammatory Bowel Disease |
| rs1819333 | 6_167373547_T_G_b37 | Z94721.1 | ENSG00000227598 | 0.402163 | 0.00110725 | Inflammatory Bowel Disease |
| rs10761659 | 10_64445564_A_G_b37 | ZNF365 | ENSG00000138311 | -0.348324 | 0.00617857 | Inflammatory Bowel Disease |
| rs12946510 | 17_37912377_C_T_b37 | RP11-94L15.2 | ENSG00000264198 | 0.391955 | 0.000000449 | Inflammatory Bowel Disease |
| rs2546890 | 5_158759900_A_G_b37 | AC008691.1 | ENSG00000249738 | -0.230585 | 0.0112089 | Multiple Sclerosis |
| rs35703946 | 16_86021505_G_A_b37 | AC092723.1 | ENSG00000269667 | -0.383622 | 0.012357 | Multiple Sclerosis |
| rs11125803 | 2_25052177_C_T_b37 | ADCY3 | ENSG00000138031 | 0.215132 | 0.0180555 | Multiple Sclerosis |
| rs2705616 | 4_87862396_C_G_b37 | AFF1 | ENSG00000172493 | 0.10828 | 0.0468428 | Multiple Sclerosis |
| rs12722559 | 10_6070273_C_A_b37 | AL137186.1 | ENSG00000229664 | -0.420687 | 0.0110543 | Multiple Sclerosis |
| rs4940730 | 18_56269737_A_G_b37 | ALPK2 | ENSG00000198796 | -0.19414 | 0.0225689 | Multiple Sclerosis |
| rs9308424 | 1_212877776_G_A_b37 | BATF3 | ENSG00000123685 | 0.216271 | 0.0220797 | Multiple Sclerosis |
| rs35486093 | 1_85729820_A_G_b37 | C1orf52 | ENSG00000162642 | 0.276227 | 0.0443953 | Multiple Sclerosis |
| rs138433213 | 3_112693983_T_G_b37 | CD200R1 | ENSG00000163606 | -1.23071 | 0.00612609 | Multiple Sclerosis |
| rs7977720 | 12_9866349_C_T_b37 | CLECL1 | ENSG00000184293 | 0.335632 | 0.00149976 | Multiple Sclerosis |
| rs5756405 | 22_37310954_A_G_b37 | CSF2RB | ENSG00000100368 | 0.268548 | 0.00469532 | Multiple Sclerosis |
| rs62013236 | 15_79247482_C_T_b37 | CTSH | ENSG00000103811 | -0.632411 | 0.0000497 | Multiple Sclerosis |
| rs9591325 | 13_50811220_T_C_b37 | DLEU1 | ENSG00000176124 | -0.429962 | 0.000133024 | Multiple Sclerosis |
| rs2327586 | 6_135495226_C_T_b37 | HBS1L | ENSG00000112339 | 0.200862 | 0.0351355 | Multiple Sclerosis |
| rs10951154 | 7_27135314_C_T_b37 | HOTAIRM1 | ENSG00000233429 | -0.258235 | 0.0320297 | Multiple Sclerosis |
| rs9909593 | 17_37970149_A_G_b37 | IKZF3 | ENSG00000161405 | 0.222279 | 0.00265836 | Multiple Sclerosis |
| rs2331964 | 3_121542898_T_C_b37 | IQCB1 | ENSG00000173226 | 0.726044 | 1.39E-11 | Multiple Sclerosis |
| rs4728142 | 7_128573967_G_A_b37 | IRF5 | ENSG00000128604 | 0.179553 | 0.0266785 | Multiple Sclerosis |
| rs2084007 | 5_133891282_C_T_b37 | JADE2 | ENSG00000043143 | -0.41808 | 0.000000232 | Multiple Sclerosis |
| rs28834106 | 19_10592144_T_C_b37 | KEAP1 | ENSG00000079999 | 0.193755 | 0.016468 | Multiple Sclerosis |
| rs75191738 | 6_130348257_C_T_b37 | L3MBTL3 | ENSG00000198945 | -0.373237 | 0.0135273 | Multiple Sclerosis |
| rs4896153 | 6_135833463_T_A_b37 | LINC00271 | ENSG00000231028 | 0.349361 | 0.0156848 | Multiple Sclerosis |
| rs116899835 | 14_88523488_C_T_b37 | LINC01146 | ENSG00000258867 | 0.430294 | 0.018489 | Multiple Sclerosis |
| rs6072343 | 20_39968188_G_A_b37 | LPIN3 | ENSG00000132793 | -0.382798 | 0.0495398 | Multiple Sclerosis |
| rs10936602 | 3_169536637_T_C_b37 | LRRIQ4 | ENSG00000188306 | 0.315228 | 0.0147709 | Multiple Sclerosis |
| rs9610458 | 22_22205353_C_T_b37 | MAPK1 | ENSG00000100030 | -0.193087 | 0.0239345 | Multiple Sclerosis |
| rs11542663 | 6_119215402_A_C_b37 | MCM9 | ENSG00000111877 | 0.332026 | 0.00144306 | Multiple Sclerosis |
| rs4808760 | 19_18301979_C_G_b37 | MPV17L2 | ENSG00000254858 | 0.153859 | 0.0207872 | Multiple Sclerosis |
| rs1801133 | 1_11856378_G_A_b37 | MTHFR | ENSG00000177000 | -0.276912 | 0.0101752 | Multiple Sclerosis |
| rs249677 | 5_141539339_C_A_b37 | NDFIP1 | ENSG00000131507 | 0.306305 | 0.00113988 | Multiple Sclerosis |
| rs140522 | 22_50971266_T_C_b37 | ODF3B | ENSG00000177989 | 0.324894 | 0.00179623 | Multiple Sclerosis |
| rs3923387 | 8_144986793_C_T_b37 | PLEC | ENSG00000178209 | -0.36765 | 0.0000194 | Multiple Sclerosis |
| rs12588969 | 14_103230758_G_C_b37 | RCOR1 | ENSG00000089902 | 0.20758 | 0.0176386 | Multiple Sclerosis |
| rs6032662 | 20_44734310_C_T_b37 | RPL13P2 | ENSG00000213820 | -0.312212 | 0.0331793 | Multiple Sclerosis |
| rs11083862 | 19_47638539_A_T_b37 | SAE1 | ENSG00000142230 | -0.152572 | 0.000165411 | Multiple Sclerosis |
| rs6427540 | 1_160634588_C_T_b37 | SETP9 | ENSG00000235101 | -0.809118 | 0.000000057 | Multiple Sclerosis |
| rs35540610 | 2_231121829_T_C_b37 | SP140 | ENSG00000079263 | -0.596225 | 3.7E-09 | Multiple Sclerosis |
| rs11161550 | 1_85682020_G_A_b37 | SYDE2 | ENSG00000097096 | 0.296308 | 0.0188359 | Multiple Sclerosis |
| rs3809627 | 16_30103160_C_A_b37 | TBX6 | ENSG00000149922 | -0.441233 | 2.35E-09 | Multiple Sclerosis |
| rs32658 | 5_118703662_G_T_b37 | TNFAIP8 | ENSG00000145779 | 0.311233 | 0.000535422 | Multiple Sclerosis |
| rs1800693 | 12_6440009_T_C_b37 | TNFRSF1A | ENSG00000067182 | 0.310718 | 0.00183555 | Multiple Sclerosis |
| rs2150879 | 17_57859210_G_A_b37 | VMP1 | ENSG00000062716 | 0.237067 | 0.0134751 | Multiple Sclerosis |
| rs354033 | 7_149289464_G_A_b37 | ZNF767P | ENSG00000133624 | -0.28839 | 0.00160115 | Multiple Sclerosis |
| rs4796224 | 17_34842521_A_G_b37 | ZNHIT3 | ENSG00000108278 | 0.536395 | 1.05E-20 | Multiple Sclerosis |
| rs4780401 | 16_11839326_G_T_b37 | AC007613.1 | ENSG00000262420 | -0.24778 | 0.0153847 | Rheumatoid Arthritis |
| rs7752903 | 6_138227364_T_G_b37 | AL591468.1 | ENSG00000226004 | 0.75994 | 0.029405 | Rheumatoid Arthritis |
| rs2736337 | 8_11341880_T_C_b37 | BLK | ENSG00000136573 | -0.461967 | 0.00000332 | Rheumatoid Arthritis |
| rs4239702 | 20_44749251_T_C_b37 | CD40 | ENSG00000101017 | 0.0985446 | 0.0472455 | Rheumatoid Arthritis |
| rs2234067 | 6_36355654_A_C_b37 | ETV7 | ENSG00000010030 | 0.312148 | 0.0393873 | Rheumatoid Arthritis |
| rs968567 | 11_61595564_C_T_b37 | FADS2 | ENSG00000134824 | 0.651641 | 0.0000205 | Rheumatoid Arthritis |
| rs2736337 | 8_11341880_T_C_b37 | FAM167A | ENSG00000154319 | 0.969226 | 2.13E-15 | Rheumatoid Arthritis |
| rs3778753 | 7_128580042_A_G_b37 | IRF5 | ENSG00000128604 | 0.179664 | 0.0240959 | Rheumatoid Arthritis |
| rs72634030 | 17_5272580_C_A_b37 | NUP88 | ENSG00000108559 | -0.399099 | 0.00950071 | Rheumatoid Arthritis |
| rs8032939 | 15_38834033_T_C_b37 | RASGRP1 | ENSG00000172575 | 0.309251 | 0.00442948 | Rheumatoid Arthritis |
| rs909685 | 22_39747671_T_A_b37 | SYNGR1 | ENSG00000100321 | 0.908838 | 3.74E-14 | Rheumatoid Arthritis |
| rs998731 | 8_81095395_C_T_b37 | TPD52 | ENSG00000076554 | 0.132647 | 0.0246293 | Rheumatoid Arthritis |
| rs3768792 | 2_213871709_G_A_b37 | AC093865.1 | ENSG00000273118 | -0.389706 | 0.00185752 | SLE |
| rs6932056 | 6_138242437_T_C_b37 | AL591468.1 | ENSG00000226004 | 0.700631 | 0.0407892 | SLE |
| rs4948496 | 10_63805617_T_C_b37 | ARID5B | ENSG00000150347 | -0.23305 | 0.0173212 | SLE |
| rs2736340 | 8_11343973_C_T_b37 | BLK | ENSG00000136573 | -0.519331 | 0.000000252 | SLE |
| rs2736340 | 8_11343973_C_T_b37 | FAM167A | ENSG00000154319 | 0.981001 | 7.5E-15 | SLE |
| rs849142 | 7_28185891_T_C_b37 | JAZF1 | ENSG00000153814 | -0.330514 | 0.00321638 | SLE |

| | | | | | | |
|---|---|---|---|---|---|---|
| rs2431697 | 5_159879978_T_C_b37 | MIR3142HG | ENSG00000253522 | 0.154932 | 0.0451329 | SLE |
| rs3794060 | 11_71187679_C_T_b37 | NADSYN1 | ENSG00000172890 | 0.541679 | 3.09E-08 | SLE |
| rs9462027 | 6_34797241_G_A_b37 | UHRF1BP1 | ENSG00000065060 | 0.213996 | 0.00101631 | SLE |
| rs6691977 | 1_200814959_T_C_b37 | CAMSAP2 | ENSG00000118200 | -0.25362 | 0.0161839 | Type 1 Diabetes |
| rs1615504 | 18_67526644_T_C_b37 | CD226 | ENSG00000150637 | 0.331861 | 0.00165379 | Type 1 Diabetes |
| rs34593439 | 15_79234957_G_A_b37 | CTSH | ENSG00000103811 | -0.61738 | 0.00000172 | Type 1 Diabetes |
| rs12453507 | 17_38053207_C_G_b37 | GSDMB | ENSG00000073605 | -0.446195 | 5.14E-09 | Type 1 Diabetes |
| rs402072 | 19_47219122_T_C_b37 | PRKD2 | ENSG00000105287 | 0.42004 | 0.000211687 | Type 1 Diabetes |
| rs72727394 | 15_38847022_C_T_b37 | RASGRP1 | ENSG00000172575 | 0.570013 | 0.000000809 | Type 1 Diabetes |
| rs193778 | 16_11351211_A_G_b37 | RMI2 | ENSG00000175643 | 0.348201 | 0.000445286 | Type 1 Diabetes |
| rs11203202 | 21_43825357_C_G_b37 | UBASH3A | ENSG00000160185 | 0.247024 | 0.0320288 | Type 1 Diabetes |
| rs12453507 | 17_38053207_C_G_b37 | ZPBP2 | ENSG00000186075 | 0.30694 | 0.00211491 | Type 1 Diabetes |
| rs10797432 | 1_2501338_C_T_b37 | AL139246.2 | ENSG00000225931 | 0.259515 | 0.0000893 | UlceRheumatoid Arthritis tive Colitis |
| rs798502 | 7_2789880_A_C_b37 | AMZ1 | ENSG00000174945 | 0.443672 | 0.000804977 | UlceRheumatoid Arthritis tive Colitis |
| rs7282490 | 21_45615741_G_A_b37 | AP001057.1 | ENSG00000232124 | 0.215473 | 0.031109 | UlceRheumatoid Arthritis tive Colitis |
| rs2188962 | 5_131770805_C_T_b37 | C5orf56 | ENSG00000197536 | 0.20139 | 0.0121974 | UlceRheumatoid Arthritis tive Colitis |
| rs10781499 | 9_139266405_G_A_b37 | CARD9 | ENSG00000187796 | -0.316039 | 0.00367951 | UlceRheumatoid Arthritis tive Colitis |
| rs727088 | 18_67530439_G_A_b37 | CD226 | ENSG00000150637 | 0.331287 | 0.00168229 | UlceRheumatoid Arthritis tive Colitis |
| rs7240004 | 18_46395022_A_G_b37 | CTIF | ENSG00000134030 | -0.24535 | 0.0228475 | UlceRheumatoid Arthritis tive Colitis |
| rs11010067 | 10_35295431_C_G_b37 | CUL2 | ENSG00000108094 | -0.210407 | 0.00323466 | UlceRheumatoid Arthritis tive Colitis |
| rs2155219 | 11_76299194_G_T_b37 | EMSY | ENSG00000158636 | -0.128727 | 0.0442355 | UlceRheumatoid Arthritis tive Colitis |
| rs798502 | 7_2789880_A_C_b37 | GNA12 | ENSG00000146535 | 0.894658 | 1.41E-16 | UlceRheumatoid Arthritis tive Colitis |
| rs12103 | 1_1247494_T_C_b37 | INTS11 | ENSG00000127054 | -0.326775 | 0.000601023 | UlceRheumatoid Arthritis tive Colitis |
| rs4728142 | 7_128573967_G_A_b37 | IRF5 | ENSG00000128604 | 0.179553 | 0.0266785 | UlceRheumatoid Arthritis tive Colitis |
| rs35675666 | 1_8021973_G_T_b37 | PARK7 | ENSG00000116288 | 0.163399 | 0.032923 | UlceRheumatoid Arthritis tive Colitis |
| rs1126510 | 19_47123783_A_G_b37 | PTGIR | ENSG00000160013 | 0.242418 | 0.00141346 | UlceRheumatoid Arthritis tive Colitis |
| rs2189234 | 4_106075498_T_G_b37 | TET2 | ENSG00000168769 | -0.13496 | 0.0305129 | UlceRheumatoid Arthritis tive Colitis |
| rs1728785 | 16_68591230_A_C_b37 | ZFP90 | ENSG00000184939 | 0.424271 | 0.000884164 | UlceRheumatoid Arthritis tive Colitis |
| rs10761659 | 10_64445564_A_G_b37 | ZNF365 | ENSG00000138311 | -0.348324 | 0.00617857 | UlceRheumatoid Arthritis tive Colitis |
| rs12946510 | 17_37912377_C_T_b37 | RP11-94L15.2 | ENSG00000264198 | 0.391955 | 0.000000449 | UlceRheumatoid Arthritis tive Colitis |

**Supplementary Table 2:** EDC genes which are the host genes expression correlated with EBV DNA copy number at a false discovery rate (FDR) of less than 5%. The correlation was tested in 433 LCL samples from GEUVADIS cohort.

**Supplementary Table 2:** EDC genes which are the host genes expression correlated with EBV DNA copy number at a false discovery rate (FDR) of less than 5%. The correlation was tested in 433 LCL samples from GEUVADIS cohort.

| Gene Ensembl ID | Gene Name | Rho | pvalue | FDR (Benjamini–Hochberg) |
|---|---|---|---|---|
| ENSG00000039068 | CDH1 | -0.355541733 | 2.39E-14 | 5.53E-10 |
| ENSG00000115604 | IL18R1 | -0.335932607 | 6.98E-13 | 8.07E-09 |
| ENSG00000109339 | MAPK10 | -0.323679751 | 5.11E-12 | 2.36E-08 |
| ENSG00000111962 | UST | -0.323969753 | 4.88E-12 | 2.82E-08 |
| ENSG00000135547 | HEY2 | -0.320706948 | 8.17E-12 | 3.15E-08 |
| ENSG00000183347 | GBP6 | -0.324849255 | 4.24E-12 | 3.27E-08 |
| ENSG00000171786 | NHLH1 | 0.316984881 | 1.46E-11 | 4.82E-08 |
| ENSG00000114541 | FRMD4B | -0.311913036 | 3.18E-11 | 9.19E-08 |
| ENSG00000115590 | IL1R2 | -0.307194693 | 6.47E-11 | 1.66E-07 |
| ENSG00000254716 | #N/A | 0.303438923 | 1.13E-10 | 2.01E-07 |
| ENSG00000143507 | DUSP10 | 0.303601458 | 1.10E-10 | 2.12E-07 |
| ENSG00000148468 | FAM171A1 | -0.30374564 | 1.08E-10 | 2.27E-07 |
| ENSG00000184292 | TACSTD2 | -0.303918511 | 1.05E-10 | 2.43E-07 |
| ENSG00000214357 | NEURL1B | 0.300493183 | 1.74E-10 | 2.68E-07 |
| ENSG00000151929 | BAG3 | -0.300713945 | 1.68E-10 | 2.78E-07 |
| ENSG00000091129 | NRCAM | -0.299042004 | 2.15E-10 | 2.92E-07 |
| ENSG00000241233 | KRTAP5-8 | -0.29925545 | 2.08E-10 | 3.01E-07 |
| ENSG00000154229 | PRKCA | -0.296541706 | 3.08E-10 | 3.95E-07 |
| ENSG00000185950 | IRS2 | 0.296092273 | 3.28E-10 | 3.99E-07 |
| ENSG00000146592 | CREB5 | -0.292792218 | 5.24E-10 | 6.06E-07 |
| ENSG00000152804 | HHEX | -0.292115445 | 5.77E-10 | 6.35E-07 |
| ENSG00000163629 | PTPN13 | -0.290814373 | 6.93E-10 | 6.96E-07 |
| ENSG00000089012 | SIRPG | -0.290962706 | 6.78E-10 | 7.13E-07 |
| ENSG00000166391 | MOGAT2 | 0.28986998 | 7.90E-10 | 7.62E-07 |
| ENSG00000163818 | LZTFL1 | -0.287459701 | 1.11E-09 | 1.02E-06 |
| ENSG00000111110 | PPM1H | -0.28653445 | 1.26E-09 | 1.12E-06 |
| ENSG00000162434 | JAK1 | -0.285605208 | 1.43E-09 | 1.22E-06 |
| ENSG00000010818 | HIVEP2 | -0.284944547 | 1.56E-09 | 1.29E-06 |
| ENSG00000262979 | CTD-2047H16.2 | 0.282718964 | 2.12E-09 | 1.69E-06 |
| ENSG00000189337 | KAZN | -0.280149044 | 3.00E-09 | 2.31E-06 |
| ENSG00000163545 | NUAK2 | -0.279612695 | 3.22E-09 | 2.40E-06 |
| ENSG00000134762 | DSC3 | -0.27808724 | 3.95E-09 | 2.85E-06 |
| ENSG00000112964 | GHR | -0.276388692 | 4.95E-09 | 3.36E-06 |
| ENSG00000135074 | ADAM19 | -0.276445601 | 4.91E-09 | 3.44E-06 |
| ENSG00000169508 | GPR183 | -0.275434765 | 5.61E-09 | 3.60E-06 |
| ENSG00000125378 | BMP4 | -0.275448068 | 5.60E-09 | 3.70E-06 |
| ENSG00000198825 | INPP5F | -0.273922169 | 6.85E-09 | 4.17E-06 |
| ENSG00000168447 | SCNN1B | 0.273965331 | 6.81E-09 | 4.25E-06 |
| ENSG00000102096 | PIM2 | -0.272905495 | 7.82E-09 | 4.64E-06 |
| ENSG00000168126 | OR2W6P | 0.272648443 | 8.09E-09 | 4.67E-06 |
| ENSG00000068831 | RASGRP2 | -0.271829915 | 9.00E-09 | 5.07E-06 |
| ENSG00000260174 | CTD-2022H16.3 | 0.271151664 | 9.82E-09 | 5.41E-06 |
| ENSG00000115009 | CCL20 | -0.269461172 | 1.22E-08 | 6.57E-06 |
| ENSG00000198719 | DLL1 | -0.269143591 | 1.27E-08 | 6.69E-06 |
| ENSG00000138755 | CXCL9 | -0.2685289 | 1.38E-08 | 6.92E-06 |
| ENSG00000144476 | ACKR3 | -0.26864937 | 1.36E-08 | 6.97E-06 |
| ENSG00000165775 | FUNDC2 | -0.267879621 | 1.50E-08 | 7.36E-06 |
| ENSG00000166828 | SCNN1G | 0.267236402 | 1.63E-08 | 7.83E-06 |
| ENSG00000124657 | OR2B6 | 0.266555046 | 1.77E-08 | 8.36E-06 |
| ENSG00000166105 | GLB1L3 | -0.266087654 | 1.88E-08 | 8.70E-06 |
| ENSG00000122877 | EGR2 | 0.26535508 | 2.06E-08 | 9.18E-06 |
| ENSG00000230847 | RP11-195E2.1 | -0.265479615 | 2.03E-08 | 9.21E-06 |
| ENSG00000039560 | RAI14 | -0.264202563 | 2.39E-08 | 1.04E-05 |
| ENSG00000249959 | RP11-252I13.2 | -0.262724039 | 2.87E-08 | 1.23E-05 |
| ENSG00000143028 | SYPL2 | -0.261800044 | 3.23E-08 | 1.26E-05 |
| ENSG00000172548 | NIPAL4 | -0.261468642 | 3.36E-08 | 1.27E-05 |
| ENSG00000012124 | CD22 | -0.262126051 | 3.10E-08 | 1.28E-05 |
| ENSG00000174099 | MSRB3 | -0.261802779 | 3.23E-08 | 1.29E-05 |
| ENSG00000106689 | LHX2 | -0.261483202 | 3.36E-08 | 1.29E-05 |
| ENSG00000138722 | MMRN1 | -0.26214985 | 3.09E-08 | 1.30E-05 |
| ENSG00000182732 | RGS6 | -0.261839437 | 3.21E-08 | 1.30E-05 |
| ENSG00000126262 | FFAR2 | -0.260974125 | 3.58E-08 | 1.33E-05 |
| ENSG00000218336 | TENM3 | -0.260358548 | 3.86E-08 | 1.42E-05 |
| ENSG00000151632 | AKR1C2 | -0.259535511 | 4.27E-08 | 1.52E-05 |
| ENSG00000206579 | XKR4 | -0.259634622 | 4.22E-08 | 1.53E-05 |
| ENSG00000141506 | PIK3R5 | -0.258614177 | 4.79E-08 | 1.68E-05 |
| ENSG00000064655 | EYA2 | -0.258041762 | 5.14E-08 | 1.77E-05 |
| ENSG00000162654 | GBP4 | -0.257793876 | 5.30E-08 | 1.80E-05 |
| ENSG00000007312 | CD79B | -0.257548945 | 5.46E-08 | 1.83E-05 |
| ENSG00000183785 | TUBA8 | -0.257323083 | 5.61E-08 | 1.85E-05 |
| ENSG00000205755 | CRLF2 | -0.257174085 | 5.71E-08 | 1.86E-05 |
| ENSG00000115616 | SLC9A2 | -0.257036469 | 5.81E-08 | 1.87E-05 |
| ENSG00000134215 | VAV3 | -0.256785996 | 5.99E-08 | 1.87E-05 |
| ENSG00000099284 | H2AFY2 | -0.256824871 | 5.96E-08 | 1.89E-05 |
| ENSG00000145113 | MUC4 | 0.256489478 | 6.21E-08 | 1.92E-05 |
| ENSG00000134755 | DSC2 | -0.256357331 | 6.31E-08 | 1.92E-05 |
| ENSG00000090013 | BLVRB | 0.255516039 | 6.99E-08 | 2.10E-05 |
| ENSG00000260628 | RP11-1166P10.1 | 0.255374284 | 7.12E-08 | 2.11E-05 |
| ENSG00000108639 | SYNGR2 | 0.254242092 | 8.16E-08 | 2.39E-05 |
| ENSG00000138131 | LOXL4 | 0.253646691 | 8.77E-08 | 2.53E-05 |
| ENSG00000197622 | CDC42SE1 | -0.252883972 | 9.61E-08 | 2.74E-05 |
| ENSG00000035664 | DAPK2 | -0.252330394 | 1.03E-07 | 2.90E-05 |
| ENSG00000127152 | BCL11B | -0.252007565 | 1.07E-07 | 2.97E-05 |
| ENSG00000225485 | ARHGAP23 | 0.25126531 | 1.17E-07 | 3.21E-05 |
| ENSG00000095397 | DFNB31 | -0.25099089 | 1.21E-07 | 3.28E-05 |
| ENSG00000134028 | ADAMDEC1 | -0.250201038 | 1.32E-07 | 3.56E-05 |
| ENSG00000260781 | ARHGAP23P1 | 0.249261967 | 1.48E-07 | 3.93E-05 |
| ENSG00000058085 | LAMC2 | -0.248812755 | 1.56E-07 | 4.06E-05 |
| ENSG00000176165 | FOXG1 | -0.248815564 | 1.56E-07 | 4.10E-05 |
| ENSG00000126759 | CFP | -0.248494361 | 1.62E-07 | 4.12E-05 |
| ENSG00000255693 | RP11-766N7.3 | -0.248507664 | 1.62E-07 | 4.16E-05 |
| ENSG00000132669 | RIN2 | -0.248203238 | 1.68E-07 | 4.22E-05 |
| ENSG00000129422 | MTUS1 | -0.247950548 | 1.73E-07 | 4.30E-05 |
| ENSGR0000205755 | #N/A | -0.247165943 | 1.89E-07 | 4.66E-05 |
| ENSG00000006042 | TMEM98 | 0.24703424 | 1.92E-07 | 4.68E-05 |
| ENSG00000139687 | RB1 | 0.246923009 | 1.95E-07 | 4.70E-05 |
| ENSG00000152465 | NMT2 | -0.246489687 | 2.05E-07 | 4.84E-05 |
| ENSG00000255129 | RP11-839D17.3 | -0.246565517 | 2.03E-07 | 4.85E-05 |
| ENSG00000135925 | WNT10A | 0.246089699 | 2.15E-07 | 5.02E-05 |
| ENSG00000250626 | RP11-756P10.2 | 0.245952969 | 2.18E-07 | 5.05E-05 |
| ENSG00000251160 | #N/A | -0.245266588 | 2.37E-07 | 5.42E-05 |
| ENSG00000155858 | LSM11 | -0.244667936 | 2.54E-07 | 5.75E-05 |
| ENSG00000103653 | CSK | -0.244551679 | 2.57E-07 | 5.77E-05 |
| ENSG00000006075 | CCL3 | 0.243825905 | 2.80E-07 | 6.21E-05 |
| ENSG00000100154 | TTC28 | -0.24346967 | 2.91E-07 | 6.24E-05 |
| ENSG00000101307 | SIRPB1 | -0.243536039 | 2.89E-07 | 6.25E-05 |
| ENSG00000154217 | PITPNC1 | -0.243573953 | 2.88E-07 | 6.28E-05 |
| ENSG00000137573 | SULF1 | -0.2435877 | 2.87E-07 | 6.33E-05 |
| ENSG00000168263 | KCNV2 | 0.242078652 | 3.42E-07 | 7.18E-05 |
| ENSG00000181264 | TMEM136 | -0.241990258 | 3.45E-07 | 7.19E-05 |
| ENSG00000111249 | CUX2 | -0.242094542 | 3.41E-07 | 7.24E-05 |
| ENSG00000143013 | LMO4 | 0.241716135 | 3.56E-07 | 7.36E-05 |
| ENSG00000260186 | RP11-481J2.2 | -0.241016524 | 3.86E-07 | 7.90E-05 |
| ENSG00000197822 | OCLN | -0.240258452 | 4.21E-07 | 8.53E-05 |
| ENSG00000133069 | TMCC2 | -0.239389224 | 4.64E-07 | 9.34E-05 |
| ENSG00000002726 | AOC1 | 0.238961224 | 4.88E-07 | 9.72E-05 |
| ENSG00000185686 | PRAME | -0.238849919 | 4.94E-07 | 9.76E-05 |
| ENSG00000169291 | SHE | -0.238098721 | 5.37E-07 | 0.000105302 |
| ENSG00000099974 | DDTL | -0.237695554 | 5.62E-07 | 0.000105713 |
| ENSG00000153395 | LPCAT1 | -0.237703905 | 5.62E-07 | 0.000106479 |
| ENSG00000198010 | DLGAP2 | 0.237716544 | 5.61E-07 | 0.000107206 |
| ENSG00000125458 | NT5C | -0.237728443 | 5.60E-07 | 0.000107955 |
| ENSG00000115112 | TFCP2L1 | 0.237760371 | 5.58E-07 | 0.000108472 |
| ENSG00000155189 | AGPAT5 | -0.237271028 | 5.90E-07 | 0.000109985 |
| ENSG00000112137 | PHACTR1 | 0.236639634 | 6.33E-07 | 0.000117109 |
| ENSG00000129353 | SLC44A2 | -0.236277782 | 6.59E-07 | 0.00012098 |
| ENSG00000125810 | CD93 | -0.236037508 | 6.77E-07 | 0.00012233 |

| | | | | |
|---|---|---|---|---|
| ENSG00000013016 | EHD3 | -0.236091608 | 6.73E-07 | 0.00012255 |
| ENSG00000177469 | PTRF | 0.235610617 | 7.10E-07 | 0.000122552 |
| ENSG00000101204 | CHRNA4 | 0.235660578 | 7.06E-07 | 0.000122788 |
| ENSG00000101161 | PRPF6 | -0.235699454 | 7.03E-07 | 0.000123183 |
| ENSG00000108960 | MMD | -0.235828644 | 6.93E-07 | 0.000123289 |
| ENSG00000136110 | LECT1 | -0.235722735 | 7.01E-07 | 0.000123802 |
| ENSG00000254470 | AP5B1 | 0.235845126 | 6.92E-07 | 0.000124016 |
| ENSG00000134070 | IRAK2 | -0.235389485 | 7.28E-07 | 0.000124679 |
| ENSG00000163347 | CLDN1 | -0.234456252 | 8.07E-07 | 0.000137285 |
| ENSG00000101916 | TLR8 | 0.234115759 | 8.39E-07 | 0.000138491 |
| ENSG00000157693 | C9orf91 | -0.234301193 | 8.21E-07 | 0.000138646 |
| ENSG00000117322 | CR2 | -0.234145544 | 8.36E-07 | 0.000139028 |
| ENSG00000131462 | TUBG1 | 0.234203709 | 8.30E-07 | 0.000139136 |
| ENSG00000007384 | RHBDF1 | 0.233883836 | 8.60E-07 | 0.000141083 |
| ENSG00000154928 | EPHB1 | 0.233749842 | 8.73E-07 | 0.000142181 |
| ENSG00000178996 | SNX18 | -0.233535509 | 8.94E-07 | 0.00014457 |
| ENSG00000081320 | STK17B | -0.232827334 | 9.67E-07 | 0.000155226 |
| ENSG00000203883 | SOX18 | -0.232637974 | 9.87E-07 | 0.000156323 |
| ENSG00000224298 | AC069363.1 | 0.232644108 | 9.86E-07 | 0.000157295 |
| ENSG00000107738 | C10orf54 | -0.232491784 | 1.00E-06 | 0.000157775 |
| ENSG00000173221 | GLRX | -0.232373753 | 1.02E-06 | 0.000158754 |
| ENSG00000197852 | FAM212B | 0.231754628 | 1.09E-06 | 0.000166538 |
| ENSG00000172264 | MACROD2 | 0.231855439 | 1.08E-06 | 0.000166919 |
| ENSG00000058668 | ATP2B4 | -0.231670226 | 1.10E-06 | 0.000166979 |
| ENSG00000115607 | IL18RAP | -0.231589149 | 1.11E-06 | 0.000167367 |
| ENSG00000182676 | PPP1R27 | 0.231760763 | 1.09E-06 | 0.000167534 |
| ENSG00000102024 | PLS3 | -0.23117194 | 1.16E-06 | 0.00017292 |
| ENSG00000050438 | SLC4A8 | -0.231192708 | 1.16E-06 | 0.000173648 |
| ENSG00000164741 | DLC1 | -0.231016734 | 1.18E-06 | 0.000174749 |
| ENSG00000173662 | TAS1R1 | 0.23078215 | 1.21E-06 | 0.00017814 |
| ENSG00000099625 | C19orf26 | 0.230427541 | 1.26E-06 | 0.000181687 |
| ENSG00000164091 | WDR82 | -0.230472329 | 1.25E-06 | 0.00018194 |
| ENSG00000174576 | NPAS4 | 0.230477651 | 1.25E-06 | 0.000182986 |
| ENSG00000026025 | VIM | 0.229453881 | 1.40E-06 | 0.00020071 |
| ENSG00000115353 | TACR1 | -0.229372139 | 1.41E-06 | 0.000201247 |
| ENSG00000221890 | NPTXR | 0.228626262 | 1.53E-06 | 0.000216823 |
| ENSG00000184414 | RP11-44M6.3 | 0.228431737 | 1.56E-06 | 0.000220076 |
| ENSG00000255883 | AC099522.2 | -0.228352803 | 1.57E-06 | 0.000220613 |
| ENSG00000177721 | ANXA2R | -0.228049116 | 1.63E-06 | 0.000226583 |
| ENSG00000180573 | HIST1H2AC | 0.227910613 | 1.65E-06 | 0.00022861 |
| ENSG00000178248 | AP000345.1 | 0.227674404 | 1.69E-06 | 0.0002331 |
| ENSG00000167081 | PBX3 | 0.227604487 | 1.71E-06 | 0.000233471 |
| ENSG00000103313 | MEFV | 0.227481283 | 1.73E-06 | 0.000233816 |
| ENSG00000147255 | IGSF1 | 0.227519346 | 1.72E-06 | 0.000234231 |
| ENSG00000234438 | KBTBD13 | 0.226508805 | 1.92E-06 | 0.000258004 |
| ENSG00000234961 | RP11-124N14.3 | 0.226012736 | 2.02E-06 | 0.000270478 |
| ENSG00000157343 | ARMC12 | -0.225895741 | 2.05E-06 | 0.000272303 |
| ENSG00000037042 | TUBG2 | 0.225694859 | 2.09E-06 | 0.000276609 |
| ENSG00000166173 | LARP6 | -0.225314382 | 2.18E-06 | 0.000286411 |
| ENSG00000124422 | USP22 | -0.225121853 | 2.23E-06 | 0.000290684 |
| ENSG00000239445 | ST3GAL6-AS1 | -0.225050014 | 2.24E-06 | 0.000291267 |
| ENSG00000220884 | MESTP1 | -0.224931836 | 2.27E-06 | 0.000293299 |
| ENSG00000168685 | IL7R | -0.224854898 | 2.29E-06 | 0.000294063 |
| ENSG00000103710 | RASL12 | 0.22445587 | 2.39E-06 | 0.000305084 |
| ENSG00000163346 | PBXIP1 | -0.224395709 | 2.40E-06 | 0.000305348 |
| ENSG00000133454 | MYO18B | -0.223884194 | 2.54E-06 | 0.000320572 |
| ENSG00000164330 | EBF1 | -0.223628842 | 2.61E-06 | 0.000327548 |
| ENSG00000128510 | CPA4 | -0.223501647 | 2.64E-06 | 0.000330181 |
| ENSG00000062524 | LTK | -0.222829678 | 2.84E-06 | 0.0003525 |
| ENSG00000243978 | RGAG1 | 0.221897849 | 3.13E-06 | 0.000386644 |
| ENSG00000153064 | BANK1 | -0.221620843 | 3.22E-06 | 0.000395903 |
| ENSG00000225327 | USP17L3 | 0.221542944 | 3.25E-06 | 0.000397029 |
| ENSG00000111490 | TBC1D30 | -0.221381456 | 3.30E-06 | 0.000401663 |
| ENSG00000230549 | USP17L1P | 0.221147538 | 3.38E-06 | 0.000409442 |
| ENSG00000155130 | MARCKS | -0.220753758 | 3.52E-06 | 0.00042001 |
| ENSG00000168824 | NSG1 | 0.220843629 | 3.49E-06 | 0.000420428 |
| ENSG00000175556 | LONRF3 | 0.220781251 | 3.51E-06 | 0.000420977 |
| ENSG00000174827 | PDZK1 | 0.220662112 | 3.56E-06 | 0.000421864 |
| ENSG00000261239 | ANKRD26P1 | 0.220397522 | 3.66E-06 | 0.000431431 |
| ENSG00000225889 | AC074289.1 | -0.220257911 | 3.71E-06 | 0.000435518 |
| ENSG00000261889 | RP11-473M20.16 | 0.220034857 | 3.80E-06 | 0.000443478 |
| ENSG00000167613 | LAIR1 | -0.219855557 | 3.87E-06 | 0.000449538 |
| ENSG00000183780 | SLC35F3 | -0.2195025 | 4.01E-06 | 0.000463966 |
| ENSG00000140280 | LYSMD2 | -0.219183218 | 4.15E-06 | 0.00047717 |
| ENSG00000162878 | PKDCC | -0.218929789 | 4.26E-06 | 0.000487412 |
| ENSG00000114520 | SNX4 | 0.21875714 | 4.33E-06 | 0.000491315 |
| ENSG00000169862 | CTNND2 | -0.21867732 | 4.37E-06 | 0.000492963 |
| ENSG00000102678 | FGF9 | 0.218766157 | 4.33E-06 | 0.000493277 |
| ENSG00000122176 | FMOD | -0.218596317 | 4.41E-06 | 0.000494687 |
| ENSG00000248603 | #N/A | 0.218342592 | 4.52E-06 | 0.000505344 |
| ENSG00000229671 | LINC01150 | -0.218086723 | 4.64E-06 | 0.000516341 |
| ENSG00000134247 | PTGFRN | -0.217774906 | 4.80E-06 | 0.000530614 |
| ENSG00000102531 | FNDC3A | 0.21682557 | 5.29E-06 | 0.000582074 |
| ENSG00000203697 | CAPN8 | -0.216650622 | 5.38E-06 | 0.000589772 |
| ENSG00000149212 | SESN3 | -0.216564815 | 5.43E-06 | 0.000592161 |
| ENSG00000171700 | RGS19 | -0.216274949 | 5.59E-06 | 0.000607085 |
| ENSG00000225493 | LINC01107 | 0.216188699 | 5.64E-06 | 0.000609589 |
| ENSG00000177519 | RPRM | -0.215583468 | 6.00E-06 | 0.000642344 |
| ENSG00000076928 | ARHGEF1 | -0.215599876 | 5.99E-06 | 0.000644256 |
| ENSG00000163479 | SSR2 | -0.215446074 | 6.09E-06 | 0.000648378 |
| ENSG00000147394 | ZNF185 | -0.215392343 | 6.12E-06 | 0.000648938 |
| ENSG00000196372 | ASB13 | -0.215303653 | 6.17E-06 | 0.000651822 |
| ENSG00000224559 | LINC01087 | -0.214961756 | 6.39E-06 | 0.000671769 |
| ENSG00000006210 | CX3CL1 | -0.21467056 | 6.58E-06 | 0.000688756 |
| ENSG00000130755 | GMFG | -0.214588596 | 6.64E-06 | 0.000691368 |
| ENSG00000144218 | AFF3 | -0.214496064 | 6.70E-06 | 0.000694744 |
| ENSG00000128849 | CGNL1 | -0.213842801 | 7.16E-06 | 0.000738828 |
| ENSG00000087237 | CETP | -0.213782337 | 7.20E-06 | 0.000740044 |
| ENSG00000173801 | JUP | -0.213650485 | 7.30E-06 | 0.000746628 |
| ENSG00000113739 | STC2 | 0.213353007 | 7.52E-06 | 0.000762586 |
| ENSG00000215193 | PEX26 | -0.213263578 | 7.59E-06 | 0.000762788 |
| ENSG00000196914 | ARHGEF12 | -0.213387521 | 7.49E-06 | 0.00076329 |
| ENSG00000148154 | UGCG | 0.21328952 | 7.57E-06 | 0.000764122 |
| ENSG00000064309 | CDON | -0.212934024 | 7.84E-06 | 0.000785066 |
| ENSG00000198223 | CSF2RA | 0.21264231 | 8.08E-06 | 0.000804907 |
| ENSG00000130787 | HIP1R | -0.212031093 | 8.59E-06 | 0.000852047 |
| ENSG00000227053 | RP11-395B7.4 | -0.211586981 | 8.98E-06 | 0.000886894 |
| ENSG00000249960 | RP11-791G16.5 | 0.211378044 | 9.16E-06 | 0.000897897 |
| ENSG00000172403 | SYNPO2 | -0.21140369 | 9.14E-06 | 0.000899415 |
| ENSG00000058056 | USP13 | -0.211292385 | 9.24E-06 | 0.000901776 |
| ENSG00000096060 | FKBP5 | -0.211028017 | 9.49E-06 | 0.000914261 |
| ENSG00000119943 | PYROXD2 | -0.210981012 | 9.53E-06 | 0.000914736 |
| ENSG00000159166 | LAD1 | 0.211058319 | 9.46E-06 | 0.000915322 |
| ENSG00000142920 | ADC | -0.210856995 | 9.65E-06 | 0.000918469 |
| ENSG00000104371 | DKK4 | 0.21106438 | 9.46E-06 | 0.000918613 |
| ENSG00000136002 | ARHGEF4 | -0.210886705 | 9.62E-06 | 0.000919543 |
| ENSG00000070601 | FRMPD1 | -0.210699202 | 9.81E-06 | 0.000925374 |
| ENSG00000148948 | LRRC4C | 0.210705927 | 9.80E-06 | 0.000928545 |
| ENSG00000157978 | LDLRAP1 | 0.210612064 | 9.89E-06 | 0.000929626 |
| ENSG00000113532 | ST8SIA4 | -0.210555303 | 9.95E-06 | 0.000931096 |
| ENSG00000102038 | SMARCA1 | -0.210177191 | 1.03E-05 | 0.000958922 |
| ENSG00000133561 | GIMAP6 | -0.210119174 | 1.04E-05 | 0.000960599 |
| ENSG00000106484 | MEST | -0.210078229 | 1.04E-05 | 0.000960659 |
| ENSG00000197746 | PSAP | -0.210196925 | 1.03E-05 | 0.000960906 |
| ENSG00000178015 | GPR150 | -0.209759095 | 1.08E-05 | 0.000987557 |
| ENSG00000105220 | GPI | 0.209695534 | 1.08E-05 | 0.000989859 |
| ENSG00000127863 | TNFRSF19 | -0.209629683 | 1.09E-05 | 0.000992407 |
| ENSG00000260432 | RP11-297M9.2 | -0.209573661 | 1.10E-05 | 0.000994001 |
| ENSG00000150594 | ADRA2A | -0.209493766 | 1.10E-05 | 0.000994084 |
| ENSG00000113389 | NPR3 | -0.209515274 | 1.10E-05 | 0.000995854 |
| ENSG00000137090 | DMRT1 | -0.209132136 | 1.15E-05 | 0.001026222 |
| ENSG00000162396 | PARS2 | 0.208951875 | 1.17E-05 | 0.001040588 |

| | | | | |
|---|---|---|---|---|
| ENSG00000141639 | MAPK4 | 0.208426391 | 1.23E-05 | 0.001091607 |
| ENSG00000161249 | DMKN | -0.208310282 | 1.24E-05 | 0.0010957 |
| ENSG00000163637 | PRICKLE2 | -0.208331124 | 1.24E-05 | 0.001097648 |
| ENSG00000123329 | ARHGAP9 | -0.207939413 | 1.29E-05 | 0.001132002 |
| ENSG00000166394 | CYB5R2 | -0.207850502 | 1.30E-05 | 0.001133292 |
| ENSG00000186009 | ATP4B | 0.207879326 | 1.30E-05 | 0.001134379 |
| ENSG00000133059 | DSTYK | -0.20758266 | 1.33E-05 | 0.001159047 |
| ENSG00000119698 | PPP4R4 | -0.207479042 | 1.35E-05 | 0.001166483 |
| ENSG00000081913 | PHLPP1 | -0.207375127 | 1.36E-05 | 0.001174002 |
| ENSG00000074219 | TEAD2 | -0.207302328 | 1.37E-05 | 0.001177993 |
| ENSG00000182575 | NXPH3 | -0.207187254 | 1.39E-05 | 0.001182524 |
| ENSG00000105989 | WNT2 | -0.207204918 | 1.38E-05 | 0.001184857 |
| ENSG00000255375 | RP1-65P5.5 | -0.20706405 | 1.40E-05 | 0.001188073 |
| ENSG00000162989 | KCNJ3 | 0.207093687 | 1.40E-05 | 0.001188998 |
| ENSG00000188373 | C10orf99 | 0.206781279 | 1.44E-05 | 0.001216859 |
| ENSG00000249193 | HSPD1P5 | 0.206679508 | 1.46E-05 | 0.001224516 |
| ENSG00000019582 | CD74 | 0.206231036 | 1.52E-05 | 0.001274533 |
| ENSGR0000198223 | #N/A | 0.205981301 | 1.56E-05 | 0.001301143 |
| ENSG00000182814 | FUNDC2P2 | -0.205712573 | 1.60E-05 | 0.001330721 |
| ENSG00000110777 | POU2AF1 | -0.205579834 | 1.62E-05 | 0.001343131 |
| ENSG00000116698 | SMG7 | -0.205310071 | 1.66E-05 | 0.001359214 |
| ENSG00000164509 | IL31RA | 0.205412212 | 1.65E-05 | 0.001360259 |
| ENSG00000255211 | RP11-1195F20.7 | 0.205372967 | 1.65E-05 | 0.001360577 |
| ENSG00000141524 | TMC6 | 0.205315252 | 1.66E-05 | 0.001363353 |
| ENSG00000250666 | RP11-463J17.1 | 0.205214509 | 1.68E-05 | 0.001367015 |
| ENSG00000121594 | CD80 | -0.205142678 | 1.69E-05 | 0.001371719 |
| ENSG00000198771 | RCSD1 | -0.205078592 | 1.70E-05 | 0.001375428 |
| ENSG00000187134 | AKR1C1 | -0.204372699 | 1.82E-05 | 0.001462202 |
| ENSG00000082512 | TRAF5 | -0.204408027 | 1.82E-05 | 0.001462309 |
| ENSG00000168386 | FILIP1L | -0.204294579 | 1.84E-05 | 0.001463096 |
| ENSG00000065923 | SLC9A7 | 0.204298496 | 1.83E-05 | 0.001467599 |
| ENSG00000233426 | EIF3FP3 | -0.20419436 | 1.85E-05 | 0.001472204 |
| ENSG00000110074 | FOXRED1 | 0.204034424 | 1.88E-05 | 0.001479792 |
| ENSG00000260339 | HEXA-AS1 | 0.204037823 | 1.88E-05 | 0.001484361 |
| ENSG00000150712 | MTMR12 | -0.204060439 | 1.88E-05 | 0.001486206 |
| ENSG00000135638 | EMX1 | 0.203928218 | 1.90E-05 | 0.001489919 |
| ENSG00000226430 | USP17L7 | 0.203808857 | 1.92E-05 | 0.001502024 |
| ENSG00000117595 | IRF6 | 0.20348019 | 1.98E-05 | 0.00154497 |
| ENSG00000138378 | STAT4 | -0.202804303 | 2.12E-05 | 0.001642795 |
| ENSG00000160886 | LY6K | 0.202733574 | 2.13E-05 | 0.001642911 |
| ENSG00000119138 | KLF9 | 0.202753381 | 2.13E-05 | 0.001645289 |
| ENSG00000125868 | DSTN | -0.202639275 | 2.15E-05 | 0.001646815 |
| ENSG00000002919 | SNX11 | -0.202671491 | 2.14E-05 | 0.001647201 |
| ENSG00000065491 | TBC1D22B | -0.202357753 | 2.21E-05 | 0.001680559 |
| ENSG00000088387 | DOCK9 | -0.202383029 | 2.20E-05 | 0.001682038 |
| ENSG00000174282 | ZBTB4 | 0.202233736 | 2.24E-05 | 0.001689447 |
| ENSG00000175329 | ISX | -0.202258569 | 2.23E-05 | 0.001690976 |
| ENSG00000167654 | ATCAY | 0.20184269 | 2.32E-05 | 0.001747873 |
| ENSG00000101439 | CST3 | 0.201734045 | 2.34E-05 | 0.001760305 |
| ENSG00000228594 | C1orf233 | -0.201636487 | 2.37E-05 | 0.001770966 |
| ENSG00000198125 | MB | 0.201413286 | 2.42E-05 | 0.001797301 |
| ENSG00000183137 | CEP57L1 | 0.201427107 | 2.41E-05 | 0.001800734 |
| ENSG00000101608 | MYL12A | -0.201261923 | 2.45E-05 | 0.001817478 |
| ENSG00000174010 | KLHL15 | 0.201139679 | 2.48E-05 | 0.001832806 |
| ENSG00000224660 | SH3BP5-AS1 | -0.200652849 | 2.60E-05 | 0.001913213 |
| ENSG00000144908 | ALDH1L1 | -0.200559652 | 2.62E-05 | 0.001924029 |
| ENSG00000137942 | FNBP1L | -0.200277694 | 2.69E-05 | 0.001969746 |
| ENSG00000152076 | CCDC74B | -0.200055675 | 2.75E-05 | 0.002005122 |
| ENSG00000203772 | SPRN | -0.200008965 | 2.76E-05 | 0.002007651 |
| ENSG00000255327 | RP11-555G19.1 | 0.199851098 | 2.80E-05 | 0.002025025 |
| ENSG00000181467 | RAP2B | 0.199870388 | 2.80E-05 | 0.002027676 |
| ENSG00000130590 | SAMD10 | -0.199735506 | 2.83E-05 | 0.002040823 |
| ENSG00000099977 | DDT | -0.199535734 | 2.89E-05 | 0.002073117 |
| ENSG00000183049 | CAMK1D | -0.19946442 | 2.91E-05 | 0.002074193 |
| ENSG00000235778 | AC130360.8 | 0.199465743 | 2.91E-05 | 0.002080357 |
| ENSG00000150625 | GPM6A | -0.199306768 | 2.95E-05 | 0.002085859 |
| ENSG00000100024 | UPB1 | -0.199332488 | 2.94E-05 | 0.002087207 |
| ENSG00000156052 | GNAQ | -0.199353625 | 2.94E-05 | 0.002089468 |
| ENSG00000230033 | RP5-1121A15.3 | 0.199137149 | 3.00E-05 | 0.002106484 |
| ENSG00000126756 | UXT | -0.199054816 | 3.02E-05 | 0.002110012 |
| ENSG00000116574 | RHOU | 0.199141362 | 3.00E-05 | 0.002112075 |
| ENSG00000101421 | CHMP4B | -0.199072628 | 3.02E-05 | 0.002112867 |
| ENSG00000249509 | RP11-402J6.1 | -0.198698507 | 3.12E-05 | 0.002175153 |
| ENSG00000251191 | LINC00589 | 0.198378634 | 3.22E-05 | 0.002234571 |
| ENSG00000111348 | ARHGDIB | -0.198304283 | 3.24E-05 | 0.002236733 |
| ENSG00000140955 | ADAD2 | 0.198313078 | 3.24E-05 | 0.002241581 |
| ENSG00000149294 | NCAM1 | -0.198186918 | 3.28E-05 | 0.002254684 |
| ENSG00000140263 | SORD | 0.19800067 | 3.33E-05 | 0.002287459 |
| ENSG00000184232 | OAF | -0.197803928 | 3.40E-05 | 0.002302531 |
| ENSG00000157657 | ZNF618 | 0.197814866 | 3.39E-05 | 0.002306943 |
| ENSG00000196263 | ZNF471 | -0.197872218 | 3.37E-05 | 0.002308212 |
| ENSG00000073008 | PVR | -0.197822405 | 3.39E-05 | 0.002312125 |
| ENSG00000223443 | USP17L2 | 0.197586639 | 3.47E-05 | 0.002335962 |
| ENSG00000114745 | GORASP1 | -0.19759706 | 3.46E-05 | 0.002340514 |
| ENSG00000172005 | MAL | -0.19750911 | 3.49E-05 | 0.002346056 |
| ENSG00000064225 | ST3GAL6 | -0.197418795 | 3.52E-05 | 0.002352202 |
| ENSG00000158748 | HTR6 | -0.197434389 | 3.51E-05 | 0.002355596 |
| ENSG00000142185 | TRPM2 | -0.197169504 | 3.60E-05 | 0.002400477 |
| ENSG00000105254 | TBCB | -0.196999146 | 3.66E-05 | 0.002417894 |
| ENSG00000164291 | ARSK | -0.197004985 | 3.66E-05 | 0.00242351 |
| ENSG00000120265 | PCMT1 | 0.197012006 | 3.66E-05 | 0.002428886 |
| ENSG00000232977 | LINC00327 | -0.196559765 | 3.81E-05 | 0.002511379 |
| ENSG00000154102 | C16orf74 | -0.196430279 | 3.86E-05 | 0.00253448 |
| ENSG00000086289 | EPDR1 | 0.196120236 | 3.97E-05 | 0.002593557 |
| ENSG00000178409 | BEND3 | -0.196142112 | 3.96E-05 | 0.002595651 |
| ENSG00000174567 | GOLT1A | 0.195827118 | 4.08E-05 | 0.002657297 |
| ENSG00000182511 | FES | -0.195708643 | 4.12E-05 | 0.002678989 |
| ENSG00000145335 | SNCA | -0.195665703 | 4.14E-05 | 0.002682094 |
| ENSG00000133740 | E2F5 | -0.195629488 | 4.15E-05 | 0.00268356 |
| ENSG00000260754 | #N/A | -0.195530895 | 4.19E-05 | 0.00270054 |
| ENSG00000177791 | MYOZ1 | -0.195454696 | 4.22E-05 | 0.002712031 |
| ENSG00000145569 | FAM105A | -0.19521664 | 4.32E-05 | 0.002749276 |
| ENSG00000182118 | FAM89A | -0.195269705 | 4.30E-05 | 0.002751031 |
| ENSG00000145850 | TIMD4 | -0.19523859 | 4.31E-05 | 0.002751307 |
| ENSG00000116667 | C1orf21 | -0.194511634 | 4.61E-05 | 0.002925327 |
| ENSG00000229255 | RP11-407H12.8 | -0.194079865 | 4.79E-05 | 0.003018561 |
| ENSG00000164484 | TMEM200A | -0.193984228 | 4.83E-05 | 0.003020414 |
| ENSG00000156136 | DCK | -0.194129974 | 4.77E-05 | 0.003021203 |
| ENSG00000171476 | HOPX | -0.194035003 | 4.81E-05 | 0.003022749 |
| ENSG00000173218 | VANGL1 | -0.194002631 | 4.83E-05 | 0.003023505 |
| ENSG00000171227 | TMEM37 | -0.194084299 | 4.79E-05 | 0.003025577 |
| ENSG00000248290 | TNXA | 0.193893691 | 4.87E-05 | 0.003037333 |
| ENSG00000105369 | CD79A | -0.19380345 | 4.91E-05 | 0.003054279 |
| ENSG00000152128 | TMEM163 | -0.193751493 | 4.94E-05 | 0.003060597 |
| ENSG00000214756 | METTL12 | 0.193633905 | 4.99E-05 | 0.003085396 |
| ENSG00000073849 | ST6GAL1 | -0.193581431 | 5.01E-05 | 0.003091954 |
| ENSG00000124493 | GRM4 | 0.193369611 | 5.11E-05 | 0.003127312 |
| ENSG00000152104 | PTPN14 | 0.193406935 | 5.10E-05 | 0.003133252 |
| ENSG00000221886 | C5orf54 | 0.193370498 | 5.11E-05 | 0.003135349 |
| ENSG00000076662 | ICAM3 | -0.193228152 | 5.18E-05 | 0.003159563 |
| ENSG00000130764 | LRRC47 | -0.193153727 | 5.21E-05 | 0.003164376 |
| ENSG00000106565 | TMEM176B | 0.193166513 | 5.21E-05 | 0.00316901 |
| ENSG00000242444 | RP11-320N7.1 | 0.193099848 | 5.24E-05 | 0.003171624 |
| ENSG00000259007 | RP11-463J10.3 | -0.193044417 | 5.27E-05 | 0.00317936 |
| ENSG00000105556 | MIER2 | 0.192928382 | 5.32E-05 | 0.003179926 |
| ENSG00000225611 | RP11-70C1.1 | 0.192940577 | 5.32E-05 | 0.00318463 |
| ENSG00000229107 | ABHD17AP4 | -0.192996895 | 5.29E-05 | 0.003184839 |
| ENSG00000171798 | KNDC1 | 0.192959202 | 5.31E-05 | 0.003187491 |
| ENSG00000242477 | CTD-2161E19.1 | -0.19282772 | 5.37E-05 | 0.003200925 |
| ENSG00000254923 | RP11-1236K1.8 | 0.192703851 | 5.43E-05 | 0.003228877 |
| ENSG00000149292 | TTC12 | -0.192493214 | 5.54E-05 | 0.00328285 |
| ENSG00000169245 | CXCL10 | -0.192298171 | 5.64E-05 | 0.003332956 |

| | | | | |
|---|---|---|---|---|
| ENSG00000260214 | #N/A | -0.1919579 | 5.81E-05 | 0.003402587 |
| ENSG00000130518 | KIAA1683 | 0.192020721 | 5.78E-05 | 0.003409162 |
| ENSG00000203852 | HIST2H3A | 0.191963886 | 5.81E-05 | 0.003409368 |
| ENSG00000171873 | ADRA1D | 0.19198628 | 5.80E-05 | 0.003411119 |
| ENSG00000162704 | ARPC5 | -0.191768474 | 5.91E-05 | 0.00342675 |
| ENSG00000136848 | DAB2IP | -0.191791977 | 5.90E-05 | 0.003428057 |
| ENSG00000187837 | HIST1H1C | 0.191834548 | 5.88E-05 | 0.003432122 |
| ENSG00000155629 | PIK3AP1 | -0.191801585 | 5.90E-05 | 0.003433704 |
| ENSG00000131055 | COX4I2 | -0.191460796 | 6.08E-05 | 0.003497145 |
| ENSG00000130300 | PLVAP | 0.191469961 | 6.08E-05 | 0.003502966 |
| ENSG00000137766 | UNC13C | -0.191478534 | 6.07E-05 | 0.003509001 |
| ENSG00000142347 | MYO1F | -0.191367894 | 6.13E-05 | 0.003517872 |
| ENSG00000197872 | FAM49A | -0.191294061 | 6.17E-05 | 0.003532635 |
| ENSG00000212768 | AC114546.1 | 0.191229539 | 6.21E-05 | 0.00353577 |
| ENSG00000176641 | RNF152 | -0.191229687 | 6.21E-05 | 0.003544454 |
| ENSG00000203811 | HIST2H3C | 0.191149497 | 6.25E-05 | 0.003552653 |
| ENSG00000262202 | SNORD3D | 0.191050313 | 6.31E-05 | 0.003575789 |
| ENSG00000122122 | SASH3 | -0.190914175 | 6.39E-05 | 0.003602284 |
| ENSG00000261207 | LA16c-361A3.3 | 0.19092467 | 6.38E-05 | 0.003607682 |
| ENSG00000134138 | MEIS2 | -0.190587281 | 6.58E-05 | 0.003700859 |
| ENSG00000178403 | NEUROG2 | -0.19045942 | 6.65E-05 | 0.003734579 |
| ENSG00000198841 | KTI12 | -0.190379238 | 6.70E-05 | 0.003752482 |
| ENSG00000206418 | RAB12 | -0.190254622 | 6.78E-05 | 0.003776441 |
| ENSG00000110042 | DTX4 | -0.190267267 | 6.77E-05 | 0.003781262 |
| ENSG00000169398 | PTK2 | -0.189839261 | 7.04E-05 | 0.003910397 |
| ENSG00000175348 | TMEM9B | -0.189802011 | 7.06E-05 | 0.003914061 |
| ENSG00000175215 | CTDSP2 | -0.189719161 | 7.11E-05 | 0.003933788 |
| ENSG00000089063 | TMEM230 | -0.189484651 | 7.26E-05 | 0.004007644 |
| ENSG00000111262 | KCNA1 | -0.189425377 | 7.30E-05 | 0.004019358 |
| ENSG00000141316 | SPACA3 | 0.189337279 | 7.36E-05 | 0.004041517 |
| ENSG00000100422 | CERK | -0.189293748 | 7.39E-05 | 0.00404766 |
| ENSG00000187239 | FNBP1 | -0.189233956 | 7.43E-05 | 0.00405972 |
| ENSG00000241566 | IGKV2D-24 | -0.189131816 | 7.50E-05 | 0.004087266 |
| ENSG00000174607 | UGT8 | -0.188637225 | 7.83E-05 | 0.004261526 |
| ENSG00000149289 | ZC3H12C | 0.188554449 | 7.89E-05 | 0.004262962 |
| ENSG00000137266 | SLC22A23 | -0.188596872 | 7.86E-05 | 0.004266829 |
| ENSG00000180353 | HCLS1 | -0.188561839 | 7.89E-05 | 0.004270135 |
| ENSG00000100612 | DHRS7 | -0.188378179 | 8.02E-05 | 0.004290266 |
| ENSG00000254423 | RP11-351I21.7 | 0.188384978 | 8.01E-05 | 0.004297618 |
| ENSG00000231747 | AC079922.2 | -0.188403751 | 8.00E-05 | 0.004300423 |
| ENSG00000189190 | ZNF600 | 0.188428584 | 7.98E-05 | 0.004300935 |
| ENSG00000145936 | KCNMB1 | -0.188111815 | 8.21E-05 | 0.004382906 |
| ENSG00000188452 | CERKL | 0.187907017 | 8.36E-05 | 0.004453041 |
| ENSG00000198515 | CNGA1 | -0.187835696 | 8.41E-05 | 0.004471003 |
| ENSG00000172985 | SH3RF3 | -0.187780487 | 8.45E-05 | 0.00448264 |
| ENSG00000173210 | ABLIM3 | 0.187736733 | 8.49E-05 | 0.004489769 |
| ENSG00000159128 | IFNGR2 | -0.187670512 | 8.54E-05 | 0.004505887 |
| ENSG00000164938 | TP53INP1 | -0.187584188 | 8.60E-05 | 0.004530142 |
| ENSG00000168477 | TNXB | 0.187393802 | 8.75E-05 | 0.00459668 |
| ENSG00000188873 | RPL10AP2 | -0.187174739 | 8.92E-05 | 0.004675981 |
| ENSG00000023697 | DERA | -0.187085311 | 8.99E-05 | 0.004691809 |
| ENSG00000120875 | DUSP4 | 0.187110291 | 8.97E-05 | 0.004692055 |
| ENSG00000101210 | EEF1A2 | -0.18670779 | 9.29E-05 | 0.004785966 |
| ENSG00000135077 | HAVCR2 | -0.186730775 | 9.27E-05 | 0.004786935 |
| ENSG00000160179 | ABCG1 | 0.186821164 | 9.20E-05 | 0.004791701 |
| ENSG00000249242 | TMEM150C | 0.186731884 | 9.27E-05 | 0.004797174 |
| ENSG00000105492 | SIGLEC6 | -0.186754869 | 9.26E-05 | 0.004798193 |
| ENSG00000168542 | COL3A1 | 0.186756126 | 9.25E-05 | 0.004808441 |
| ENSG00000175793 | SFN | 0.186540832 | 9.43E-05 | 0.004835379 |
| ENSG00000224521 | RP11-438F14.3 | -0.186560787 | 9.41E-05 | 0.004837611 |
| ENSG00000159450 | TCHH | 0.18629568 | 9.64E-05 | 0.004929953 |
| ENSG00000198873 | GRK5 | -0.186066197 | 9.83E-05 | 0.005019352 |
| ENSG00000091317 | CMTM6 | -0.185844252 | 0.000100274 | 0.005106906 |
| ENSG00000196352 | CD55 | -0.185671308 | 0.000101807 | 0.005173586 |
| ENSG00000240024 | LINC00888 | -0.185616468 | 0.000102298 | 0.005175786 |
| ENSG00000234264 | RP4-694A7.4 | 0.185640495 | 0.000102082 | 0.005176184 |
| ENSG00000104998 | IL27RA | 0.185552908 | 0.000102869 | 0.005193312 |
| ENSG00000262167 | #N/A | 0.18545195 | 0.000103784 | 0.005228091 |
| ENSG00000141179 | PCTP | -0.185238061 | 0.000105745 | 0.005315295 |
| ENSG00000175841 | FAM172BP | -0.185133925 | 0.000106713 | 0.005340732 |
| ENSG00000095002 | MSH2 | 0.185142941 | 0.000106629 | 0.005348104 |
| ENSG00000141367 | CLTC | 0.185043388 | 0.000107561 | 0.005371545 |
| ENSG00000203780 | FANK1 | -0.185014638 | 0.000107832 | 0.005373473 |
| ENSG00000105053 | VRK3 | -0.184883821 | 0.000109072 | 0.005423576 |
| ENSG00000127540 | UQCR11 | -0.184834303 | 0.000109544 | 0.005435357 |
| ENSG00000163040 | CCDC74A | -0.184738149 | 0.000110468 | 0.005469467 |
| ENSG00000153551 | CMTM7 | -0.184711542 | 0.000110725 | 0.005470477 |
| ENSG00000233138 | RP1-67K17.3 | -0.184646651 | 0.000111353 | 0.005489774 |
| ENSG00000081377 | CDC14B | -0.184599941 | 0.000111808 | 0.005500478 |
| ENSG00000168785 | TSPAN5 | 0.184546358 | 0.000112332 | 0.005514523 |
| ENSG00000067596 | DHX8 | -0.184505339 | 0.000112734 | 0.005522533 |
| ENSG00000100220 | RTCB | -0.184404308 | 0.000113731 | 0.005536186 |
| ENSG00000104140 | RHOV | -0.184413768 | 0.000113638 | 0.005543329 |
| ENSG00000189339 | SLC35E2B | -0.184417389 | 0.000113602 | 0.005553288 |
| ENSG00000235172 | CTB-114C7.3 | -0.184303941 | 0.00011473 | 0.005573082 |
| ENSG00000198468 | FLVCR1-AS1 | 0.184254127 | 0.000115229 | 0.005585587 |
| ENSG00000226981 | ABHD17AP6 | -0.184204461 | 0.000115728 | 0.005598039 |
| ENSG00000102034 | ELF4 | -0.184049329 | 0.0001173 | 0.005650439 |
| ENSG00000211652 | IGLV7-43 | 0.184067584 | 0.000117114 | 0.005653257 |
| ENSG00000254056 | IGHV3-71 | -0.183889097 | 0.000118946 | 0.005717816 |
| ENSG00000124635 | HIST1H2BJ | 0.183732117 | 0.000120579 | 0.005748511 |
| ENSG00000116983 | HPCAL4 | 0.183775574 | 0.000120125 | 0.00575058 |
| ENSG00000230493 | AC106017.1 | 0.183785404 | 0.000120022 | 0.00575757 |
| ENSG00000261423 | RP11-1007O24.3 | 0.183737069 | 0.000120527 | 0.005757904 |
| ENSG00000105642 | KCNN1 | -0.183600857 | 0.00012196 | 0.005802385 |
| ENSG00000197665 | AC007952.6 | 0.183525101 | 0.000122764 | 0.005816699 |
| ENSG00000104728 | ARHGEF10 | 0.183528649 | 0.000122727 | 0.005826886 |
| ENSG00000142684 | ZNF593 | -0.183467231 | 0.000123382 | 0.005834026 |
| ENSG00000166974 | MAPRE2 | -0.183357109 | 0.000124565 | 0.005842174 |
| ENSG00000176390 | CRLF3 | -0.183376694 | 0.000124354 | 0.005844132 |
| ENSG00000142784 | WDTC1 | -0.183297687 | 0.000125208 | 0.005848605 |
| ENSG00000174500 | GCSAM | -0.183413205 | 0.000123961 | 0.005849441 |
| ENSG00000174749 | C4orf32 | -0.183317494 | 0.000124994 | 0.005850428 |
| ENSG00000117289 | TXNIP | -0.183386228 | 0.000124251 | 0.005851185 |
| ENSG00000112584 | FAM120B | -0.183245656 | 0.000125774 | 0.005863198 |
| ENSG00000163507 | KIAA1524 | 0.183190077 | 0.00012638 | 0.005879594 |
| ENSG00000138411 | HECW2 | -0.183152015 | 0.000126798 | 0.005887195 |
| ENSG00000065882 | TBC1D1 | -0.183119348 | 0.000127157 | 0.005892032 |
| ENSG00000141668 | CBLN2 | -0.182637691 | 0.000132562 | 0.006130197 |
| ENSG00000112378 | PERP | -0.182571026 | 0.000133327 | 0.006153267 |
| ENSG00000170915 | PAQR8 | -0.182443535 | 0.000134801 | 0.006208902 |
| ENSG00000073464 | CLCN4 | -0.182240141 | 0.000137185 | 0.006256394 |
| ENSG00000167528 | ZNF641 | -0.182253075 | 0.000137033 | 0.006261812 |
| ENSG00000069345 | DNAJA2 | -0.182205257 | 0.000137598 | 0.006262876 |
| ENSG00000226360 | RPL10AP6 | -0.182253297 | 0.00013703 | 0.006274075 |
| ENSG00000187268 | FAM9C | -0.182257805 | 0.000136977 | 0.006284092 |
| ENSG00000182287 | AP1S2 | 0.182262757 | 0.000136918 | 0.006293873 |
| ENSG00000110077 | MS4A6A | -0.182116715 | 0.000138651 | 0.006298406 |
| ENSG00000217825 | AC099552.4 | -0.182054855 | 0.000139391 | 0.006319605 |
| ENSG00000109062 | SLC9A3R1 | -0.181937933 | 0.000140801 | 0.006358595 |
| ENSG00000249096 | RP11-290F5.1 | -0.181950497 | 0.000140649 | 0.006364161 |
| ENSG00000178562 | CD28 | -0.18179603 | 0.000142529 | 0.006411587 |
| ENSG00000163590 | PPM1L | -0.181797212 | 0.000142515 | 0.006423454 |
| ENSG00000109943 | CRTAM | -0.181705862 | 0.000143638 | 0.006448928 |
| ENSG00000196935 | SRGAP1 | -0.181573567 | 0.000145279 | 0.006509963 |
| ENSG00000261211 | RP1-80N2.3 | -0.18134268 | 0.000148185 | 0.006627338 |
| ENSG00000183454 | GRIN2A | -0.181219697 | 0.000149755 | 0.006684624 |
| ENSG00000112144 | ICK | -0.18115961 | 0.000150528 | 0.006706182 |
| ENSG00000136295 | TTYH3 | 0.181067817 | 0.000151716 | 0.00674611 |
| ENSG00000122025 | FLT3 | -0.180973141 | 0.00015295 | 0.006761969 |
| ENSG00000132170 | PPARG | 0.181013495 | 0.000152423 | 0.006764539 |
| ENSG00000145779 | TNFAIP8 | 0.180989622 | 0.000152735 | 0.0067654 |

| | | | | |
|---|---|---|---|---|
| ENSG00000089057 | SLC23A2 | -0.180862132 | 0.00015441 | 0.006813489 |
| ENSG00000215458 | AP001053.11 | -0.180703969 | 0.000156511 | 0.006866883 |
| ENSG00000107796 | ACTA2 | 0.180724515 | 0.000156237 | 0.006867893 |
| ENSG00000177383 | MAGEF1 | -0.180740553 | 0.000156023 | 0.00687155 |
| ENSG00000250081 | CTD-2116N20.1 | 0.180672632 | 0.000156931 | 0.00687227 |
| ENSG00000060709 | RIMBP2 | -0.180632131 | 0.000157475 | 0.006883057 |
| ENSG00000138801 | PAPSS1 | -0.180601015 | 0.000157894 | 0.006888349 |
| ENSG00000158106 | RHPN1 | -0.180382692 | 0.000160864 | 0.006978419 |
| ENSG00000151150 | ANK3 | 0.18034729 | 0.00016135 | 0.006986395 |
| ENSG00000214110 | LDHAP4 | 0.180387718 | 0.000160795 | 0.006988538 |
| ENSG00000234289 | H2BFS | 0.180395256 | 0.000160691 | 0.00699717 |
| ENSG00000170209 | ANKK1 | -0.180282104 | 0.000162249 | 0.006999107 |
| ENSG00000048052 | HDAC9 | -0.180289347 | 0.000162149 | 0.007007868 |
| ENSG00000116668 | SWT1 | 0.180097039 | 0.000164828 | 0.007070785 |
| ENSG00000169738 | DCXR | -0.180117437 | 0.000164542 | 0.007071636 |
| ENSG00000184979 | USP18 | -0.180119359 | 0.000164515 | 0.007083642 |
| ENSG00000172818 | OVOL1 | 0.179978195 | 0.000166505 | 0.007116319 |
| ENSG00000144820 | GPR128 | 0.179990242 | 0.000166334 | 0.007122175 |
| ENSG00000162390 | ACOT11 | 0.179851295 | 0.000168312 | 0.007180277 |
| ENSG00000215068 | AC025171.1 | -0.179766302 | 0.000169533 | 0.007205776 |
| ENSG00000167178 | ISLR2 | 0.179777757 | 0.000169368 | 0.00721202 |
| ENSG00000125166 | GOT2 | -0.179715083 | 0.000170273 | 0.007223949 |
| ENSG00000104043 | ATP8B4 | 0.179658175 | 0.000171099 | 0.007245698 |
| ENSG00000197006 | METTL9 | -0.179625138 | 0.00017158 | 0.007252784 |
| ENSG00000083828 | ZNF586 | -0.179576137 | 0.000172295 | 0.007269717 |
| ENSG00000095015 | MAP3K1 | -0.179547313 | 0.000172718 | 0.007274291 |
| ENSG00000136305 | CIDEB | -0.179391663 | 0.000175015 | 0.007357631 |
| ENSG00000230061 | AP001065.2 | -0.179331724 | 0.000175907 | 0.007381709 |
| ENSG00000180530 | NRIP1 | -0.179271563 | 0.000176807 | 0.007406035 |
| ENSG00000255507 | RP11-535A19.2 | 0.17908332 | 0.00017965 | 0.007511514 |
| ENSG00000157064 | NMNAT2 | -0.178749553 | 0.000184798 | 0.007698918 |
| ENSG00000109846 | CRYAB | 0.178702695 | 0.000185531 | 0.007701702 |
| ENSG00000197903 | HIST1H2BK | 0.178711712 | 0.00018539 | 0.00770969 |
| ENSG00000181023 | OR56B1 | 0.178752731 | 0.000184748 | 0.007710728 |
| ENSG00000056972 | TRAF3IP2 | -0.178589838 | 0.000187309 | 0.007761575 |
| ENSG00000218027 | RP11-157J24.1 | -0.178512531 | 0.000188536 | 0.007798443 |
| ENSG00000103769 | RAB11A | -0.17849095 | 0.00018888 | 0.00779872 |
| ENSG00000131389 | SLC6A6 | -0.178342765 | 0.000191258 | 0.00788283 |
| ENSG00000107263 | RAPGEF1 | -0.178125254 | 0.000194798 | 0.008014447 |
| ENSG00000134086 | VHL | -0.178075071 | 0.000195624 | 0.008034135 |
| ENSG00000158458 | NRG2 | -0.177908778 | 0.000198383 | 0.008133 |
| ENSG00000154822 | PLCL2 | -0.177666657 | 0.000202465 | 0.008285656 |
| ENSG00000118971 | CCND2 | 0.177594301 | 0.0002037 | 0.008306793 |
| ENSG00000139219 | COL2A1 | 0.177611004 | 0.000203414 | 0.008309785 |
| ENSG00000156427 | FGF18 | 0.177568064 | 0.00020415 | 0.008310486 |
| ENSG00000140006 | WDR89 | 0.1775453 | 0.000204541 | 0.00831177 |
| ENSG00000176177 | ENTHD1 | -0.177515737 | 0.000205049 | 0.008317795 |
| ENSG00000145391 | SETD7 | -0.177476196 | 0.000205731 | 0.008330844 |
| ENSG00000169641 | LUZP1 | -0.177379229 | 0.000207413 | 0.008384272 |
| ENSG00000156876 | SASS6 | 0.17726689 | 0.000209378 | 0.008448932 |
| ENSG00000241111 | RP11-129B22.1 | -0.177239544 | 0.000209859 | 0.008453588 |
| ENSG00000198046 | ZNF667 | -0.177201629 | 0.000210527 | 0.008465748 |
| ENSG00000181038 | METTL23 | -0.177176057 | 0.000210979 | 0.008469195 |
| ENSG00000107362 | ABHD17B | -0.177102297 | 0.000212288 | 0.008477587 |
| ENSG00000175390 | EIF3F | -0.177116709 | 0.000212031 | 0.008481974 |
| ENSG00000203497 | PDCD4-AS1 | -0.177075395 | 0.000212767 | 0.008482067 |
| ENSG00000224272 | AC114730.3 | 0.177049527 | 0.000213229 | 0.008485854 |
| ENSG00000182759 | MAFA | 0.177006882 | 0.000213992 | 0.008487003 |
| ENSG00000243467 | #N/A | -0.177129569 | 0.000211803 | 0.008487537 |
| ENSG00000262319 | CTC-457L16.2 | 0.177022994 | 0.000213703 | 0.008490104 |
| ENSG00000068137 | PLEKHH3 | 0.176904446 | 0.000215835 | 0.00854544 |
| ENSG00000124092 | CTCFL | -0.176869414 | 0.000216469 | 0.008555891 |
| ENSG00000065833 | ME1 | 0.176843842 | 0.000216933 | 0.008559599 |
| ENSG00000158856 | DMTN | 0.176759661 | 0.000218467 | 0.008576221 |
| ENSG00000146540 | C7orf50 | -0.176773334 | 0.000218217 | 0.008580975 |
| ENSG00000163995 | ABLIM2 | 0.176787302 | 0.000217962 | 0.008585549 |
| ENSG00000198756 | COLGALT2 | -0.176626479 | 0.000220914 | 0.008657582 |
| ENSG00000164683 | HEY1 | -0.176566466 | 0.000222025 | 0.008686399 |
| ENSG00000183283 | DAZAP2 | -0.176401282 | 0.00022511 | 0.008792219 |
| ENSG00000085741 | WNT11 | 0.176360485 | 0.000225878 | 0.008807337 |
| ENSG00000163060 | TEKT4 | -0.176336983 | 0.000226322 | 0.008809793 |
| ENSG00000168843 | FSTL5 | -0.17625428 | 0.000227889 | 0.008841023 |
| ENSG00000107742 | SPOCK2 | -0.176259453 | 0.000227791 | 0.008852073 |
| ENSG00000233971 | RPS20P10 | -0.17621533 | 0.000228631 | 0.008854951 |
| ENSG00000122861 | PLAU | -0.176120655 | 0.000230443 | 0.008910206 |
| ENSG00000010295 | IFFO1 | -0.176099369 | 0.000230852 | 0.008911118 |
| ENSG00000128708 | HAT1 | 0.175908539 | 0.000234552 | 0.009038852 |
| ENSG00000179981 | TSHZ1 | -0.175847861 | 0.00023574 | 0.009054452 |
| ENSG00000158516 | CPA2 | -0.175862495 | 0.000235453 | 0.009058476 |
| ENSG00000169413 | RNASE6 | -0.17581815 | 0.000236324 | 0.00906183 |
| ENSG00000258707 | RP11-467L19.11 | -0.175738034 | 0.000237905 | 0.009092297 |
| ENSG00000134954 | ETS1 | -0.175747871 | 0.00023771 | 0.009099885 |
| ENSG00000138792 | ENPEP | 0.175681864 | 0.000239019 | 0.009119798 |
| ENSG00000115652 | UXS1 | -0.175627321 | 0.000240106 | 0.009146179 |
| ENSG00000081237 | PTPRC | -0.175590958 | 0.000240833 | 0.009158784 |
| ENSG00000140009 | ESR2 | 0.175467089 | 0.000243325 | 0.009238359 |
| ENSG00000249274 | PDLIM1P4 | -0.175295031 | 0.000246827 | 0.009355957 |
| ENSG00000131773 | KHDRBS3 | -0.174975528 | 0.000253454 | 0.009591429 |
| ENSG00000166770 | ZNF667-AS1 | -0.174933549 | 0.000254337 | 0.009593442 |
| ENSG00000126351 | THRA | 0.174935396 | 0.000254298 | 0.009607644 |
| ENSG00000226397 | C12orf77 | 0.17480081 | 0.000257149 | 0.009683712 |
| ENSG00000166341 | DCHS1 | 0.174753879 | 0.000258149 | 0.009705563 |
| ENSG00000236090 | LDHAP3 | 0.17470348 | 0.000259228 | 0.009730308 |
| ENSG00000179299 | NSUN7 | 0.174657282 | 0.000260221 | 0.00975175 |
| ENSG00000226525 | RPS7P10 | -0.174607985 | 0.000261284 | 0.009775742 |
| ENSG00000111537 | IFNG | 0.174436741 | 0.000265009 | 0.009867211 |
| ENSG00000167470 | MIDN | -0.174448862 | 0.000264743 | 0.009873206 |
| ENSG00000137868 | STRA6 | -0.174465934 | 0.00026437 | 0.009875223 |
| ENSG00000259863 | SH3RF3-AS1 | -0.174358546 | 0.000266726 | 0.009899259 |
| ENSG00000126432 | PRDX5 | -0.174358768 | 0.000266721 | 0.009914989 |
| ENSG00000152749 | GPR180 | 0.173984573 | 0.000275083 | 0.010193059 |
| ENSG00000147485 | PXDNL | -0.173954788 | 0.000275759 | 0.010201759 |
| ENSG00000164199 | GPR98 | -0.173912957 | 0.000276711 | 0.010220626 |
| ENSG00000137731 | FXYD2 | 0.173869573 | 0.000277702 | 0.010224563 |
| ENSG00000115085 | ZAP70 | -0.173879107 | 0.000277484 | 0.010232831 |
| ENSG00000100351 | GRAP2 | -0.17380483 | 0.000279186 | 0.01026286 |
| ENSG00000113552 | GNPDA1 | 0.173560571 | 0.000284854 | 0.010454594 |
| ENSG00000137309 | HMGA1 | 0.173498778 | 0.000286305 | 0.010474595 |
| ENSG00000131370 | SH3BP5 | -0.173512894 | 0.000285973 | 0.01047903 |
| ENSG00000111879 | FAM184A | 0.173457242 | 0.000287284 | 0.010493808 |
| ENSG00000043462 | LCP2 | -0.173431226 | 0.000287899 | 0.010499686 |
| ENSG00000109654 | TRIM2 | 0.173335959 | 0.000290161 | 0.010565516 |
| ENSG00000230097 | RP11-460C6.1 | 0.173275059 | 0.000291615 | 0.010601764 |
| ENSG00000104419 | NDRG1 | 0.173130052 | 0.000295106 | 0.010711838 |
| ENSG00000079112 | CDH17 | -0.172961764 | 0.000299206 | 0.010809752 |
| ENSG00000129277 | CCL4 | 0.172996501 | 0.000298356 | 0.010812833 |
| ENSG00000020633 | RUNX3 | -0.172965607 | 0.000299112 | 0.010823267 |
| ENSG00000128833 | MYO5C | 0.172774186 | 0.000303839 | 0.010960008 |
| ENSG00000245812 | RP11-175K6.1 | -0.17272659 | 0.000305025 | 0.010968566 |
| ENSG00000008056 | SYN1 | -0.172745436 | 0.000304555 | 0.010968724 |
| ENSG00000214980 | RP11-274J2.1 | 0.172695327 | 0.000305807 | 0.010979611 |
| ENSG00000138030 | KHK | 0.172609594 | 0.000307959 | 0.011022644 |
| ENSG00000163563 | MNDA | 0.172620237 | 0.000307691 | 0.011030126 |
| ENSG00000095587 | TLL2 | -0.172456161 | 0.000311847 | 0.011144554 |
| ENSG00000256948 | RP11-598F7.3 | -0.172414773 | 0.000312904 | 0.011165071 |
| ENSG00000148516 | ZEB1 | 0.172386023 | 0.00031364 | 0.011174089 |
| ENSG00000179195 | ZNF664 | -0.172295042 | 0.000315979 | 0.011222836 |
| ENSG00000128274 | A4GALT | 0.172311598 | 0.000315552 | 0.011224913 |
| ENSG00000226937 | CEP164P1 | -0.172180707 | 0.000318942 | 0.011310701 |
| ENSG00000232531 | AC027612.1 | -0.172066963 | 0.000321915 | 0.011381221 |
| ENSG00000243802 | RP11-390K5.1 | -0.172075389 | 0.000321694 | 0.011390825 |
| ENSG00000225544 | KB-1027C11.4 | -0.17197044 | 0.000324459 | 0.01145365 |

| | | | | |
|---|---|---|---|---|
| ENSG00000262823 | RP13-580F15.2 | -0.171796609 | 0.000329087 | 0.011581658 |
| ENSG00000214820 | MPRIPP1 | -0.171807843 | 0.000328786 | 0.011588704 |
| ENSG00000105173 | CCNE1 | -0.171634603 | 0.000333455 | 0.011699767 |
| ENSG00000261026 | CTD-3247F14.2 | -0.171635046 | 0.000333443 | 0.011717126 |
| ENSG00000237350 | CDC42P6 | -0.17150556 | 0.000336973 | 0.011805287 |
| ENSG00000223417 | TRIM49D1 | 0.171386716 | 0.000340244 | 0.011901848 |
| ENSG00000175029 | CTBP2 | -0.171323156 | 0.000342005 | 0.011945377 |
| ENSG00000136888 | ATP6V1G1 | -0.171165806 | 0.000346402 | 0.012062511 |
| ENSG00000175970 | UNC119B | -0.171183544 | 0.000345904 | 0.012063337 |
| ENSG00000136485 | DCAF7 | -0.170989462 | 0.000351393 | 0.012163037 |
| ENSG00000181359 | HSP90AA6P | 0.171001361 | 0.000351054 | 0.012169521 |
| ENSG00000248746 | ACTN3 | -0.170960269 | 0.000352226 | 0.012173647 |
| ENSG00000243156 | MICAL3 | -0.171012004 | 0.000350751 | 0.012177274 |
| ENSG00000251102 | CTBP2P4 | -0.17102989 | 0.000350243 | 0.012177923 |
| ENSG00000242622 | RP11-18H7.1 | 0.170878157 | 0.000354577 | 0.012236611 |
| ENSG00000238041 | RP11-59K5.1 | -0.170847633 | 0.000355455 | 0.01224863 |
| ENSG00000253741 | CTD-2292P10.4 | 0.170795011 | 0.000356974 | 0.012264417 |
| ENSG00000149970 | CNKSR2 | -0.170805949 | 0.000356657 | 0.012271761 |
| ENSG00000204272 | RP11-622K12.1 | -0.170749336 | 0.000358296 | 0.012291573 |
| ENSG00000236125 | USP17L4 | 0.170404113 | 0.000368443 | 0.012620947 |
| ENSG00000180549 | FUT7 | 0.170366051 | 0.000369578 | 0.012641098 |
| ENSG00000158406 | HIST1H4H | 0.17033124 | 0.000370618 | 0.012657946 |
| ENSG00000204209 | DAXX | -0.170179729 | 0.00037518 | 0.012794855 |
| ENSG00000172349 | IL16 | -0.170054086 | 0.000379003 | 0.012887217 |
| ENSG00000231536 | AC123886.2 | -0.170070789 | 0.000378493 | 0.012888829 |
| ENSG00000158710 | TAGLN2 | -0.170024671 | 0.000379903 | 0.01289885 |
| ENSG00000125354 | 6-Sep | -0.169918687 | 0.000383163 | 0.012971442 |
| ENSG00000151414 | NEK7 | -0.169923787 | 0.000383006 | 0.012985139 |
| ENSG00000233837 | EIF3LP2 | -0.169654984 | 0.000391388 | 0.013230517 |
| ENSG00000135842 | FAM129A | -0.169550405 | 0.000394695 | 0.013322829 |
| ENSG00000058453 | CROCC | -0.169482262 | 0.000396864 | 0.01333763 |
| ENSG00000124074 | ENKD1 | -0.169485588 | 0.000396757 | 0.013353443 |
| ENSG00000182053 | TRIM49B | 0.169500887 | 0.00039627 | 0.013356494 |
| ENSG00000254471 | RP11-885L14.1 | 0.169194096 | 0.000406158 | 0.013630167 |
| ENSG00000174502 | SLC26A9 | -0.169160911 | 0.000407242 | 0.013646738 |
| ENSG00000140465 | CYP1A1 | 0.169121518 | 0.000408531 | 0.013670121 |
| ENSG00000214575 | CPEB1 | -0.169068896 | 0.000410259 | 0.013708105 |
| ENSG00000105655 | ISYNA1 | -0.169017752 | 0.000411946 | 0.013744611 |
| ENSG00000235204 | RP11-121A14.2 | 0.168975477 | 0.000413345 | 0.013771417 |
| ENSG00000251226 | RP11-469N6.1 | 0.168947392 | 0.000414276 | 0.013782575 |
| ENSG00000196743 | GM2A | 0.168857668 | 0.000417266 | 0.013862104 |
| ENSG00000154889 | MPPE1 | -0.168816723 | 0.000418637 | 0.013887697 |
| ENSG00000257576 | RP11-153M3.1 | 0.16879359 | 0.000419413 | 0.013893506 |
| ENSG00000223759 | RP11-353N4.4 | -0.168696401 | 0.00042269 | 0.013982029 |
| ENSG00000173473 | SMARCC1 | -0.168577785 | 0.000426721 | 0.014015118 |
| ENSG00000187775 | DNAH17 | -0.168584874 | 0.000426479 | 0.014027095 |
| ENSG00000159860 | FAM115D | 0.168598251 | 0.000426023 | 0.014032057 |
| ENSG00000172508 | CARNS1 | 0.168606825 | 0.000425731 | 0.014042442 |
| ENSG00000139517 | LNX2 | -0.168624415 | 0.000425132 | 0.014042717 |
| ENSG00000141446 | ESCO1 | 0.168430703 | 0.00043177 | 0.014160831 |
| ENSG00000188620 | HMX3 | -0.16837505 | 0.000433695 | 0.014203818 |
| ENSG00000234753 | RP11-328M4.2 | -0.168282074 | 0.000436928 | 0.014249153 |
| ENSG00000081985 | IL12RB2 | -0.168310455 | 0.000435939 | 0.014257117 |
| ENSG00000134962 | KLB | 0.168283109 | 0.000436892 | 0.014268103 |
| ENSG00000236852 | RP11-3D23.1 | 0.168219844 | 0.000439105 | 0.01429998 |
| ENSG00000114948 | ADAM23 | -0.168117556 | 0.000442704 | 0.014396908 |
| ENSG00000125753 | VASP | -0.16792643 | 0.000449504 | 0.014577043 |
| ENSG00000197697 | HIST1H2BE | 0.167942468 | 0.00044893 | 0.014578876 |
| ENSG00000115825 | PRKD3 | -0.167901375 | 0.000450402 | 0.014585707 |
| ENSG00000196331 | HIST1H2BO | 0.167846758 | 0.000452367 | 0.014608421 |
| ENSG00000249952 | #N/A | 0.167856218 | 0.000452026 | 0.014617825 |
| ENSG00000137193 | PIM1 | -0.167739074 | 0.000456264 | 0.014713719 |
| ENSG00000213015 | ZNF580 | -0.167631914 | 0.000460172 | 0.014819077 |
| ENSG00000259747 | RP11-275I4.2 | -0.167586972 | 0.000461821 | 0.014851495 |
| ENSG00000198738 | SMIM11P1 | -0.16745682 | 0.000466626 | 0.014964392 |
| ENSG00000227269 | RP11-96L7.2 | -0.167461033 | 0.000466469 | 0.014980134 |
| ENSG00000198276 | UCKL1 | -0.167339085 | 0.000471012 | 0.015084127 |
| ENSG00000135269 | TES | -0.167301762 | 0.000472411 | 0.015108004 |
| ENSG00000168676 | KCTD19 | -0.167124901 | 0.000479091 | 0.015279368 |
| ENSG00000160712 | IL6R | -0.16713813 | 0.000478588 | 0.015284408 |
| ENSG00000003436 | TFPI | -0.167043381 | 0.000482199 | 0.015336183 |
| ENSG00000104067 | TJP1 | 0.167050032 | 0.000481945 | 0.015349218 |
| ENSG00000227712 | RP11-418J17.3 | -0.167011822 | 0.000483408 | 0.015353516 |
| ENSG00000088832 | FKBP1A | -0.166957869 | 0.00048548 | 0.015398174 |
| ENSG00000247809 | NR2F2-AS1 | -0.166835774 | 0.000490201 | 0.015505373 |
| ENSG00000140470 | ADAMTS17 | 0.166852403 | 0.000489555 | 0.015506152 |
| ENSG00000140199 | SLC12A6 | -0.166582714 | 0.000500121 | 0.015775986 |
| ENSG00000177453 | NIM1K | 0.166592396 | 0.000499738 | 0.01578544 |
| ENSG00000262992 | #N/A | 0.166535708 | 0.000501984 | 0.01581318 |
| ENSG00000132819 | RBM38 | -0.166416421 | 0.00050674 | 0.015941282 |
| ENSG00000151623 | NR3C2 | 0.16636609 | 0.00050876 | 0.015983083 |
| ENSG00000108518 | PFN1 | -0.166301716 | 0.000511354 | 0.016042778 |
| ENSG00000214212 | C19orf38 | -0.166266019 | 0.000512798 | 0.016066281 |
| ENSG00000197696 | NMB | -0.166208223 | 0.000515143 | 0.016117911 |
| ENSG00000228549 | U1 | -0.166038088 | 0.000522106 | 0.016313696 |
| ENSG00000250274 | CTB-114C7.4 | -0.165859009 | 0.000529528 | 0.016523275 |
| ENSG00000109881 | CCDC34 | 0.165839128 | 0.000530358 | 0.01652687 |
| ENSG00000235999 | RP11-403I13.8 | -0.165709642 | 0.000535794 | 0.016651383 |
| ENSG00000092931 | MFSD11 | -0.165711341 | 0.000535723 | 0.016671584 |
| ENSG00000103647 | CORO2B | -0.165660641 | 0.000537865 | 0.016693308 |
| ENSG00000237437 | ASS1P12 | 0.165573577 | 0.000541562 | 0.016785518 |
| ENSG00000108883 | EFTUD2 | -0.165500557 | 0.000544681 | 0.016837051 |
| ENSG00000137225 | CAPN11 | 0.165511643 | 0.000544207 | 0.016844919 |
| ENSG00000106341 | PPP1R17 | 0.165445865 | 0.000547029 | 0.016887055 |
| ENSG00000173077 | 1-Dec | -0.165415267 | 0.000548346 | 0.016905142 |
| ENSG00000173198 | CYSLTR1 | -0.165394721 | 0.000549232 | 0.01690991 |
| ENSG00000260928 | SPCS2P1 | -0.165194061 | 0.000557956 | 0.01713288 |
| ENSG00000237753 | AC079922.3 | -0.165199826 | 0.000557704 | 0.017147915 |
| ENSG00000183558 | HIST2H2AA3 | 0.16509436 | 0.000562339 | 0.017221725 |
| ENSG00000169957 | ZNF768 | -0.165106554 | 0.000561801 | 0.017228067 |
| ENSG00000064393 | HIPK2 | -0.165000866 | 0.000566478 | 0.017302648 |
| ENSG00000182952 | HMGN4 | -0.165012987 | 0.000565939 | 0.01730905 |
| ENSG00000166959 | MS4A8 | -0.164892148 | 0.000571326 | 0.017427704 |
| ENSG00000100814 | CCNB1IP1 | -0.164873523 | 0.00057216 | 0.01743015 |
| ENSG00000152910 | CNTNAP4 | -0.164850242 | 0.000573205 | 0.017439008 |
| ENSG00000116824 | CD2 | -0.164817575 | 0.000574674 | 0.017460726 |
| ENSG00000173918 | C1QTNF1 | -0.164800059 | 0.000575463 | 0.017461753 |
| ENSG00000003400 | CASP10 | -0.164690676 | 0.000580412 | 0.017474331 |
| ENSG00000203812 | HIST2H2AA4 | 0.164692893 | 0.000580312 | 0.017494099 |
| ENSG00000254667 | AP000783.1 | -0.164702649 | 0.000579869 | 0.017503565 |
| ENSG00000142627 | EPHA2 | 0.164715213 | 0.000579298 | 0.017509187 |
| ENSG00000172500 | FIBP | -0.164715361 | 0.000579292 | 0.017531924 |
| ENSG00000235098 | ANKRD65 | -0.16471743 | 0.000579198 | 0.017552053 |
| ENSG00000233154 | RP4-655J12.4 | 0.164505167 | 0.000588897 | 0.017706731 |
| ENSG00000182871 | COL18A1 | -0.164473239 | 0.000590369 | 0.017727938 |
| ENSG00000119888 | EPCAM | -0.164384698 | 0.000594469 | 0.017827902 |
| ENSG00000257086 | RP11-783K16.13 | -0.164312859 | 0.000597814 | 0.017904994 |
| ENSG00000112406 | HECA | -0.164258611 | 0.000600352 | 0.017957748 |
| ENSG00000132823 | OSER1 | -0.164166226 | 0.000604698 | 0.0179715 |
| ENSG00000090104 | RGS1 | 0.164179308 | 0.00060408 | 0.017976239 |
| ENSG00000171540 | OTP | -0.164205989 | 0.000602824 | 0.017985157 |
| ENSG00000180336 | C17orf104 | 0.164188768 | 0.000603635 | 0.017986145 |
| ENSG00000078114 | NEBL | -0.16421604 | 0.000602351 | 0.017994263 |
| ENSG00000251562 | MALAT1 | 0.164008286 | 0.000612194 | 0.018170924 |
| ENSG00000134853 | PDGFRA | -0.163990769 | 0.00061303 | 0.01817241 |
| ENSG00000101363 | MANBAL | -0.163956994 | 0.000614647 | 0.018173744 |
| ENSG00000162676 | GFI1 | 0.163973105 | 0.000613875 | 0.018174158 |
| ENSG00000154258 | ABCA9 | 0.163841476 | 0.000620204 | 0.018291272 |
| ENSG00000230438 | RP11-420G6.4 | -0.16385404 | 0.000619598 | 0.018296737 |
| ENSG00000121900 | TMEM54 | -0.163785232 | 0.000622927 | 0.018348176 |
| ENSG00000102738 | MRPS31 | 0.163753008 | 0.000624492 | 0.01837087 |
| ENSG00000240694 | PNMA2 | -0.163692256 | 0.000627453 | 0.018434521 |

| | | | | |
|---|---|---|---|---|
| ENSG00000197321 | SVIL | 0.163632834 | 0.000630361 | 0.018496456 |
| ENSG00000261668 | RP11-50D9.3 | 0.163612805 | 0.000631344 | 0.01850182 |
| ENSG00000196547 | MAN2A2 | -0.163472381 | 0.000638276 | 0.018657671 |
| ENSG00000232101 | AC108059.2 | -0.163486423 | 0.00063758 | 0.018660917 |
| ENSG00000222047 | C10orf55 | -0.163439344 | 0.000639917 | 0.018682021 |
| ENSG00000010270 | STARD3NL | -0.163410741 | 0.000641341 | 0.018699983 |
| ENSG00000153936 | HS2ST1 | 0.163362628 | 0.000643743 | 0.01874638 |
| ENSG00000161956 | SENP3 | -0.163304388 | 0.000646662 | 0.018807697 |
| ENSG00000213326 | RPS7P11 | -0.163278077 | 0.000647985 | 0.018822499 |
| ENSG00000129355 | CDKN2D | -0.163204687 | 0.000651687 | 0.018906282 |
| ENSG00000126882 | FAM78A | -0.163136026 | 0.000655169 | 0.018983481 |
| ENSG00000230481 | IGKV1OR22-5 | -0.163034699 | 0.000660338 | 0.019085419 |
| ENSG00000234629 | WDR82P1 | -0.16304305 | 0.000659911 | 0.019096949 |
| ENSG00000127666 | TICAM1 | 0.163008462 | 0.000661683 | 0.019100417 |
| ENSG00000089041 | P2RX7 | 0.162988211 | 0.000662722 | 0.019106556 |
| ENSG00000159212 | CLIC6 | -0.162901887 | 0.000667171 | 0.019186975 |
| ENSG00000102230 | PCYT1B | -0.16290573 | 0.000666972 | 0.019205139 |
| ENSG00000151575 | TEX9 | 0.162867889 | 0.000668931 | 0.019213693 |
| ENSG00000147119 | CHST7 | 0.162819338 | 0.000671451 | 0.019214468 |
| ENSG00000135750 | KCNK1 | 0.162849782 | 0.00066987 | 0.019216792 |
| ENSG00000164237 | CMBL | 0.162827757 | 0.000671013 | 0.019225728 |
| ENSG00000124767 | GLO1 | -0.162738033 | 0.000675691 | 0.019288058 |
| ENSG00000204713 | TRIM27 | -0.162744389 | 0.000675359 | 0.019302411 |
| ENSG00000139998 | RAB15 | -0.162680681 | 0.000678698 | 0.019326176 |
| ENSG00000117602 | RCAN3 | -0.162681863 | 0.000678635 | 0.01934821 |
| ENSG00000215908 | CROCCP2 | -0.162524883 | 0.000686926 | 0.019464587 |
| ENSG00000156097 | GPR61 | 0.162567676 | 0.000684657 | 0.019471881 |
| ENSG00000071462 | WBSCR22 | 0.162499237 | 0.000688289 | 0.019479337 |
| ENSG00000114115 | RBP1 | -0.162526879 | 0.00068682 | 0.019485463 |
| ENSG00000185736 | ADARB2 | -0.162479282 | 0.000689352 | 0.019485571 |
| ENSG00000182217 | HIST2H4B | 0.162539148 | 0.000686169 | 0.019490909 |
| ENSG00000183941 | HIST2H4A | 0.162356669 | 0.000695914 | 0.019647037 |
| ENSG00000122497 | NBPF14 | -0.162329176 | 0.000697394 | 0.01966481 |
| ENSG00000226835 | RP11-148B18.3 | -0.162226666 | 0.000702935 | 0.01979691 |
| ENSG00000162496 | DHRS3 | 0.162202799 | 0.000704231 | 0.019809281 |
| ENSG00000183155 | RABIF | -0.162121569 | 0.000708659 | 0.019909615 |
| ENSG00000187486 | KCNJ11 | 0.162061999 | 0.000711922 | 0.019977015 |
| ENSG00000237520 | RP11-443B7.2 | -0.161850919 | 0.000723598 | 0.020230995 |
| ENSG00000138080 | EMILIN1 | -0.161873313 | 0.000722351 | 0.020245091 |
| ENSG00000115956 | PLEK | -0.161856018 | 0.000723314 | 0.020247538 |
| ENSG00000055813 | CCDC85A | -0.161808865 | 0.000725945 | 0.020247648 |
| ENSG00000236833 | AC024560.2 | 0.161811748 | 0.000725784 | 0.020267606 |
| ENSG00000105409 | ATP1A3 | 0.161747448 | 0.000729386 | 0.020319112 |
| ENSG00000214552 | COPS8P2 | -0.161624392 | 0.000736325 | 0.020487734 |
| ENSG00000257931 | CTD-2311B13.7 | -0.16160148 | 0.000737624 | 0.020499209 |
| ENSG00000137054 | POLR1E | -0.161533115 | 0.000741512 | 0.020582522 |
| ENSG00000129009 | ISLR | -0.161508135 | 0.000742937 | 0.020597349 |
| ENSG00000163814 | CDCP1 | 0.161405329 | 0.00074883 | 0.020686317 |
| ENSG00000180539 | C9orf139 | 0.161406807 | 0.000748745 | 0.02070871 |
| ENSG00000225177 | RP11-390P2.4 | -0.161419519 | 0.000748014 | 0.020713269 |
| ENSG00000238165 | AC007560.1 | -0.161372588 | 0.000750715 | 0.020713642 |
| ENSG00000125430 | HS3ST3B1 | -0.161355589 | 0.000751696 | 0.020715989 |
| ENSG00000146373 | RNF217 | 0.161259583 | 0.000757258 | 0.020844428 |
| ENSG00000147912 | FBXO10 | -0.161192105 | 0.000761189 | 0.020853332 |
| ENSG00000235609 | AF127936.7 | -0.161206222 | 0.000760365 | 0.020855468 |
| ENSG00000229214 | LINC00242 | 0.161207848 | 0.00076027 | 0.020877628 |
| ENSG00000139083 | ETV6 | -0.161211765 | 0.000760042 | 0.020896184 |
| ENSG00000166317 | SYNPO2L | -0.161131353 | 0.000764745 | 0.020925957 |
| ENSG00000149600 | COMMD7 | -0.161114354 | 0.000765743 | 0.020928498 |
| ENSG00000232157 | #N/A | -0.161061141 | 0.000768874 | 0.020939817 |
| ENSG00000109686 | SH3D19 | 0.1610808 | 0.000767716 | 0.02095765 |
| ENSG00000162341 | TPCN2 | 0.161062323 | 0.000768804 | 0.020962602 |
| ENSG00000100342 | APOL1 | -0.160823232 | 0.000783018 | 0.021150633 |
| ENSG00000122068 | FYTTD1 | -0.16083757 | 0.000782159 | 0.021152141 |
| ENSG00000169855 | ROBO1 | -0.160888123 | 0.000779137 | 0.021169454 |
| ENSG00000124602 | UNC5CL | 0.160872085 | 0.000780094 | 0.021170579 |
| ENSG00000250966 | RP11-402J6.3 | -0.160839491 | 0.000782044 | 0.021173796 |
| ENSG00000125122 | LRRC29 | 0.160900022 | 0.000778427 | 0.021175046 |
| ENSG00000263002 | ZNF234 | 0.16085346 | 0.000781208 | 0.021175957 |
| ENSG00000162032 | SPSB3 | -0.160783469 | 0.000785405 | 0.021190355 |
| ENSG00000180884 | ZNF792 | -0.160759449 | 0.000786851 | 0.021204626 |
| ENSG00000162928 | PEX13 | 0.160734395 | 0.000788361 | 0.021220586 |
| ENSG00000185028 | LRRC14B | 0.160707344 | 0.000789995 | 0.021239842 |
| ENSG00000250536 | ABHD17AP3 | -0.160647701 | 0.000793608 | 0.021287476 |
| ENSG00000140848 | CPNE2 | -0.160654205 | 0.000793213 | 0.021301592 |
| ENSG00000101017 | CD40 | -0.160623459 | 0.00079508 | 0.021302248 |
| ENSG00000158352 | SHROOM4 | 0.160580666 | 0.000797687 | 0.02134736 |
| ENSG00000226102 | SEPT7P3 | 0.160562337 | 0.000798805 | 0.021352566 |
| ENSG00000072195 | SPEG | -0.160479191 | 0.000803898 | 0.021463891 |
| ENSG00000175806 | MSRA | -0.160451845 | 0.00080558 | 0.021483992 |
| ENSG00000006659 | LGALS14 | -0.160315781 | 0.000813995 | 0.021658449 |
| ENSG00000146122 | DAAM2 | 0.160320511 | 0.000813701 | 0.02167557 |
| ENSG00000262465 | #N/A | -0.160246899 | 0.000818286 | 0.021747596 |
| ENSG00000253719 | ATXN7L3B | -0.160138107 | 0.000825106 | 0.021878556 |
| ENSG00000229047 | AF127577.10 | -0.160143502 | 0.000824766 | 0.021894649 |
| ENSG00000081059 | TCF7 | -0.160078168 | 0.000828885 | 0.021903405 |
| ENSG00000124557 | BTN1A1 | -0.160079276 | 0.000828815 | 0.021926614 |
| ENSG00000131187 | F12 | -0.160087702 | 0.000828283 | 0.02193764 |
| ENSG00000107099 | DOCK8 | -0.160022589 | 0.000832405 | 0.021971311 |
| ENSG00000261128 | #N/A | -0.159961172 | 0.00083631 | 0.022049213 |
| ENSG00000223638 | RFPL4A | 0.159916753 | 0.000839144 | 0.022073592 |
| ENSG00000205097 | FRG2 | 0.1599254 | 0.000838592 | 0.022084196 |
| ENSG00000112033 | PPARD | -0.159865683 | 0.000842414 | 0.022134428 |
| ENSG00000111729 | CLEC4A | -0.159647138 | 0.000856542 | 0.022403806 |
| ENSG00000197057 | DTHD1 | -0.159659185 | 0.000855757 | 0.022408622 |
| ENSG00000101474 | APMAP | -0.159660367 | 0.00085568 | 0.02243201 |
| ENSG00000184260 | HIST2H2AC | 0.159667462 | 0.000855219 | 0.022445373 |
| ENSG00000180901 | KCTD2 | -0.159591116 | 0.000860198 | 0.022474009 |
| ENSG00000110076 | NRXN2 | 0.159543519 | 0.000863316 | 0.022530014 |
| ENSG00000182606 | TRAK1 | -0.159463108 | 0.000868608 | 0.022642564 |
| ENSG00000111907 | TPD52L1 | 0.159369097 | 0.000874832 | 0.022779128 |
| ENSG00000108176 | DNAJC12 | -0.159281886 | 0.000880642 | 0.022878881 |
| ENSG00000139343 | SNRPF | 0.159289794 | 0.000880114 | 0.022890884 |
| ENSG00000106025 | TSPAN12 | -0.159217364 | 0.000884964 | 0.022939616 |
| ENSG00000164627 | KIF6 | 0.159194527 | 0.000886499 | 0.022953673 |
| ENSG00000231389 | HLA-DPA1 | 0.159223055 | 0.000884582 | 0.022955449 |
| ENSG00000060566 | CREB3L3 | 0.159087582 | 0.000893717 | 0.023114681 |
| ENSG00000073712 | FERMT2 | -0.15893415 | 0.000904168 | 0.02330677 |
| ENSG00000225899 | FRG2B | 0.158946271 | 0.000903338 | 0.023311363 |
| ENSG00000177738 | CTD-2201E18.3 | -0.158952405 | 0.000902919 | 0.023326584 |
| ENSG00000159055 | MIS18A | 0.158885223 | 0.000907524 | 0.023341235 |
| ENSG00000006116 | CACNG3 | -0.158886701 | 0.000907423 | 0.023364627 |
| ENSG00000262074 | SNORD3B-2 | 0.158856769 | 0.000909481 | 0.023365577 |
| ENSG00000123454 | DBH | 0.158837479 | 0.000910811 | 0.023373776 |
| ENSG00000112796 | ENPP5 | 0.15877377 | 0.000915213 | 0.023382934 |
| ENSG00000258756 | #N/A | 0.15881065 | 0.000912662 | 0.023395311 |
| ENSG00000260609 | #N/A | 0.15879173 | 0.00091397 | 0.023402895 |
| ENSG00000137501 | SYTL2 | 0.158775027 | 0.000915126 | 0.023406575 |
| ENSG00000185527 | PDE6G | -0.158680277 | 0.000921709 | 0.023522909 |
| ENSG00000259248 | USP3-AS1 | -0.158646723 | 0.00092405 | 0.023530709 |
| ENSG00000259132 | RP11-298I3.5 | -0.158653005 | 0.000923611 | 0.023545461 |
| ENSG00000176771 | NCKAP5 | 0.158580132 | 0.000928714 | 0.02362346 |
| ENSG00000134294 | SLC38A2 | -0.158558551 | 0.00093023 | 0.02363602 |
| ENSG00000233822 | HIST1H2BN | 0.158506889 | 0.000933868 | 0.02370241 |
| ENSG00000253629 | KB-1107E3.1 | -0.158489521 | 0.000935094 | 0.023707504 |
| ENSG00000250326 | RP11-284M14.1 | 0.158421156 | 0.000939935 | 0.023804137 |
| ENSG00000185670 | ZBTB3 | 0.158369495 | 0.000943609 | 0.023871036 |
| ENSG00000168303 | MPLKIP | -0.158308078 | 0.000947993 | 0.023877445 |
| ENSG00000137752 | CASP1 | -0.158348062 | 0.000945137 | 0.02388356 |
| ENSG00000135932 | CAB39 | 0.15831702 | 0.000947353 | 0.023887346 |
| ENSG00000112379 | KIAA1244 | 0.158325815 | 0.000946725 | 0.023897571 |
| ENSG00000165512 | ZNF22 | -0.15825516 | 0.000951786 | 0.023946894 |

| | | | | |
|---|---|---|---|---|
| ENSG00000067560 | RHOA | -0.158116656 | 0.000961779 | 0.024172015 |
| ENSG00000130332 | LSM7 | -0.158016068 | 0.000969097 | 0.024224282 |
| ENSG00000235385 | GS1-600G8.5 | -0.158057826 | 0.000966053 | 0.024226765 |
| ENSG00000251279 | CTC-436P18.1 | 0.158063665 | 0.000965628 | 0.0242424 |
| ENSG00000197272 | IL27 | 0.158017546 | 0.000968989 | 0.024247796 |
| ENSG00000189325 | C6orf222 | -0.158023385 | 0.000968563 | 0.024263395 |
| ENSG00000236534 | H3F3BP1 | -0.157957533 | 0.000973379 | 0.024305042 |
| ENSG00000166831 | RBPMS2 | 0.157941126 | 0.000974583 | 0.024308855 |
| ENSG00000116741 | RGS2 | 0.15790432 | 0.000977287 | 0.024350032 |
| ENSG00000182389 | CACNB4 | 0.157849258 | 0.000981346 | 0.024372376 |
| ENSG00000227304 | RP11-791G16.2 | -0.157861157 | 0.000980468 | 0.024376754 |
| ENSG00000176208 | ATAD5 | 0.157866331 | 0.000980086 | 0.024393486 |
| ENSG00000176396 | EID2 | -0.157821469 | 0.000983401 | 0.024397208 |
| ENSG00000168297 | PXK | -0.157786363 | 0.000986002 | 0.024435518 |
| ENSG00000198755 | RPL10A | -0.157722063 | 0.000990782 | 0.024527689 |
| ENSG00000126267 | COX6B1 | -0.157705138 | 0.000992044 | 0.024532665 |
| ENSG00000184678 | HIST2H2BE | 0.157640912 | 0.000996846 | 0.024625078 |
| ENSG00000105085 | MED26 | -0.157523547 | 0.001005677 | 0.024816717 |
| ENSG00000259401 | RP11-566K19.8 | -0.157486076 | 0.001008512 | 0.024860143 |
| ENSG00000215860 | PDZK1P1 | 0.157364571 | 0.001017754 | 0.025034583 |
| ENSG00000261435 | MIR1587 | 0.15737551 | 0.001016918 | 0.025040658 |
| ENSG00000163283 | ALPP | 0.157306184 | 0.001022222 | 0.025117765 |
| ENSG00000091844 | RGS17 | 0.157266718 | 0.001025253 | 0.025165499 |
| ENSG00000066322 | ELOVL1 | -0.157179359 | 0.001031992 | 0.02530405 |
| ENSG00000261324 | RP11-174G6.5 | 0.157162582 | 0.001033291 | 0.025309062 |
| ENSG00000168890 | TMEM150A | -0.157100942 | 0.001038075 | 0.025399333 |
| ENSG00000173542 | MOB1B | -0.15705985 | 0.001041276 | 0.025450723 |
| ENSG00000242802 | AP5Z1 | -0.156996733 | 0.001046211 | 0.025544341 |
| ENSG00000187715 | KBTBD12 | -0.156958301 | 0.001049226 | 0.025590932 |
| ENSG00000121769 | FABP3 | 0.156919499 | 0.001052278 | 0.025638327 |
| ENSG00000078804 | TP53INP2 | -0.156855864 | 0.001057302 | 0.025733618 |
| ENSG00000241294 | IGKV2-24 | -0.156840861 | 0.00105849 | 0.025735442 |
| ENSG00000100897 | DCAF11 | -0.156808194 | 0.00106108 | 0.025771315 |
| ENSG00000253744 | AC025442.3 | 0.156722387 | 0.001067911 | 0.025910009 |
| ENSG00000245648 | RP11-277P12.20 | -0.156637245 | 0.001074729 | 0.025993602 |
| ENSG00000135052 | GOLM1 | -0.156649884 | 0.001073715 | 0.02599627 |
| ENSG00000168314 | MOBP | 0.156650918 | 0.001073632 | 0.026021508 |
| ENSG00000261827 | RP11-340F14.5 | 0.15660613 | 0.001077231 | 0.026026892 |
| ENSG00000237550 | UBE2Q2P6 | -0.156561342 | 0.001080842 | 0.02603253 |
| ENSG00000242140 | RP11-262M14.2 | 0.156561786 | 0.001080806 | 0.026058807 |
| ENSG00000147647 | DPYS | -0.156573611 | 0.001079852 | 0.026062983 |
| ENSG00000196586 | MYO6 | 0.15649586 | 0.001086141 | 0.026132937 |
| ENSG00000244245 | RP11-120B7.1 | -0.156455432 | 0.001089424 | 0.02618468 |
| ENSG00000120370 | GORAB | -0.156426756 | 0.001091759 | 0.026213553 |
| ENSG00000167900 | TK1 | 0.156402071 | 0.001093772 | 0.026234643 |
| ENSG00000105339 | DENND3 | -0.156358983 | 0.001097294 | 0.026291846 |
| ENSG00000196734 | LCE1B | 0.15633112 | 0.001099578 | 0.026319299 |
| ENSG00000011083 | SLC6A7 | 0.156266598 | 0.001104882 | 0.026364377 |
| ENSG00000153956 | CACNA2D1 | -0.156268446 | 0.00110473 | 0.026387983 |
| ENSG00000180448 | HMHA1 | -0.156281158 | 0.001103683 | 0.026390236 |
| ENSG00000250850 | RP11-297B17.3 | -0.156180791 | 0.001111973 | 0.026506227 |
| ENSG00000256897 | RP11-17G12.2 | 0.156120852 | 0.001116951 | 0.026597468 |
| ENSG00000205002 | AARD | -0.156103779 | 0.001118372 | 0.026603907 |
| ENSG00000203835 | ABHD17AP1 | -0.156050714 | 0.001122802 | 0.026654443 |
| ENSG00000158525 | CPA5 | -0.156056035 | 0.001122357 | 0.026671263 |
| ENSG00000002933 | TMEM176A | 0.155945173 | 0.001131659 | 0.02678221 |
| ENSG00000005379 | BZRAP1 | -0.155951086 | 0.001131161 | 0.026797853 |
| ENSG00000163349 | HIPK1 | -0.155963281 | 0.001130135 | 0.026801007 |
| ENSG00000109103 | UNC119 | -0.15590482 | 0.001135063 | 0.026835303 |
| ENSG00000198658 | ABHD17AP2 | -0.155788199 | 0.001144953 | 0.027041474 |
| ENSG00000154065 | ANKRD29 | 0.155759887 | 0.001147366 | 0.027070813 |
| ENSG00000178927 | C17orf62 | -0.155708447 | 0.001151762 | 0.027146831 |
| ENSG00000185811 | IKZF1 | -0.1556389 | 0.00115773 | 0.027149121 |
| ENSG00000145741 | BTF3 | -0.155647621 | 0.00115698 | 0.027159078 |
| ENSG00000180438 | TPRXL | -0.155672602 | 0.001154834 | 0.027163857 |
| ENSG00000152939 | MARVELD2 | -0.155655603 | 0.001156294 | 0.027170559 |
| ENSG00000261845 | RP13-638C3.4 | -0.155676814 | 0.001154473 | 0.027183019 |
| ENSG00000113140 | SPARC | -0.155530477 | 0.00116709 | 0.027340887 |
| ENSG00000154945 | ANKRD40 | -0.155503353 | 0.001169443 | 0.02736828 |
| ENSG00000083454 | P2RX5 | -0.155361672 | 0.001181803 | 0.027629574 |
| ENSG00000110395 | CBL | -0.155297668 | 0.001187426 | 0.027732994 |
| ENSG00000261434 | CTD-2083E4.7 | 0.155275052 | 0.001189419 | 0.02775151 |
| ENSG00000259936 | #N/A | -0.155235733 | 0.001192891 | 0.027804461 |
| ENSG00000124766 | SOX4 | -0.155213856 | 0.001194827 | 0.027821541 |
| ENSG00000143815 | LBR | -0.155196636 | 0.001196352 | 0.027829025 |
| ENSG00000198467 | TPM2 | -0.155146083 | 0.001200842 | 0.027905396 |
| ENSG00000259330 | LINC00984 | -0.155070327 | 0.001207599 | 0.028034241 |
| ENSG00000171777 | RASGRP4 | -0.15498356 | 0.001215381 | 0.028073966 |
| ENSG00000120129 | DUSP1 | -0.15501837 | 0.001212253 | 0.028085886 |
| ENSG00000099282 | TSPAN15 | -0.1550316 | 0.001211066 | 0.028086528 |
| ENSG00000213376 | GAPDHP71 | 0.154985999 | 0.001215161 | 0.028096953 |
| ENSG00000154237 | LRRK1 | 0.154989177 | 0.001214875 | 0.028118458 |
| ENSG00000226677 | IGBP1P1 | -0.154907287 | 0.001222259 | 0.028204663 |
| ENSG00000189157 | FAM47E | 0.154886371 | 0.001224152 | 0.028220182 |
| ENSG00000133574 | GIMAP4 | -0.154756072 | 0.001236003 | 0.028408411 |
| ENSG00000183150 | GPR19 | 0.154781422 | 0.001233689 | 0.02841171 |
| ENSG00000258501 | EIF3LP1 | -0.154765532 | 0.001235139 | 0.0284168 |
| ENSG00000151917 | BEND6 | 0.154710027 | 0.001240216 | 0.028476936 |
| ENSG00000163866 | SMIM12 | -0.154680021 | 0.001242969 | 0.028483577 |
| ENSG00000125735 | TNFSF14 | 0.154689407 | 0.001242107 | 0.028492062 |
| ENSG00000153574 | RPIA | -0.15463272 | 0.001247319 | 0.028526716 |
| ENSG00000146054 | TRIM7 | 0.154637745 | 0.001246856 | 0.028544361 |
| ENSG00000102931 | ARL2BP | -0.154549727 | 0.001254987 | 0.028673725 |
| ENSG00000125637 | PSD4 | -0.154504342 | 0.001259198 | 0.028741536 |
| ENSG00000076513 | ANKRD13A | -0.154421787 | 0.001266892 | 0.028888636 |
| ENSG00000198265 | HELZ | -0.154312625 | 0.001277131 | 0.029007685 |
| ENSG00000111245 | MYL2 | 0.154313808 | 0.00127702 | 0.029033684 |
| ENSG00000232347 | RP11-488L18.8 | 0.15431706 | 0.001276714 | 0.029055296 |
| ENSG00000213782 | DDX47 | -0.15426089 | 0.001282011 | 0.02906143 |
| ENSG00000067798 | NAV3 | -0.154326668 | 0.00127581 | 0.029063329 |
| ENSG00000188070 | C11orf95 | -0.154270498 | 0.001281103 | 0.029069346 |
| ENSG00000154930 | ACSS1 | -0.154195334 | 0.001288218 | 0.029173532 |
| ENSG00000114737 | CISH | 0.15415668 | 0.001291891 | 0.029199515 |
| ENSG00000005893 | LAMP2 | 0.154129414 | 0.001294488 | 0.029201123 |
| ENSG00000179455 | MKRN3 | -0.154168727 | 0.001290745 | 0.029202158 |
| ENSG00000153982 | GDPD1 | -0.154111892 | 0.001296159 | 0.02921032 |
| ENSG00000186469 | GNG2 | -0.154132069 | 0.001294235 | 0.029223927 |
| ENSG00000259344 | RP11-566K19.6 | -0.154032293 | 0.001303777 | 0.02935339 |
| ENSG00000214535 | RPS15AP1 | -0.15400746 | 0.001306161 | 0.029378458 |
| ENSG00000240632 | SPATA31D5P | -0.153939243 | 0.001312733 | 0.029468944 |
| ENSG00000125910 | S1PR4 | -0.153947669 | 0.00131192 | 0.029479314 |
| ENSG00000261786 | RP4-555D20.2 | -0.153825505 | 0.001323758 | 0.029658849 |
| ENSG00000148908 | RGS10 | -0.153830451 | 0.001323277 | 0.029676829 |
| ENSG00000104886 | PLEKHJ1 | -0.153776868 | 0.001328499 | 0.029736257 |
| ENSG00000174173 | TRMT10C | 0.153757214 | 0.001330419 | 0.029750433 |
| ENSG00000236048 | AC013470.6 | 0.153671845 | 0.001338788 | 0.029764862 |
| ENSG00000107614 | TRDMT1 | 0.153707691 | 0.001335268 | 0.029772485 |
| ENSG00000215014 | AL645728.1 | 0.153678423 | 0.001338142 | 0.029779133 |
| ENSG00000254835 | RNF185-AS1 | -0.15368537 | 0.001337459 | 0.029792608 |
| ENSG00000180626 | ZNF594 | 0.153709982 | 0.001335044 | 0.029796223 |
| ENSG00000117298 | ECE1 | -0.153622844 | 0.001343614 | 0.02981482 |
| ENSG00000156802 | ATAD2 | 0.153714194 | 0.00133463 | 0.029815763 |
| ENSG00000181260 | MTHFD2P7 | 0.153633931 | 0.001342521 | 0.029819184 |
| ENSG00000239344 | RP11-771F20.1 | -0.15358663 | 0.001347191 | 0.029865532 |
| ENSG00000153487 | ING1 | 0.153531125 | 0.00135269 | 0.029958715 |
| ENSG00000230067 | HSPD1P6 | 0.153417824 | 0.001363979 | 0.03017983 |
| ENSG00000111679 | PTPN6 | 0.153374662 | 0.001368303 | 0.03024656 |
| ENSG00000196220 | SRGAP3 | 0.153346725 | 0.001371108 | 0.030279617 |
| ENSG00000160883 | HK3 | 0.153322853 | 0.001373509 | 0.030303698 |
| ENSG00000255990 | AC034102.1 | -0.153247319 | 0.001381132 | 0.030413842 |
| ENSG00000119242 | CCDC92 | -0.15324865 | 0.001380997 | 0.03043986 |
| ENSG00000234383 | CTBP2P8 | -0.153222412 | 0.001383654 | 0.030440388 |

| | | | | |
|---|---|---|---|---|
| ENSG00000177098 | SCN4B | 0.153197062 | 0.001386225 | 0.030467961 |
| ENSG00000229292 | RFPL4AL1 | 0.15308317 | 0.001397832 | 0.030635708 |
| ENSG00000183688 | FAM101B | -0.153095808 | 0.00139654 | 0.030636431 |
| ENSG00000130600 | H19 | 0.153106303 | 0.001395467 | 0.030641964 |
| ENSG00000231407 | RP11-576I22.2 | -0.153061663 | 0.001400034 | 0.030654911 |
| ENSG00000073756 | PTGS2 | 0.153011184 | 0.001405214 | 0.030739222 |
| ENSG00000124615 | MOCS1 | 0.152966248 | 0.00140984 | 0.030811267 |
| ENSG00000077348 | EXOSC5 | 0.152874603 | 0.001419319 | 0.030901595 |
| ENSG00000139269 | INHBE | 0.15289744 | 0.001416951 | 0.030908246 |
| ENSG00000164483 | SAMD3 | -0.152882289 | 0.001418521 | 0.030913329 |
| ENSG00000185989 | RASA3 | -0.152903057 | 0.001416369 | 0.030924725 |
| ENSG00000257557 | RP11-84G21.1 | 0.152815476 | 0.001425465 | 0.03100621 |
| ENSG00000151164 | RAD9B | 0.152763667 | 0.001430871 | 0.031094548 |
| ENSG00000226979 | LTA | 0.152713114 | 0.001436163 | 0.031150995 |
| ENSG00000115363 | EVA1A | -0.152685177 | 0.001439096 | 0.031156159 |
| ENSG00000110237 | ARHGEF17 | -0.152659531 | 0.001441793 | 0.031156204 |
| ENSG00000181852 | RNF41 | -0.152721318 | 0.001435303 | 0.031161574 |
| ENSG00000247157 | RP11-434C1.1 | -0.15268939 | 0.001438653 | 0.031175759 |
| ENSG00000003147 | ICA1 | -0.152661453 | 0.001441591 | 0.03118098 |
| ENSG00000096696 | DSP | 0.152617478 | 0.001446225 | 0.031222796 |
| ENSG00000197694 | SPTAN1 | -0.152575651 | 0.001450646 | 0.031289027 |
| ENSG00000137166 | FOXP4 | -0.152558616 | 0.00145245 | 0.031298741 |
| ENSG00000140612 | SEC11A | -0.152527162 | 0.001455787 | 0.031312286 |
| ENSG00000111450 | STX2 | -0.152539579 | 0.001454469 | 0.031313065 |
| ENSG00000090661 | CERS4 | -0.15249893 | 0.001458788 | 0.031347673 |
| ENSG00000257951 | RP11-554D14.4 | 0.152449855 | 0.001464018 | 0.031430849 |
| ENSG00000070731 | ST6GALNAC2 | 0.152432782 | 0.001465841 | 0.031440794 |
| ENSG00000197019 | SERTAD1 | 0.152376317 | 0.001471886 | 0.031541194 |
| ENSG00000116954 | RRAGC | -0.152269082 | 0.001483431 | 0.031759159 |
| ENSG00000225614 | ZNF469 | -0.152205516 | 0.001490313 | 0.031876982 |
| ENSG00000049759 | NEDD4L | -0.15216191 | 0.001495051 | 0.03194877 |
| ENSG00000099365 | STX1B | 0.152072704 | 0.001504787 | 0.032127133 |
| ENSG00000244649 | CTD-2377D24.6 | 0.15203272 | 0.00150917 | 0.032190986 |
| ENSG00000147509 | RGS20 | 0.151981501 | 0.001514801 | 0.032281317 |
| ENSG00000224769 | AC069213.1 | 0.15196635 | 0.001516471 | 0.032287148 |
| ENSG00000090530 | LEPREL1 | -0.151837381 | 0.001530751 | 0.0325612 |
| ENSG00000101298 | SNPH | -0.151791041 | 0.001535912 | 0.03261098 |
| ENSG00000226666 | HSPA9P1 | 0.151801684 | 0.001534725 | 0.032615727 |
| ENSG00000240344 | PPIL3 | 0.151698287 | 0.00154629 | 0.032771143 |
| ENSG00000151552 | QDPR | 0.151710334 | 0.001544939 | 0.03277255 |
| ENSG00000182704 | TSKU | 0.151618393 | 0.001555281 | 0.032811321 |
| ENSG00000168286 | THAP11 | -0.151623936 | 0.001554656 | 0.032828088 |
| ENSG00000005102 | MEOX1 | -0.151661703 | 0.001550401 | 0.032828179 |
| ENSG00000008226 | DLEC1 | -0.151639456 | 0.001552906 | 0.032851137 |
| ENSG00000023041 | ZDHHC6 | -0.151624231 | 0.001554622 | 0.032857376 |
| ENSG00000130720 | FIBCD1 | 0.151585356 | 0.001559013 | 0.032860072 |
| ENSG00000137185 | ZSCAN9 | -0.151467473 | 0.001572396 | 0.033111967 |
| ENSG00000236911 | RP11-78B10.2 | -0.151406647 | 0.001579342 | 0.033137519 |
| ENSG00000134297 | PLEKHA8P1 | -0.151413225 | 0.00157859 | 0.033151824 |
| ENSG00000243926 | TIPARP-AS1 | -0.151413964 | 0.001578505 | 0.033180175 |
| ENSG00000213799 | ZNF845 | 0.151425198 | 0.001577221 | 0.033183352 |
| ENSG00000169570 | DTWD2 | 0.151289577 | 0.001592792 | 0.033389426 |
| ENSG00000230903 | RPL9P8 | -0.151268661 | 0.001595205 | 0.033409719 |
| ENSG00000167895 | TMC8 | 0.151254175 | 0.001596879 | 0.033414512 |
| ENSG00000181577 | C6orf223 | 0.151147527 | 0.001609252 | 0.033551961 |
| ENSG00000017373 | SRCIN1 | 0.151183889 | 0.001605023 | 0.033554559 |
| ENSG00000228797 | FAM207BP | -0.15114834 | 0.001609157 | 0.03358026 |
| ENSG00000122223 | CD244 | -0.151158169 | 0.001608013 | 0.0335867 |
| ENSG00000114626 | ABTB1 | -0.15111619 | 0.001612904 | 0.033597807 |
| ENSG00000184697 | CLDN6 | 0.151088992 | 0.00161608 | 0.033603419 |
| ENSG00000197771 | MCMBP | -0.151063346 | 0.001619079 | 0.033605336 |
| ENSG00000105278 | ZFR2 | 0.151096456 | 0.001615207 | 0.033615496 |
| ENSG00000128626 | MRPS12 | -0.151067337 | 0.001618612 | 0.033625828 |
| ENSG00000138041 | SMEK2 | 0.150958545 | 0.001631392 | 0.033830534 |
| ENSG00000216306 | KRT19P2 | 0.150923364 | 0.001635544 | 0.033886244 |
| ENSG00000151948 | GLT1D1 | -0.150834306 | 0.001646099 | 0.034074397 |
| ENSG00000224137 | AC079767.4 | 0.15082115 | 0.001647663 | 0.034076265 |
| ENSG00000237186 | RP11-229A12.2 | -0.150797573 | 0.00165047 | 0.034103814 |
| ENSG00000241319 | SETP6 | 0.150625295 | 0.001671115 | 0.034438076 |
| ENSG00000196329 | GIMAP5 | -0.150646137 | 0.001668605 | 0.034447754 |
| ENSG00000241537 | RP11-624D20.1 | 0.150628177 | 0.001670768 | 0.034461639 |
| ENSG00000149591 | TAGLN | -0.150573411 | 0.001677379 | 0.034536382 |
| ENSG00000092330 | TINF2 | -0.150538231 | 0.001681638 | 0.034593269 |
| ENSG00000229133 | RPS7P4 | -0.150513921 | 0.001684587 | 0.034623129 |
| ENSG00000213626 | LBH | -0.150418575 | 0.0016962 | 0.034769093 |
| ENSG00000097033 | SH3GLB1 | -0.150426409 | 0.001695243 | 0.034780309 |
| ENSG00000135862 | LAMC1 | -0.150400467 | 0.001698413 | 0.034783619 |
| ENSG00000175779 | C15orf53 | -0.150428183 | 0.001695026 | 0.034806742 |
| ENSG00000181274 | FRAT2 | -0.150343706 | 0.00170537 | 0.03489519 |
| ENSG00000182768 | NGRN | -0.150324342 | 0.001707749 | 0.034912973 |
| ENSG00000253667 | RP11-30L15.4 | 0.150259525 | 0.001715734 | 0.035045231 |
| ENSG00000205955 | HSP90AA5P | 0.150241196 | 0.001717999 | 0.035060523 |
| ENSG00000143318 | CASQ1 | -0.15018643 | 0.001724781 | 0.035167889 |
| ENSG00000163041 | H3F3A | -0.150097593 | 0.001735834 | 0.035330945 |
| ENSG00000183077 | AFMID | 0.150085325 | 0.001737366 | 0.035331026 |
| ENSG00000186628 | FSD2 | 0.150107645 | 0.001734581 | 0.035336548 |
| ENSG00000228991 | RP11-318K12.1 | 0.149983036 | 0.001750184 | 0.035560417 |
| ENSG00000165272 | AQP3 | 0.149939209 | 0.001755703 | 0.035641233 |
| ENSG00000173275 | ZNF449 | -0.149899816 | 0.001760677 | 0.035679556 |
| ENSG00000133816 | MICAL2 | 0.149845642 | 0.001767538 | 0.035693462 |
| ENSG00000237604 | AP001056.1 | -0.149902699 | 0.001760312 | 0.035703451 |
| ENSG00000136010 | ALDH1L2 | 0.149850372 | 0.001766938 | 0.035712535 |
| ENSG00000165905 | GYLTL1B | -0.14982443 | 0.001770231 | 0.03571665 |
| ENSG00000261602 | CTD-2033A16.1 | 0.149870844 | 0.001764343 | 0.035722538 |
| ENSG00000241351 | IGKV3-11 | -0.14985318 | 0.001766582 | 0.035736578 |
| ENSG00000168952 | STXBP6 | -0.149730494 | 0.001782203 | 0.035926851 |
| ENSG00000130176 | CNN1 | -0.149643948 | 0.001793299 | 0.036119041 |
| ENSG00000196796 | CTB-134H23.2 | -0.149537668 | 0.001807011 | 0.036363541 |
| ENSG00000186788 | SPATA31D3 | -0.149504853 | 0.001811264 | 0.036417431 |
| ENSG00000110047 | EHD1 | -0.149479577 | 0.001814546 | 0.036451723 |
| ENSG00000030582 | GRN | 0.149458587 | 0.001817276 | 0.036474875 |
| ENSG00000112977 | DAP | -0.14941742 | 0.001822641 | 0.036550828 |
| ENSG00000143341 | HMCN1 | 0.149377954 | 0.001827798 | 0.036590775 |
| ENSG00000160877 | NACC1 | 0.149380762 | 0.00182743 | 0.03661511 |
| ENSG00000136859 | ANGPTL2 | -0.149314467 | 0.001836121 | 0.036725597 |
| ENSG00000080709 | KCNN2 | -0.149283943 | 0.001840135 | 0.036774072 |
| ENSG00000071564 | TCF3 | -0.149242185 | 0.00184564 | 0.036852235 |
| ENSG00000086717 | PPEF1 | -0.149203605 | 0.001850739 | 0.036890334 |
| ENSG00000196998 | WDR45 | -0.149184537 | 0.001853264 | 0.036908846 |
| ENSG00000235366 | LINC01055 | 0.14920767 | 0.001850201 | 0.03691143 |
| ENSG00000213480 | RP11-364P2.2 | -0.149153274 | 0.001857411 | 0.036959602 |
| ENSG00000154556 | SORBS2 | 0.149136497 | 0.00185964 | 0.036972138 |
| ENSG00000263264 | CTB-133G6.1 | -0.149085353 | 0.001866449 | 0.03707563 |
| ENSG00000163251 | FZD5 | 0.148975304 | 0.001881178 | 0.037336135 |
| ENSG00000142541 | RPL13A | -0.1489215 | 0.001888419 | 0.037383582 |
| ENSG00000121753 | BAI2 | -0.14893229 | 0.001886965 | 0.037386808 |
| ENSG00000254419 | RP11-261P9.4 | -0.148942933 | 0.001885531 | 0.037390435 |
| ENSG00000091039 | OSBPL8 | -0.148882846 | 0.001893636 | 0.037390821 |
| ENSG00000219133 | RP11-203F10.6 | -0.148905757 | 0.001890542 | 0.037393595 |
| ENSG00000126856 | PRDM7 | 0.148887502 | 0.001893007 | 0.037410349 |
| ENSG00000130313 | PGLS | -0.148760381 | 0.001910252 | 0.037686729 |
| ENSG00000105373 | GLTSCR2 | -0.148705985 | 0.001917676 | 0.0377366 |
| ENSG00000232004 | CAP1P2 | -0.14868056 | 0.001921154 | 0.037740801 |
| ENSG00000224531 | SMIM13 | -0.148691942 | 0.001919596 | 0.037742261 |
| ENSG00000133275 | CSNK1G2 | -0.148714114 | 0.001916564 | 0.037746842 |
| ENSG00000114125 | RNF7 | 0.148724979 | 0.00191508 | 0.03774977 |
| ENSG00000225739 | NPM1P18 | 0.148604805 | 0.001931554 | 0.037912896 |
| ENSG00000069974 | RAB27A | -0.148474136 | 0.001949613 | 0.038234904 |
| ENSG00000257950 | P2RX5-TAX1BP3 | -0.1484275 | 0.001956095 | 0.038329516 |
| ENSG00000048392 | RRM2B | 0.148401559 | 0.00195971 | 0.038367836 |
| ENSG00000050165 | DKK3 | -0.148389659 | 0.00196137 | 0.038367849 |
| ENSG00000211664 | IGLV2-18 | 0.148352632 | 0.001966543 | 0.038436523 |

| | | | | |
|---|---|---|---|---|
| ENSG00000156973 | PDE6D | -0.148285597 | 0.001975942 | 0.038555047 |
| ENSG00000180979 | LRRC57 | -0.148288258 | 0.001975568 | 0.038580307 |
| ENSG00000162892 | IL24 | 0.148203264 | 0.001987541 | 0.03874867 |
| ENSG00000182798 | MAGEB17 | 0.148172814 | 0.001991847 | 0.038767244 |
| ENSG00000001461 | NIPAL3 | -0.148160619 | 0.001993573 | 0.038768204 |
| ENSG00000257122 | RRN3P3 | -0.148181831 | 0.001990571 | 0.038775049 |
| ENSG00000164070 | HSPA4L | 0.148098093 | 0.002002448 | 0.038908069 |
| ENSG00000164494 | PDSS2 | 0.148015174 | 0.002014274 | 0.03910499 |
| ENSG00000237592 | IGKV1OR10-1 | -0.147955082 | 0.002022883 | 0.03914067 |
| ENSG00000147224 | PRPS1 | -0.147960625 | 0.002022088 | 0.039158056 |
| ENSG00000135503 | ACVR1B | 0.147967351 | 0.002021123 | 0.039172176 |
| ENSG00000138621 | PPCDC | -0.147976441 | 0.002019819 | 0.039179744 |
| ENSG00000260942 | CAPN10-AS1 | 0.147894921 | 0.002031537 | 0.03927525 |
| ENSG00000150893 | FREM2 | 0.14786824 | 0.002035385 | 0.03928395 |
| ENSG00000124535 | WRNIP1 | -0.147873636 | 0.002034606 | 0.039301721 |
| ENSG00000136872 | ALDOB | 0.147844886 | 0.002038759 | 0.039316252 |
| ENSG00000258867 | LINC01146 | -0.147811633 | 0.002043572 | 0.039376226 |
| ENSG00000092607 | TBX15 | 0.147709191 | 0.002058465 | 0.039630165 |
| ENSG00000239557 | RP11-168J18.6 | -0.147638609 | 0.002068784 | 0.039795694 |
| ENSG00000162552 | WNT4 | -0.147619689 | 0.002071558 | 0.03981593 |
| ENSG00000163554 | SPTA1 | 0.147534399 | 0.002084106 | 0.039990622 |
| ENSG00000164458 | T | -0.147541125 | 0.002083114 | 0.040004786 |
| ENSG00000133138 | TBC1D8B | 0.147438325 | 0.002098323 | 0.040163431 |
| ENSG00000164362 | TERT | -0.14744571 | 0.002097227 | 0.040175711 |
| ENSG00000131467 | PSME3 | -0.147457166 | 0.002095528 | 0.04017645 |
| ENSG00000140853 | NLRC5 | -0.147309276 | 0.00211756 | 0.040498116 |
| ENSG00000250685 | RP11-486L19.2 | 0.147293534 | 0.002119917 | 0.040509687 |
| ENSG00000174276 | ZNHIT2 | -0.147248672 | 0.002126649 | 0.040604771 |
| ENSG00000211671 | IGLV2-8 | 0.147221843 | 0.002130684 | 0.040614737 |
| ENSG00000142546 | NOSIP | -0.14720174 | 0.002133712 | 0.040638953 |
| ENSG00000131469 | RPL27 | -0.147221991 | 0.002130662 | 0.040647827 |
| ENSG00000189357 | SPATA31D4 | -0.147122511 | 0.002145685 | 0.040833357 |
| ENSG00000179934 | CCR8 | -0.147045795 | 0.002157336 | 0.040987611 |
| ENSG00000103942 | HOMER2 | 0.147047938 | 0.00215701 | 0.041015119 |
| ENSG00000105357 | MYH14 | 0.147005811 | 0.002163432 | 0.041069684 |
| ENSG00000123684 | LPGAT1 | -0.146978909 | 0.002167542 | 0.041113951 |
| ENSG00000219395 | HSPA8P15 | 0.146965679 | 0.002169566 | 0.041118611 |
| ENSG00000062282 | DGAT2 | 0.146915718 | 0.002177224 | 0.041229954 |
| ENSG00000197381 | ADARB1 | -0.146823185 | 0.002191474 | 0.041431939 |
| ENSG00000236157 | RP11-187C18.4 | -0.146830945 | 0.002190275 | 0.041443158 |
| ENSG00000023445 | BIRC3 | 0.146747947 | 0.002203122 | 0.041618127 |
| ENSG00000203710 | CR1 | -0.146695251 | 0.002211315 | 0.041738796 |
| ENSG00000198858 | R3HDM4 | -0.146629103 | 0.002221638 | 0.0418312 |
| ENSG00000213343 | RP11-118B13.1 | -0.146636938 | 0.002220413 | 0.041842208 |
| ENSG00000150455 | TIRAP | -0.146601684 | 0.00222593 | 0.041843865 |
| ENSG00000198162 | MAN1A2 | -0.146647506 | 0.002218761 | 0.041845181 |
| ENSG00000229153 | EPHA1-AS1 | 0.146603088 | 0.00222571 | 0.041873773 |
| ENSG00000241313 | WWTR1-AS1 | 0.146525041 | 0.002237967 | 0.04196778 |
| ENSG00000122224 | LY9 | 0.146526963 | 0.002237664 | 0.041996158 |
| ENSG00000102178 | UBL4A | -0.146534945 | 0.002236408 | 0.042006682 |
| ENSG00000248734 | CTD-2260A17.1 | -0.146458007 | 0.002248544 | 0.042097842 |
| ENSG00000187122 | SLIT1 | -0.146459189 | 0.002248357 | 0.042128453 |
| ENSG00000157551 | KCNJ15 | 0.146405902 | 0.002256796 | 0.042218153 |
| ENSG00000067836 | ROGDI | -0.146312483 | 0.002271662 | 0.042461899 |
| ENSG00000241978 | AKAP2 | -0.146230149 | 0.002284837 | 0.042502012 |
| ENSG00000247373 | RP11-486O12.2 | 0.146287428 | 0.002275664 | 0.042502345 |
| ENSG00000159733 | ZFYVE28 | 0.146233327 | 0.002284328 | 0.042526757 |
| ENSG00000044574 | HSPA5 | 0.146238723 | 0.002283462 | 0.04254489 |
| ENSG00000188015 | S100A3 | 0.146192604 | 0.002290869 | 0.042545761 |
| ENSG00000120899 | PTK2B | -0.146175093 | 0.002293687 | 0.042563909 |
| ENSG00000105127 | AKAP8 | -0.146242935 | 0.002282787 | 0.042566614 |
| ENSG00000204449 | TRIM49C | 0.146193713 | 0.00229069 | 0.042576635 |
| ENSG00000254726 | MEX3A | -0.146247 | 0.002282135 | 0.042588802 |
| ENSG00000260907 | AC008088.4 | -0.146065039 | 0.00231147 | 0.04285951 |
| ENSG00000236801 | RPL24P8 | -0.145966447 | 0.002327508 | 0.043018897 |
| ENSG00000100302 | RASD2 | 0.145969181 | 0.002327062 | 0.043045062 |
| ENSG00000262050 | RP11-74E22.3 | 0.145982411 | 0.002324904 | 0.043074063 |
| ENSG00000117505 | DR1 | -0.14596992 | 0.002326941 | 0.043077286 |
| ENSG00000137507 | LRRC32 | -0.145934297 | 0.002332759 | 0.043081512 |
| ENSG00000147526 | TACC1 | -0.145885745 | 0.002340711 | 0.043193871 |
| ENSG00000151694 | ADAM17 | -0.145841986 | 0.002347899 | 0.043291962 |
| ENSG00000260690 | CYCSP39 | -0.145830235 | 0.002349832 | 0.04329308 |
| ENSG00000179101 | RP11-349N19.2 | -0.145779386 | 0.002358216 | 0.043412954 |
| ENSG00000141030 | COPS3 | -0.145751597 | 0.002362809 | 0.043428354 |
| ENSG00000067955 | CBFB | -0.145752484 | 0.002362663 | 0.043460218 |
| ENSG00000214215 | C12orf74 | -0.145704739 | 0.002370573 | 0.043536449 |
| ENSG00000160285 | LSS | -0.145659656 | 0.002378065 | 0.04363938 |
| ENSG00000174307 | PHLDA3 | 0.1456482 | 0.002379972 | 0.04363974 |
| ENSG00000189014 | FAM35DP | 0.145514649 | 0.002402308 | 0.043979545 |
| ENSG00000180357 | ZNF609 | -0.145503272 | 0.002404219 | 0.043979709 |
| ENSG00000180596 | HIST1H2BC | 0.145479986 | 0.002408136 | 0.04398177 |
| ENSG00000140937 | CDH11 | -0.145522631 | 0.002400968 | 0.043989843 |
| ENSG00000109861 | CTSC | -0.145482499 | 0.002407713 | 0.044008806 |
| ENSG00000131015 | ULBP2 | -0.145366685 | 0.002427278 | 0.044296387 |
| ENSG00000204099 | NEU4 | 0.145326406 | 0.002434116 | 0.044386144 |
| ENSG00000132005 | RFX1 | 0.145270162 | 0.002443694 | 0.044525684 |
| ENSG00000090376 | IRAK3 | -0.145214657 | 0.002453179 | 0.044663311 |
| ENSG00000159231 | CBR3 | -0.14519485 | 0.002456573 | 0.044689914 |
| ENSG00000134531 | EMP1 | 0.145107786 | 0.002471538 | 0.044926809 |
| ENSG00000198908 | BHLHB9 | 0.145096479 | 0.002473488 | 0.044926936 |
| ENSG00000091656 | ZFHX4 | -0.145083545 | 0.00247572 | 0.04493218 |
| ENSG00000106609 | TMEM248 | -0.145040309 | 0.002483194 | 0.04503248 |
| ENSG00000121101 | TEX14 | 0.145015032 | 0.002487573 | 0.04504124 |
| ENSG00000086200 | IPO11 | 0.145024123 | 0.002485998 | 0.045047998 |
| ENSG00000260641 | RP11-1299A16.3 | 0.144989756 | 0.00249196 | 0.045085367 |
| ENSG00000134716 | CYP2J2 | 0.14496751 | 0.002495826 | 0.045120007 |
| ENSG00000100100 | PIK3IP1 | -0.144886876 | 0.002509885 | 0.045303326 |
| ENSG00000110934 | BIN2 | -0.144887468 | 0.002509782 | 0.045336859 |
| ENSG00000236047 | AC073410.1 | 0.144753177 | 0.002533356 | 0.045691308 |
| ENSG00000197442 | MAP3K5 | -0.144740761 | 0.002535546 | 0.045695163 |
| ENSG00000108602 | ALDH3A1 | -0.144559095 | 0.002567783 | 0.045989371 |
| ENSG00000184584 | TMEM173 | -0.144621252 | 0.002556711 | 0.046004881 |
| ENSG00000183405 | RPS7P1 | -0.144587698 | 0.002562682 | 0.046004917 |
| ENSG00000181817 | LSM10 | -0.144609944 | 0.002558722 | 0.046005264 |
| ENSG00000164691 | TAGAP | -0.144627164 | 0.00255566 | 0.046021784 |
| ENSG00000167283 | ATP5L | -0.144559835 | 0.002567651 | 0.046022656 |
| ENSG00000163466 | ARPC2 | -0.144570625 | 0.002565726 | 0.04602383 |
| ENSG00000181061 | HIGD1A | -0.144592502 | 0.002561827 | 0.046025302 |
| ENSG00000218502 | H2AFZP3 | 0.144527906 | 0.002573355 | 0.046053494 |
| ENSG00000048540 | LMO3 | 0.144489622 | 0.002580209 | 0.046104786 |
| ENSG00000037280 | FLT4 | -0.144495387 | 0.002579176 | 0.04612197 |
| ENSG00000070159 | PTPN3 | -0.144433157 | 0.00259035 | 0.046250249 |
| ENSG00000163071 | SPATA18 | 0.144415049 | 0.002593609 | 0.046272706 |
| ENSG00000234996 | RP11-480I12.7 | -0.144375139 | 0.002600807 | 0.04632963 |
| ENSG00000165810 | BTNL9 | -0.144385117 | 0.002599006 | 0.046333243 |
| ENSG00000115944 | COX7A2L | -0.144286307 | 0.002616892 | 0.046580275 |
| ENSG00000206527 | PTPLB | -0.14427322 | 0.002619269 | 0.046586721 |
| ENSG00000165943 | MOAP1 | -0.144249348 | 0.002623611 | 0.046592268 |
| ENSG00000154262 | ABCA6 | 0.144256 | 0.002622401 | 0.046606576 |
| ENSG00000257074 | RPL29P33 | -0.144171597 | 0.002637797 | 0.046808244 |
| ENSG00000228857 | AC104653.1 | 0.144145286 | 0.002642614 | 0.046857761 |
| ENSG00000253490 | AC145110.1 | 0.144114467 | 0.002648266 | 0.046921997 |
| ENSG00000135220 | UGT2A3 | 0.143950096 | 0.002678596 | 0.047423045 |
| ENSG00000136813 | KIAA0368 | 0.143891265 | 0.002689528 | 0.047580158 |
| ENSG00000185130 | HIST1H2BL | 0.143812849 | 0.002704163 | 0.04780249 |
| ENSG00000237686 | RP5-1120P11.1 | 0.143766657 | 0.002712818 | 0.047918852 |
| ENSG00000236609 | ZNF853 | -0.143710782 | 0.00272332 | 0.048067637 |
| ENSG00000203813 | HIST1H3H | 0.143670207 | 0.00273097 | 0.048165895 |
| ENSG00000182013 | PNMAL1 | -0.143651435 | 0.002734516 | 0.048191676 |
| ENSG00000230533 | RP11-95M15.1 | -0.143570506 | 0.002749851 | 0.048351372 |
| ENSG00000118777 | ABCG2 | 0.143577083 | 0.002748602 | 0.048366191 |
| ENSG00000232553 | AC006026.10 | -0.143578936 | 0.00274825 | 0.048396829 |

| | | | | |
|---|---|---|---|---|
| ENSG00000205021 | CCL3L1 | 0.143464152 | 0.002770122 | 0.048670791 |
| ENSG00000147443 | DOK2 | -0.143410643 | 0.002780373 | 0.048739791 |
| ENSG00000101146 | RAE1 | -0.143428233 | 0.002776999 | 0.048754572 |
| ENSG00000008283 | CYB561 | -0.143380341 | 0.002786193 | 0.048767869 |
| ENSG00000081665 | ZNF506 | -0.143411678 | 0.002780174 | 0.04877328 |
| ENSG00000008735 | MAPK8IP2 | 0.143385736 | 0.002785156 | 0.048786649 |
| ENSG00000260837 | RP11-434B12.1 | 0.143291726 | 0.002803277 | 0.049029781 |
| ENSG00000127528 | KLF2 | -0.143264675 | 0.002808511 | 0.049084196 |
| ENSG00000260409 | RP11-403B2.7 | 0.143152483 | 0.002830314 | 0.049427885 |
| ENSG00000142694 | EVA1B | -0.143138367 | 0.002833068 | 0.04943864 |
| ENSG00000197217 | ENTPD4 | -0.143111686 | 0.00283828 | 0.04949224 |
| ENSG00000186583 | SPATC1 | 0.143087666 | 0.00284298 | 0.049499536 |
| ENSG00000107551 | RASSF4 | -0.143095944 | 0.00284136 | 0.04950861 |
| ENSG00000164023 | SGMS2 | 0.142995134 | 0.002861152 | 0.049778447 |
| ENSG00000184588 | PDE4B | -0.142968675 | 0.002866367 | 0.049794243 |
| ENSG00000100902 | PSMA6 | -0.142973553 | 0.002865405 | 0.049814958 |
| ENSG00000166845 | C18orf54 | 0.14291014 | 0.002877936 | 0.049957685 |

**Supplementary Table 3:** LCLeQTLs at FDR less than 5% for MS risk SNPs and genes within 1 mega base window from them. Genotype and gene expression data were obtained from 1000 genome project and GEUVADIS, respectively.

**Supplementary Table 3:** LCLeQTLs at FDR less than 5% for MS risk SNPs and genes within 1 mega base window from them. Genotype and gene expression data were obtained from 1000 genome project and GEUVADIS, respectively.

| SNP (rs ID) | Gene Name | Gene Ensembl ID | beta | pvalue | FDR (Benjamini–Hochberg) |
|---|---|---|---|---|---|
| rs983494 | SLAMF7 | ENSG00000026751 | 18.09256633 | 2.98E-09 | 1.01E-06 |
| rs1177228 | C2orf74 | ENSG00000237651 | 0.961880908 | 2.17E-08 | 6.23E-06 |
| rs7222450 | LRRC37A4P | ENSG00000214425 | 0.061178006 | 1.68E-06 | 0.000338063 |
| rs1177228 | KIAA1841 | ENSG00000162929 | 0.594846461 | 0.000143174 | 0.013966559 |
| rs983494 | RP11-312J18.7 | ENSG00000233691 | 0.034504693 | 0.000328798 | 0.026720966 |
| rs1177228 | AC016747.3 | ENSG00000212978 | -0.857558241 | 0.000362896 | 0.028228106 |
| rs9808753 | IFNGR2 | ENSG00000159128 | 1.938095608 | 0.000377158 | 0.02892432 |
| rs1177228 | AHSA2 | ENSG00000173209 | -2.165983291 | 0.000641504 | 0.041253624 |
| rs4796224 | ZNHIT3 | ENSG00000108278 | 2.802999905 | 2.70E-45 | 1.47E-41 |
| rs4896153 | AHI1 | ENSG00000135541 | -0.99456087 | 1.18E-34 | 3.21E-31 |
| rs2331964 | EAF2 | ENSG00000145088 | 2.944517047 | 2.58E-27 | 4.69E-24 |
| rs3809627 | TBX6 | ENSG00000149922 | 0.110036169 | 5.23E-25 | 7.12E-22 |
| rs11079784 | MRPL45P2 | ENSG00000228782 | -0.942655711 | 4.79E-20 | 5.08E-17 |
| rs9909593 | ORMDL3 | ENSG00000172057 | -3.957369608 | 5.60E-20 | 5.08E-17 |
| rs4728142 | IRF5 | ENSG00000128604 | 9.722027443 | 2.90E-17 | 2.25E-14 |
| rs4796224 | DHRS11 | ENSG00000108272 | 0.363750829 | 8.95E-13 | 6.09E-10 |
| rs1465697 | CCDC155 | ENSG00000161609 | -0.049822661 | 2.70E-11 | 1.63E-08 |
| rs6533052 | RP11-10L12.4 | ENSG00000246560 | 0.069803928 | 5.07E-11 | 2.76E-08 |
| rs9900529 | MRPS7 | ENSG00000125445 | -1.312843243 | 1.22E-10 | 6.04E-08 |
| rs6427540 | SETP9 | ENSG00000235101 | 0.133926177 | 1.87E-10 | 8.48E-08 |
| rs12588969 | TRAF3 | ENSG00000131323 | -2.632515316 | 4.89E-10 | 2.05E-07 |
| rs11079784 | TBKBP1 | ENSG00000198933 | 0.360287076 | 1.16E-09 | 4.53E-07 |
| rs6670198 | TNFRSF14 | ENSG00000157873 | -6.158632027 | 1.59E-09 | 5.76E-07 |
| rs2317231 | FCRL3 | ENSG00000160856 | -0.281171723 | 6.82E-09 | 2.19E-06 |
| rs1077667 | TNFSF14 | ENSG00000125735 | -1.07287952 | 1.19E-08 | 3.60E-06 |
| rs2331964 | IQCB1 | ENSG00000173226 | 1.299188397 | 2.32E-08 | 6.26E-06 |
| rs354033 | ZNF767 | ENSG00000133624 | 0.314029155 | 2.41E-08 | 6.26E-06 |
| rs10951154 | HOTAIRM1 | ENSG00000233429 | 0.14990808 | 1.55E-07 | 3.83E-05 |
| rs2469434 | DOK6 | ENSG00000206052 | 0.037826653 | 1.70E-07 | 4.03E-05 |
| rs10936602 | LRRIQ4 | ENSG00000188306 | -0.015577469 | 2.16E-07 | 4.91E-05 |
| rs701006 | RP11-571M6.7 | ENSG00000257342 | 1.487160403 | 2.60E-07 | 5.66E-05 |
| rs9610458 | LL22NC03-86G7.1 | ENSG00000224086 | -0.025456744 | 8.82E-07 | 0.000184665 |
| rs2286974 | DEXI | ENSG00000182108 | -0.607492729 | 2.09E-06 | 0.000406917 |
| rs2269434 | NR1H3 | ENSG00000025434 | -0.234389378 | 2.32E-06 | 0.000434974 |
| rs1415069 | FAM69A | ENSG00000154511 | 0.372335552 | 4.58E-06 | 0.000830704 |
| rs6533052 | KRT8P46 | ENSG00000248971 | -0.025986363 | 1.14E-05 | 0.002000581 |
| rs7975763 | CDK2AP1 | ENSG00000111328 | -3.364132145 | 1.20E-05 | 0.002034026 |
| rs4940730 | MALT1 | ENSG00000172175 | 1.029266898 | 1.32E-05 | 0.00217588 |
| rs6498163 | DEXI | ENSG00000182108 | -0.595181357 | 1.58E-05 | 0.002527138 |
| rs7260482 | PVR | ENSG00000073008 | 0.121678911 | 1.75E-05 | 0.002680742 |
| rs735542 | PRNCR1 | ENSG00000224722 | -0.674626004 | 1.77E-05 | 0.002680742 |
| rs6533052 | SLC9B1 | ENSG00000164037 | 0.048730701 | 1.90E-05 | 0.00280094 |
| rs3923387 | PLEC | ENSG00000178209 | -0.572605153 | 2.41E-05 | 0.003384327 |
| rs883871 | THRA | ENSG00000126351 | -0.096603375 | 2.42E-05 | 0.003384327 |
| rs9308424 | RPL21P28 | ENSG00000220749 | -16.71910179 | 2.51E-05 | 0.003420524 |
| rs1465697 | CD37 | ENSG00000104894 | -7.778334997 | 3.00E-05 | 0.003914524 |
| rs116899835 | RP11-1152H15.1 | ENSG00000258807 | -0.148505541 | 3.02E-05 | 0.003914524 |
| rs6533052 | CISD2 | ENSG00000145354 | 1.278931444 | 3.48E-05 | 0.004403914 |
| rs531612 | SNX32 | ENSG00000172803 | -0.151967697 | 4.03E-05 | 0.004981769 |
| rs34947566 | RMI2 | ENSG00000175643 | 2.539457914 | 4.22E-05 | 0.005100179 |
| rs28703878 | RP11-578O24.2 | ENSG00000254352 | 0.428344486 | 4.89E-05 | 0.005786755 |
| rs5756405 | CSF2RB | ENSG00000100368 | -0.847568318 | 6.08E-05 | 0.007048838 |
| rs1465697 | TEAD2 | ENSG00000074219 | -0.031388956 | 6.42E-05 | 0.007287394 |
| rs35540610 | SP140 | ENSG00000079263 | -3.785997749 | 7.58E-05 | 0.008425714 |
| rs7977720 | RP11-75L1.1 | ENSG00000256582 | 0.034200105 | 8.35E-05 | 0.00903335 |
| rs2317231 | RP11-367J7.3 | ENSG00000227217 | -0.036476045 | 8.46E-05 | 0.00903335 |
| rs5756405 | NCF4 | ENSG00000100365 | -1.58823283 | 9.28E-05 | 0.009608433 |
| rs61863928 | ADO | ENSG00000181915 | 0.553332437 | 9.35E-05 | 0.009608433 |
| rs1465697 | CTC-301O7.4 | ENSG00000197813 | -0.044499733 | 0.00011624 | 0.011720838 |
| rs4808760 | MPV17L2 | ENSG00000254858 | -0.524608154 | 0.000143641 | 0.013966559 |
| rs12609500 | SMARCA4 | ENSG00000127616 | -0.794321769 | 0.000152403 | 0.014558497 |
| rs9308424 | RP11-348H3.2 | ENSG00000236905 | -0.049398885 | 0.00015888 | 0.014823104 |
| rs1112718 | TNKS2-AS1 | ENSG00000228701 | -0.035577099 | 0.000160618 | 0.014823104 |
| rs2469434 | CD226 | ENSG00000150637 | -2.593179179 | 0.000165461 | 0.015015583 |
| rs62013236 | CTSH | ENSG00000103811 | 25.31344443 | 0.000172451 | 0.015393346 |
| rs8062446 | MT1L | ENSG00000260549 | 0.052365547 | 0.000216828 | 0.019042412 |
| rs7977720 | RP11-705C15.3 | ENSG00000257027 | 0.087945341 | 0.000247241 | 0.021368662 |
| rs3809627 | GDPD3 | ENSG00000102886 | -0.080275415 | 0.00028707 | 0.024423368 |
| rs28703878 | ZC2HC1A | ENSG00000104427 | -0.312418538 | 0.000301533 | 0.025259152 |
| rs2150879 | SKA2 | ENSG00000182628 | 0.280357918 | 0.000310082 | 0.025581763 |
| rs2084007 | JADE2 | ENSG00000043143 | 1.301927401 | 0.0003549 | 0.028228106 |
| rs354033 | ZNF777 | ENSG00000196453 | 0.195496787 | 0.000359306 | 0.028228106 |
| rs1465697 | FLT3LG | ENSG00000090554 | -0.18365623 | 0.000398672 | 0.029822173 |

| | | | | | |
|---|---|---|---|---|---|
| rs2327586 | ALDH8A1 | ENSG00000118514 | -0.028233467 | 0.00039982 | 0.029822173 |
| rs7977720 | CLEC2B | ENSG00000110852 | 0.883079238 | 0.000419999 | 0.030903988 |
| rs249677 | NDFIP1 | ENSG00000131507 | 0.147847769 | 0.000432987 | 0.031222914 |
| rs7977720 | RP13-735L24.1 | ENSG00000260423 | -0.013624837 | 0.000435802 | 0.031222914 |
| rs6911131 | FUCA2 | ENSG00000001036 | 1.202890326 | 0.000442849 | 0.031248335 |
| rs1801133 | C1orf167 | ENSG00000215910 | 0.211501279 | 0.000447635 | 0.031248335 |
| rs701006 | TSFM | ENSG00000123297 | 0.925835878 | 0.000507117 | 0.034952592 |
| rs2269434 | ACP2 | ENSG00000134575 | -0.517420771 | 0.000529846 | 0.036062671 |
| rs140522 | ODF3B | ENSG00000177989 | -1.319700393 | 0.000550442 | 0.037001952 |
| rs3809627 | MAPK3 | ENSG00000102882 | -3.524876889 | 0.000574078 | 0.038120185 |
| rs3923387 | PARP10 | ENSG00000178685 | -2.725841428 | 0.000640894 | 0.041253624 |
| rs34947566 | CTD-3088G3.4 | ENSG00000262636 | -0.027827702 | 0.000643996 | 0.041253624 |
| rs3737798 | PEA15 | ENSG00000162734 | 2.788264603 | 0.000661998 | 0.041913728 |
| rs6670198 | TTC34 | ENSG00000215912 | -0.005522123 | 0.000711808 | 0.044083252 |
| rs244656 | TCF7 | ENSG00000081059 | 2.820373802 | 0.000712636 | 0.044083252 |
| rs2150879 | HEATR6 | ENSG00000068097 | 0.155499417 | 0.000720553 | 0.044083252 |
| rs1465697 | ALDH16A1 | ENSG00000161618 | -0.332351249 | 0.000730181 | 0.044175978 |
| rs354033 | KRBA1 | ENSG00000133619 | 0.571862984 | 0.000833244 | 0.049857264 |

**Supplementary Table 4:** MS risk SNPs genotype effect on EBV miRNA expression (EBV mir-QTL) at FDR less than 5%. Genotype data was obtained from 1000 genome project. EBV miRNA expression level were quantified by processing small RNA-seq sequences row available data for 435 LCL samples from GEUVADIS cohort.

**Supplementary Table 4:** MS risk SNPs genotype effect on EBV miRNA expression (EBV mir-QTL) at FDR less than 5%. Genotype data was obtained from 1000 genome project. EBV miRNA expression level were quantified by processing small RNA-seq sequences row data for 435 LCL samples from GEUVADIS cohort.

| SNP (rs ID) | EBV miRNA | beta | pvalue | FDR (Benjamini–Hochberg) |
|---|---|---|---|---|
| rs1365120 | BART3-5p | -5123.69813 | 3.65E-07 | 0.000564806 |
| rs2986736 | BART3-5p | -4469.312619 | 4.43E-07 | 0.000564806 |
| rs11231749 | BART4-3p | 353.2805037 | 1.24E-06 | 0.000769459 |
| rs9308424 | BART3-5p | 4400.366518 | 1.66E-06 | 0.000769459 |
| rs9900529 | BART3-5p | -4184.639928 | 1.67E-06 | 0.000769459 |
| rs6670198 | BART4-3p | 293.0691563 | 1.81E-06 | 0.000769459 |
| rs2986736 | BART4-3p | -293.9680434 | 2.14E-06 | 0.000778424 |
| rs9900529 | BART1-5p | -5947.288375 | 6.83E-06 | 0.00182521 |
| rs9992763 | BART3-3p | -6663.710639 | 7.03E-06 | 0.00182521 |
| rs9992763 | BART2-3p | -311.602437 | 7.35E-06 | 0.00182521 |
| rs7260482 | BART3-5p | -4032.993759 | 7.88E-06 | 0.00182521 |
| rs9900529 | BART2-3p | -304.833919 | 1.65E-05 | 0.003496265 |
| rs2286974 | BART3-5p | 3765.227159 | 1.89E-05 | 0.003590977 |
| rs438613 | BART4-3p | 259.0204214 | 1.97E-05 | 0.003590977 |
| rs2286974 | BART1-5p | 5605.465469 | 2.50E-05 | 0.004078379 |
| rs10801908 | BART4-3p | 268.057404 | 2.56E-05 | 0.004078379 |
| rs1399180 | BART4-3p | 292.3289978 | 3.24E-05 | 0.004860796 |
| rs2286974 | BART2-3p | 293.0153742 | 3.82E-05 | 0.005247463 |
| rs7260482 | BART4-3p | -260.0880207 | 3.91E-05 | 0.005247463 |
| rs10801908 | BART3-5p | 3721.021202 | 4.41E-05 | 0.005622348 |
| rs2986736 | BART2-3p | -290.2534585 | 5.39E-05 | 0.006540584 |
| rs6498163 | BART3-5p | 3818.690629 | 6.01E-05 | 0.006964842 |
| rs11125803 | BHRF1-3 | 3399.467 | 6.35E-05 | 0.007038231 |
| rs2986736 | BART1-5p | -5309.996352 | 7.86E-05 | 0.00812549 |
| rs6672420 | BART4-3p | -255.8323107 | 7.97E-05 | 0.00812549 |
| rs2327586 | BART4-3p | -253.5265849 | 8.67E-05 | 0.008495916 |
| rs12147246 | BART4-3p | -236.3486327 | 9.03E-05 | 0.008521589 |
| rs438613 | BART3-5p | 3377.358054 | 0.000102115 | 0.009077691 |
| rs244656 | BART4-3p | 267.6314559 | 0.000103318 | 0.009077691 |
| rs10951154 | BART3-5p | -3742.508941 | 0.000111586 | 0.009233528 |
| rs10191360 | BART3-5p | -3351.002546 | 0.000112339 | 0.009233528 |
| rs9308424 | BART1-5p | 5368.037803 | 0.000117597 | 0.009363674 |
| rs9992763 | BART3-5p | -3311.570348 | 0.00012456 | 0.009601483 |
| rs3923387 | BART4-3p | 245.5457404 | 0.00012812 | 0.009601483 |
| rs9992763 | BART1-5p | -4974.765469 | 0.000136048 | 0.009658683 |
| rs6498163 | BART1-5p | 5489.230433 | 0.000136465 | 0.009658683 |
| rs6911131 | BART4-3p | -380.6049455 | 0.000158373 | 0.010906356 |
| rs883871 | BART4-3p | -293.1753374 | 0.000169412 | 0.011359548 |
| rs11542663 | BART3-5p | -3666.605064 | 0.000174428 | 0.011395949 |
| rs28834106 | BART4-3p | 241.4335506 | 0.000189894 | 0.012096267 |
| rs3184504 | BART4-3p | 240.1212177 | 0.000206643 | 0.012654902 |
| rs9900529 | BART3-3p | -5615.641121 | 0.000208597 | 0.012654902 |
| rs9900529 | BART4-3p | -226.1263504 | 0.000233215 | 0.013819348 |
| rs1365120 | BART2-3p | -300.830531 | 0.000242036 | 0.014016092 |
| rs2327586 | BART3-5p | -3360.705501 | 0.000278221 | 0.01575349 |
| rs9900529 | BHRF1-3 | 2878.170807 | 0.000316732 | 0.017544213 |
| rs13136820 | BART3-5p | -3463.845382 | 0.000379156 | 0.020296545 |
| rs1365120 | BART1-5p | -5446.72339 | 0.000382352 | 0.020296545 |
| rs6498163 | BART2-3p | 273.094168 | 0.00039283 | 0.020427168 |
| rs6496663 | BART3-5p | -3131.244542 | 0.000404334 | 0.020604859 |
| rs34695601 | BART3-5p | -4259.051882 | 0.000424012 | 0.021183965 |
| rs9909593 | BART3-5p | 3170.372587 | 0.000439627 | 0.02154171 |
| rs35486093 | BART15 | 1765.297758 | 0.000475244 | 0.022847586 |
| rs1365120 | BART3-3p | -6095.335031 | 0.000497613 | 0.023479981 |
| rs9900529 | BART4-5p | -868.3169862 | 0.000513473 | 0.023787805 |
| rs3184504 | BART3-5p | 3173.689431 | 0.000611295 | 0.027813917 |
| rs28834106 | BART3-5p | 3149.781632 | 0.000671272 | 0.029697843 |
| rs1365120 | BHRF1-1 | 26821.18823 | 0.000676011 | 0.029697843 |
| rs7977720 | BART2-3p | -242.9378253 | 0.000744 | 0.032130703 |
| rs11231749 | BART3-5p | 3521.605431 | 0.000786838 | 0.03341437 |
| rs11125803 | BART3-5p | -3126.804771 | 0.000849474 | 0.035482951 |
| rs11542663 | BART1-5p | -4913.081335 | 0.000889053 | 0.036012927 |
| rs11083862 | BART3-5p | -2864.396344 | 0.00089043 | 0.036012927 |
| rs1399180 | BART2-3p | 268.9221799 | 0.000973507 | 0.038757752 |
| rs1250551 | BART2-3p | 268.5963414 | 0.00103544 | 0.040589256 |
| rs11125803 | BART4-3p | -214.5318312 | 0.001069446 | 0.04093438 |
| rs11256593 | BART3-5p | 2918.924567 | 0.001090974 | 0.04093438 |
| rs3737798 | BART3-3p | 5147.294263 | 0.00109244 | 0.04093438 |
| rs7977720 | BART1-5p | -4392.941813 | 0.001114415 | 0.041049931 |
| rs7977720 | BART3-5p | -2905.012276 | 0.001127745 | 0.041049931 |
| rs13136820 | BHRF1-3 | 2881.56052 | 0.001166001 | 0.041844673 |
| rs6670198 | BART3-5p | 2865.255877 | 0.001190175 | 0.042118969 |
| rs5756405 | BART4-3p | -204.5016257 | 0.001235419 | 0.043121212 |
| rs5756405 | BART3-3p | -5034.302005 | 0.001252614 | 0.04313055 |
| rs405343 | BART4-3p | -251.6210633 | 0.001307627 | 0.044424454 |
| rs9308424 | BHRF1-2-3p | 3571.579461 | 0.001389988 | 0.046121347 |
| rs3923387 | BART3-5p | 2936.567399 | 0.001395028 | 0.046121347 |
| rs11256593 | BART4-3p | 199.6158917 | 0.001411878 | 0.046121347 |
| rs10951154 | BART4-3p | -216.1227227 | 0.00146682 | 0.046359422 |
| rs2585447 | BART3-5p | 3417.430657 | 0.001467046 | 0.046359422 |
| rs802730 | BART3-5p | -3287.564208 | 0.001487282 | 0.046359422 |
| rs2150879 | BART3-5p | -2848.636258 | 0.001491944 | 0.046359422 |
| rs61863928 | BART4-3p | -215.4708448 | 0.001539213 | 0.047251976 |
| rs13385171 | BART3-3p | -5122.802493 | 0.001570819 | 0.047648171 |
| rs11083862 | BHRF1-2-3p | -3285.121876 | 0.001610951 | 0.048290629 |
| rs2327586 | BART1-5p | -4395.792305 | 0.00168317 | 0.049868792 |

**Supplementary Table 5:** MS risk SNPs genotype effect on EBV DNA copy number level (DNA-QTL) at FDR less than 5%. Genotype and EBV DNA copy number data were obtained from 1000 genome project and Mandage et al 2017 (PMID: 28654678), respectively.

**Supplementary Table 5:** MS risk SNPs genotype effect on EBV DNA copy number level (DNA-QTL) at FDR less than 5%.
Genotype and EBV DNA copy number data were obtained from 1000 genome project and Mandage et al 2017 (PMID: 28654678), respectively.

| SNP (rs ID) | beta | pvalue | FDR (Benjamini–Hochberg) |
|---|---|---|---|
| rs3184504 | 13.15612091 | 1.08E-06 | 0.000212071 |
| rs9878602 | -10.32325608 | 3.84E-06 | 0.000376694 |
| rs34536443 | -48.75075991 | 1.73E-05 | 0.001132937 |
| rs59655222 | -12.15041248 | 7.35E-05 | 0.00360203 |
| rs72922276 | -16.53754301 | 0.000102685 | 0.003999457 |
| rs9808753 | -9.437956628 | 0.000122432 | 0.003999457 |
| rs11919880 | -9.329281976 | 0.000170468 | 0.004773116 |
| rs983494 | -11.30974637 | 0.000269145 | 0.006594043 |
| rs12211604 | -7.81551953 | 0.000374555 | 0.008156974 |
| rs17724508 | 9.324889179 | 0.000766261 | 0.015018711 |
| rs79979643 | 14.13736931 | 0.001075929 | 0.019171101 |
| rs11749040 | 11.62890843 | 0.001363441 | 0.021348695 |
| rs802730 | -9.014280535 | 0.001524115 | 0.021348695 |
| rs1177228 | 7.277590581 | 0.001633961 | 0.021348695 |
| rs1365120 | -6.971966848 | 0.001681239 | 0.021348695 |
| rs11256593 | 6.951264734 | 0.001742751 | 0.021348695 |
| rs10801908 | 6.227274723 | 0.001987767 | 0.022917788 |
| rs12622670 | -6.528915619 | 0.002721922 | 0.029152675 |
| rs6837324 | 7.78009823 | 0.00283657 | 0.029152675 |
| rs7260482 | -6.383734012 | 0.002974763 | 0.029152675 |
| rs9568402 | -6.585784293 | 0.003319905 | 0.030985776 |
| rs72989863 | -7.379142748 | 0.004807511 | 0.042830556 |
| rs12925972 | 6.171638571 | 0.005087932 | 0.043358025 |
| rs7222450 | 6.23927514 | 0.006052667 | 0.049430116 |

**Supplementary Table 6:** MS risk SNPs genotype effect on LCL intrinsic growth rate (IG-QTL) at FDR less than 5%. Genotype and EBV DNA copy number data were obtained from 1000 genome project and Im et al 2012 (PMID: 22346769), respectively.

**Supplementary Table 6:** MS risk SNPs genotype effect on LCL intrinsic growth rate (IG-QTL) at FDR less than 5%. Genotype and EBV DNA copy number data were obtained from 1000 genome project and Im et al 201[illegible]

| SNP (rs ID) | beta | pvalue | FDR (Benjamini–Hochberg) |
|---|---|---|---|
| rs112741635 | -1157.408598 | 0.001753632 | 0.26853305 |
| rs11852059 | 1110.152511 | 0.002795617 | 0.26853305 |
| rs2327586 | 795.4115565 | 0.004068683 | 0.26853305 |
| rs56095240 | -1715.137568 | 0.007996068 | 0.395805381 |
| rs1077667 | 944.5447227 | 0.011172119 | 0.442415913 |
| rs1076928 | 703.0939845 | 0.017878917 | 0.590004266 |
| rs3184504 | -780.7160103 | 0.029989563 | 0.69170882 |
| rs9843355 | 968.1031779 | 0.031449244 | 0.69170882 |
| rs62420820 | 1164.647277 | 0.033165754 | 0.69170882 |
| rs3923387 | -831.3396817 | 0.035400088 | 0.69170882 |
| rs4796224 | -604.6245139 | 0.040470904 | 0.69170882 |
| rs6589706 | -603.0433706 | 0.043106934 | 0.69170882 |
| rs9955954 | 871.24286 | 0.04813801 | 0.69170882 |
| rs34947566 | 1345.928133 | 0.051782793 | 0.69170882 |
| rs405343 | 757.0986065 | 0.053499329 | 0.69170882 |
| rs962052 | 548.1768875 | 0.063634905 | 0.69170882 |
| rs4896153 | -768.5468928 | 0.06754663 | 0.69170882 |
| rs116877451 | 3324.189294 | 0.070121561 | 0.69170882 |
| rs2150879 | 602.7874332 | 0.072268742 | 0.69170882 |
| rs6837324 | -612.5006095 | 0.072627994 | 0.69170882 |
| rs77654077 | -3287.881768 | 0.073363057 | 0.69170882 |
| rs4325907 | -617.8267054 | 0.079724365 | 0.717519283 |
| rs35703946 | -971.5400282 | 0.091823132 | 0.751322305 |
| rs802730 | 731.9780347 | 0.092720752 | 0.751322305 |
| rs2585447 | -777.3400863 | 0.096438664 | 0.751322305 |
| rs57116599 | 813.5790788 | 0.100130335 | 0.751322305 |
| rs7977720 | 471.8522093 | 0.102453042 | 0.751322305 |
| rs1415069 | 713.3365671 | 0.113753773 | 0.765066131 |
| rs10191360 | 470.2729165 | 0.115463441 | 0.765066131 |
| rs17051321 | -545.8839973 | 0.121104186 | 0.765066131 |
| rs883871 | 465.3373259 | 0.127847439 | 0.765066131 |
| rs6564681 | -525.0576273 | 0.135357207 | 0.765066131 |
| rs9878602 | 426.136772 | 0.138584436 | 0.765066131 |
| rs2364485 | -1204.930836 | 0.140165445 | 0.765066131 |
| rs12609500 | 903.6641105 | 0.146836453 | 0.765066131 |
| rs146566517 | -2621.488103 | 0.153442928 | 0.765066131 |
| rs6020055 | -6862.783028 | 0.154459506 | 0.765066131 |
| rs483180 | 627.4399608 | 0.156199079 | 0.765066131 |
| rs10230723 | 788.6050442 | 0.156954846 | 0.765066131 |
| rs137955 | -468.8128271 | 0.160077086 | 0.765066131 |
| rs1399180 | -414.9053856 | 0.162001164 | 0.765066131 |
| rs9992763 | 519.9332577 | 0.162286755 | 0.765066131 |
| rs1800693 | -410.4182044 | 0.176717326 | 0.803532521 |
| rs10801908 | -441.5863147 | 0.188149933 | 0.803532521 |
| rs7260482 | 354.9535088 | 0.188518894 | 0.803532521 |
| rs13385171 | -455.2533074 | 0.190608821 | 0.803532521 |
| rs438613 | -422.2226063 | 0.19363236 | 0.803532521 |
| rs11899404 | -479.8246722 | 0.195713746 | 0.803532521 |
| rs17780048 | 1290.945646 | 0.198854008 | 0.803532521 |
| rs72922276 | 796.975681 | 0.212623192 | 0.841987842 |
| rs2726479 | 451.1685174 | 0.22685427 | 0.858666065 |
| rs6742 | -424.5186748 | 0.228313447 | 0.858666065 |
| rs2331964 | -347.8131 | 0.233691871 | 0.858666065 |
| rs12588969 | -445.9817435 | 0.236911111 | 0.858666065 |
| rs4262739 | -347.8649195 | 0.241020752 | 0.858666065 |
| rs6032662 | 517.3164721 | 0.242855049 | 0.858666065 |
| rs2836438 | 645.361183 | 0.25243945 | 0.876894931 |
| rs11161550 | -440.6750648 | 0.257710788 | 0.879771312 |
| rs244656 | -382.8359277 | 0.264577542 | 0.880835034 |
| rs11749040 | -564.5521483 | 0.269164058 | 0.880835034 |

| | | | | |
|---|---|---|---|---|
| rs34681760 | 421.3823938 | 0.280692906 | 0.880835034 | |
| rs4409785 | 648.6611453 | 0.281229364 | 0.880835034 | |
| rs12622670 | 390.0624337 | 0.285623728 | 0.880835034 | |
| rs149114341 | -1568.111961 | 0.2884557 | 0.880835034 | |
| rs5756405 | -381.8509247 | 0.289163016 | 0.880835034 | |
| rs34695601 | 662.6505891 | 0.310213021 | 0.922563192 | |
| rs79979643 | -768.5888533 | 0.314069509 | 0.922563192 | |
| rs34536443 | 1716.739985 | 0.318998817 | 0.922563192 | |
| rs35540610 | -459.4367019 | 0.321499294 | 0.922563192 | |
| rs1014486 | 298.7373876 | 0.329916705 | 0.925529073 | |
| rs631204 | 297.6389782 | 0.331881637 | 0.925529073 | |
| rs7731626 | 439.0358746 | 0.352318482 | 0.92886198 | |
| rs6911131 | 361.9758368 | 0.355111694 | 0.92886198 | |
| rs1323292 | -402.2000349 | 0.356656094 | 0.92886198 | |
| rs13136820 | -345.4496561 | 0.357608462 | 0.92886198 | |
| rs2269434 | 287.8689981 | 0.365207798 | 0.92886198 | |
| rs78727559 | -1031.027787 | 0.36578152 | 0.92886198 | |
| rs12614091 | -346.8263785 | 0.376680391 | 0.92886198 | |
| rs4728142 | -302.9603109 | 0.377248614 | 0.92886198 | |
| rs6589939 | -315.6347166 | 0.381169856 | 0.92886198 | |
| rs6496663 | -310.6587308 | 0.383125557 | 0.92886198 | |
| rs111430408 | -1105.544645 | 0.385503392 | 0.92886198 | |
| rs12971909 | -318.5414302 | 0.393190284 | 0.92886198 | |
| rs570429157 | -2063.334791 | 0.394062658 | 0.92886198 | |
| rs4940730 | 260.2012746 | 0.41089583 | 0.95714558 | |
| rs112344141 | 1153.822387 | 0.416243321 | 0.958327646 | |
| rs11919880 | 266.5409525 | 0.424607138 | 0.959604219 | |
| rs9568402 | 270.8771556 | 0.426490764 | 0.959604219 | |
| rs7222450 | -280.4087322 | 0.441305905 | 0.972156047 | |
| rs11079784 | -220.4209367 | 0.450511446 | 0.972156047 | |
| rs2546890 | -227.9432259 | 0.450986006 | 0.972156047 | |
| rs61884005 | 351.5041109 | 0.454889109 | 0.972156047 | |
| rs12211604 | 277.044899 | 0.463535341 | 0.972156047 | |
| rs12147246 | -258.3370089 | 0.46374031 | 0.972156047 | |
| rs10245867 | 200.3930002 | 0.466438507 | 0.972156047 | |
| rs9610458 | -271.1540747 | 0.482871586 | 0.986465769 | |
| rs60600003 | -641.944604 | 0.490071338 | 0.986465769 | |
| rs3737798 | 260.7472191 | 0.49068777 | 0.986465769 | |
| rs10951042 | 242.0031046 | 0.502501645 | 0.986465769 | |
| rs28703878 | -233.0252471 | 0.511433148 | 0.986465769 | |
| rs61863928 | 305.6588251 | 0.513783545 | 0.986465769 | |
| rs6880809 | -209.002685 | 0.521573666 | 0.986465769 | |
| rs71252597 | 354.332817 | 0.5401268 | 0.986465769 | |
| rs10936602 | -178.9955779 | 0.541882501 | 0.986465769 | |
| rs6738544 | -174.197919 | 0.544391947 | 0.986465769 | |
| rs6672420 | 193.2532292 | 0.551352623 | 0.986465769 | |
| rs1738074 | -175.645452 | 0.553870193 | 0.986465769 | |
| rs17741873 | 223.0409499 | 0.568651507 | 0.986465769 | |
| rs9909593 | -169.4391488 | 0.568871293 | 0.986465769 | |
| rs2469434 | 214.9126635 | 0.575926412 | 0.986465769 | |
| rs2289746 | -167.1186809 | 0.579805436 | 0.986465769 | |
| rs11125803 | 197.9904203 | 0.587194342 | 0.986465769 | |
| rs9863496 | 209.5929261 | 0.592769744 | 0.986465769 | |
| rs12434551 | -167.285493 | 0.596371156 | 0.986465769 | |
| rs760517 | -144.5618943 | 0.609288432 | 0.986465769 | |
| rs1250551 | 190.3330647 | 0.612077989 | 0.986465769 | |
| rs735542 | 176.5291045 | 0.61599043 | 0.986465769 | |
| rs61708525 | 196.1230331 | 0.617975451 | 0.986465769 | |
| rs12365699 | 290.2116478 | 0.619330646 | 0.986465769 | |
| rs7975763 | 220.5358257 | 0.629180223 | 0.986465769 | |
| rs3809627 | -182.2038658 | 0.630285747 | 0.986465769 | |
| rs1177228 | -196.2967966 | 0.63039606 | 0.986465769 | |
| rs72989863 | 218.8111377 | 0.634533521 | 0.986465769 | |
| rs138433213 | -1614.605934 | 0.636705957 | 0.986465769 | |
| rs55858457 | -173.6384906 | 0.643465959 | 0.986465769 | |

**Supplementary Table 7:** Processed EBV miRNA expression level for 435 LCL samples. We processed small RNA-seq sequences row available data from GEUVADIS cohort.

**Supplementary Table 7:** Processed EBV miRNA expression level for 435 LCL samples. We processed small RNA-seq sequences available data from the GEUVADIS cohort.

| Samples | ebv-miR-BHRF1-2-3p | ebv-miR-BART15 | ebv-miR-BART1-3p | ebv-miR-BART1-5p | ebv-miR-BART2-3p | ebv-miR-BART2-5p | ebv-miR-BART3-3p | ebv-miR-BART3-5p | ebv-miR-BART4-3p | ebv-miR-BART4-5p | ebv-miR-BHRF1-1 | ebv-miR-BHRF1-2-5p | ebv-miR-BHRF1-3 |
|---|---|---|---|---|---|---|---|---|---|---|---|---|---|
| HG00096 | 30863.3 | 1617.46 | 33256.62 | 25327.11 | 2025.12 | 102242.09 | 28956.54 | 11427.44 | 2025.12 | 7140.51 | 723282.27 | 78.9 | 31757.51 |
| HG00097 | 41622.88 | 1989.19 | 64517.34 | 40534.45 | 1820.3 | 125788.17 | 18428.16 | 14168.29 | 2439.57 | 5254.47 | 654162.29 | 150.13 | 29124.76 |
| HG00099 | 39278.51 | 1958.78 | 32517.7 | 21923.18 | 1132.87 | 63986.39 | 14182.98 | 8972.75 | 1645.5 | 4305.21 | 763934.97 | 71.2 | 46089.95 |
| HG00100 | 42914.44 | 3235.59 | 40670.51 | 37676.48 | 1703.61 | 104536.82 | 33125.04 | 19890.28 | 2498.2 | 5511.31 | 666041.59 | 63.57 | 41891.01 |
| HG00101 | 35540.02 | 2629.86 | 27946.18 | 23056.12 | 1468.07 | 77195.24 | 36173.72 | 13413.32 | 1753.24 | 4985.11 | 732272.24 | 137.3 | 43429.59 |
| HG00102 | 100061.17 | 3134.88 | 21052.1 | 49852.18 | 3797.53 | 116882.45 | 16209.6 | 15266.59 | 2778.06 | 3262.31 | 595346.11 | 229.38 | 72127.64 |
| HG00105 | 97230.73 | 2695.75 | 14704.08 | 40803.82 | 4717.56 | 80014.7 | 21259.65 | 14213.94 | 1776.74 | 3063.35 | 670322.26 | 122.53 | 49074.87 |
| HG00106 | 53926.82 | 5483.14 | 14547.87 | 41834.36 | 1753.87 | 90721.12 | 21138.72 | 19366.39 | 2086.18 | 5796.99 | 684543.81 | 147.69 | 58616.11 |
| HG00107 | 35947.37 | 6897.06 | 37123.36 | 48099.33 | 2722.8 | 136352.08 | 43342.37 | 35068.02 | 2839.34 | 9662.25 | 606856.8 | 392 | 34697.21 |
| HG00108 | 37900.61 | 4940.39 | 66321.51 | 45903.67 | 3190.29 | 116946.8 | 39905.93 | 34455.1 | 3372.59 | 8021.29 | 596693.04 | 182.3 | 42166.48 |
| HG00109 | 38011.26 | 4696.64 | 36107.95 | 45364.3 | 2423.63 | 89852.25 | 30849.91 | 27125.47 | 3094.58 | 7284.58 | 669441.74 | 150.62 | 45583.38 |
| HG00110 | 11061.95 | 1146.18 | 27321.68 | 25189.25 | 1439.39 | 78864.13 | 28023.6 | 6814.87 | 3571.81 | 5775.31 | 793767.99 | 17.77 | 17006.08 |
| HG00111 | 43177.89 | 4887.74 | 37478.41 | 42331.61 | 1934.37 | 101882.56 | 39620.03 | 23454.23 | 2694.3 | 6563.04 | 656183.07 | 397.24 | 39378.24 |
| HG00112 | 13612.56 | 1580.23 | 21972.73 | 14974.57 | 1166.36 | 51447.79 | 28594.65 | 10384.37 | 2791.74 | 4763.27 | 824790.06 | 30.1 | 23891.58 |
| HG00114 | 23559.24 | 2535.18 | 29602.69 | 20460.69 | 1510.86 | 53635.68 | 18194.39 | 19897.31 | 2740.04 | 4353.34 | 784381.76 | 102.43 | 39026.39 |
| HG00115 | 30356.66 | 2640.89 | 19629.73 | 26681.19 | 1402.12 | 53757.15 | 29567.11 | 17186.22 | 3151.37 | 4104.27 | 773366.46 | 231.42 | 37217.53 |
| HG00116 | 25522.98 | 3192.58 | 55432.09 | 49887.7 | 2340.05 | 138521.42 | 41456.47 | 24458.79 | 3692.34 | 7637.49 | 601380.51 | 47.04 | 46418.79 |
| HG00117 | 40029.18 | 4962.15 | 19285.12 | 57916.51 | 2682.01 | 48905.14 | 31576.92 | 13458.09 | 5586.79 | 5901.29 | 663504.17 | 104.83 | 106087.81 |
| HG00118 | 40906.24 | 2620.96 | 18909.49 | 34040.3 | 1913.46 | 76039.94 | 40054.03 | 14970.01 | 1993.86 | 6994.58 | 725924.97 | 112.56 | 35519.61 |
| HG00119 | 41972.51 | 2499.81 | 35175.27 | 30475.25 | 1179.69 | 75219.08 | 23031.98 | 12630.14 | 3436.07 | 3857.39 | 722811.03 | 84.26 | 47627.52 |
| HG00120 | 73425.91 | 3698.77 | 27878.17 | 38911.08 | 1331.56 | 76060.84 | 14633.05 | 16979.12 | 1479.51 | 3586.05 | 688253.4 | 197.27 | 53565.26 |
| HG00121 | 41800.25 | 2100.93 | 19958.8 | 26643.57 | 1459.73 | 65960.9 | 24801.84 | 12114.43 | 1336.95 | 4706.62 | 762035.99 | 136.42 | 36943.56 |
| HG00122 | 22473.98 | 1317.73 | 18124.36 | 18783.22 | 876.62 | 42541.43 | 23529.28 | 11345.87 | 2914.64 | 3361.33 | 823457.81 | 83.75 | 31189.98 |
| HG00123 | 48287.99 | 2540.33 | 23796.6 | 54541.11 | 1997.52 | 95772.63 | 24295.98 | 11941.72 | 1541.57 | 5362.92 | 706819.81 | 108.56 | 22993.25 |
| HG00125 | 81838.49 | 6508.69 | 64406.37 | 54041.32 | 1797.31 | 130906.68 | 34532.7 | 22666.99 | 1779.86 | 6840.23 | 539453.48 | 209.39 | 55018.5 |
| HG00126 | 31809.17 | 933.68 | 28893.01 | 18827.14 | 972.05 | 58259.26 | 14900.56 | 8288.03 | 1394.13 | 1956.9 | 794001.41 | 12.79 | 39751.87 |
| HG00127 | 102068.77 | 2879.51 | 31926.2 | 33939.06 | 1369.86 | 70645.79 | 31562.76 | 13195.42 | 1313.95 | 5255.8 | 664663.13 | 27.96 | 41151.8 |
| HG00128 | 22450.69 | 1273.96 | 24406.33 | 23087.67 | 1262.78 | 73487.18 | 20126.28 | 12583.11 | 1888.58 | 5107 | 777895.74 | 22.35 | 36408.34 |
| HG00129 | 76312.16 | 3241.58 | 15883.74 | 39142.06 | 2512.22 | 103838.57 | 15721.66 | 21178.31 | 2134.04 | 4511.2 | 636159.81 | 54.03 | 79310.62 |
| HG00130 | 20391.52 | 4097.84 | 48880.15 | 43551.97 | 1401.49 | 100280.12 | 32371.41 | 30895.54 | 2485.77 | 7569.31 | 654022.48 | 126.35 | 53909.03 |
| HG00131 | 138780.71 | 2509.89 | 20397.8 | 30437.34 | 1703.14 | 73473.9 | 9481.79 | 9929.98 | 1454.14 | 2599.52 | 621326.06 | 159.36 | 87746.38 |
| HG00132 | 36816.54 | 3205.37 | 73704.52 | 49647.55 | 2214.71 | 157034.67 | 29705.66 | 18360.64 | 3295.87 | 6915.6 | 573109.16 | 61.92 | 45927.8 |
| HG00133 | 30518.64 | 3436.69 | 27493.51 | 34922.7 | 1378.92 | 88636.87 | 25855.78 | 19546.7 | 2719.65 | 5922.98 | 707703.27 | 118.8 | 51737.01 |
| HG00136 | 50699.61 | 2456.09 | 26242.93 | 54406.07 | 2947.31 | 156177.43 | 23965.47 | 15852.93 | 2991.96 | 5388.51 | 608663.29 | 89.31 | 50119.08 |
| HG00137 | 21338.7 | 1716.67 | 22770.11 | 22286.18 | 871.07 | 52615.76 | 12521.01 | 13830.17 | 2358.51 | 3800.11 | 787805 | 117.16 | 57969.54 |
| HG00138 | 30938.98 | 4025.78 | 50804.6 | 36297.26 | 1156.55 | 81952.75 | 27564.07 | 33894.34 | 2003.05 | 6094.04 | 664432.72 | 155.03 | 60678.35 |
| HG00139 | 34018.14 | 1950.19 | 40286 | 22725.6 | 895.1 | 66620.11 | 17746.34 | 9376.93 | 2484.23 | 4423.61 | 756227.87 | 82.16 | 43163.73 |
| HG00141 | 37058.93 | 2514.28 | 36489.12 | 27073.04 | 1217.97 | 75976.87 | 32165.7 | 10748.02 | 2827.68 | 6410.35 | 736905.08 | 99.72 | 30513.26 |
| HG00142 | 32358.43 | 3289.18 | 60227.58 | 77295.76 | 3111.39 | 152369.1 | 41070.32 | 32580.67 | 3066.94 | 11423.24 | 553960.35 | 133.35 | 29024.8 |
| HG00143 | 57519.92 | 2739.04 | 38512.62 | 52954.85 | 4067.07 | 123090.97 | 27722.44 | 17762.28 | 3403.05 | 4814.08 | 640023.24 | 0 | 27390.44 |
| HG00145 | 16584.64 | 3155.56 | 79857.28 | 50264.91 | 2528.04 | 106858.87 | 31752.86 | 30865.36 | 4338.9 | 11958.87 | 635344.11 | 89.65 | 26400.95 |
| HG00146 | 33502.39 | 3411.95 | 40659.69 | 36077.32 | 1071.11 | 85121.48 | 26791.98 | 21471.89 | 2383.4 | 6391.2 | 703500.62 | 241.18 | 39375.78 |
| HG00148 | 70444.38 | 3056.85 | 31081.76 | 37469.2 | 1699.52 | 73444.2 | 32883.93 | 18386.71 | 2543.57 | 5395.11 | 685213.07 | 102.66 | 38279.04 |
| HG00149 | 27280.43 | 2792.88 | 57528.3 | 31444.82 | 1072.27 | 70644.86 | 22530.05 | 16221.14 | 2331.55 | 5411.2 | 701411.4 | 137.15 | 61193.96 |
| HG00150 | 67678.46 | 3794.23 | 17206.39 | 58987.03 | 2647.14 | 120665.31 | 29471.46 | 23603.64 | 1500.04 | 7014.91 | 632312.72 | 176.48 | 34942.2 |
| HG00151 | 31335.09 | 1696.42 | 52027.06 | 46754.12 | 1609.98 | 97225.22 | 16575.18 | 16337.47 | 4041.15 | 5532.26 | 686508.62 | 64.83 | 40281.8 |
| HG00154 | 27762.15 | 4133.96 | 64510.92 | 49874.92 | 1615.7 | 84044.15 | 88924.67 | 38526.04 | 2936.11 | 10619.04 | 599618.92 | 111.43 | 27271.87 |
| HG00155 | 34544.52 | 4257.11 | 52576.3 | 39776.83 | 1575.32 | 104224.3 | 32547.24 | 26471 | 3413.19 | 9011.21 | 648309.81 | 300.06 | 42993.11 |
| HG00157 | 41004.94 | 3102.9 | 70399.04 | 41672.23 | 2101.96 | 120912.85 | 35666.62 | 22287.47 | 3102.9 | 8140.93 | 622981.45 | 0 | 28526.62 |
| HG00158 | 28993.21 | 1881.08 | 39829.88 | 38848.45 | 2658.05 | 103704.92 | 44123.66 | 17134.21 | 2658.05 | 9691.67 | 685981.84 | 81.79 | 24413.18 |
| HG00159 | 34106.46 | 2175.15 | 56268.06 | 34622.6 | 1138.26 | 81024.16 | 17995.64 | 25640.91 | 2645.2 | 5405.6 | 703525.86 | 46.08 | 35406.02 |
| HG00160 | 34538.22 | 10712.82 | 47064.6 | 39480.63 | 1890.5 | 107461.6 | 19993.11 | 15497.68 | 2560.96 | 4674.95 | 677072.95 | 73.28 | 38978.69 |
| HG00171 | 33702.87 | 5958.03 | 30783.14 | 60914.16 | 2178.68 | 87058.34 | 64649.04 | 26425.78 | 3171.69 | 7454.94 | 647216.62 | 251.96 | 30234.76 |
| HG00173 | 37016.56 | 6141.66 | 56691.08 | 67315.92 | 2552.08 | 134412.23 | 33199.8 | 39227.86 | 3127.63 | 8443.84 | 582480.75 | 143.89 | 29246.72 |
| HG00176 | 41117.84 | 28079.91 | 51027.36 | 99846.78 | 2528.36 | 160550.65 | 116630.64 | 72197.98 | 2376.89 | 15228.4 | 353463.09 | 413.63 | 56532.66 |
| HG00177 | 61205.38 | 5244.03 | 49631.04 | 100535.64 | 3108.96 | 200996.37 | 26444.92 | 45660.56 | 2434.73 | 10525.53 | 456418.32 | 224.74 | 37569.76 |
| HG00178 | 52804.39 | 3444.93 | 54096.24 | 91452.26 | 3875.55 | 185434.38 | 23522.45 | 30627.62 | 2960.49 | 11572.83 | 513241.47 | 161.48 | 26805.9 |
| HG00179 | 26851.75 | 4930.67 | 40999.95 | 39539.59 | 1711.6 | 76645.26 | 33886.59 | 29317.09 | 2496.74 | 5527.38 | 709310.18 | 157.03 | 28610.46 |
| HG00180 | 29297.43 | 3078.71 | 57502.41 | 42279.1 | 2142.33 | 116806.65 | 71831.91 | 21437.49 | 2667.27 | 9690.14 | 620452.87 | 42.56 | 22771.13 |
| HG00181 | 41719.12 | 6961.42 | 19378.41 | 47273.45 | 2616.7 | 144214.87 | 44582.69 | 19279.67 | 2715.45 | 14515.29 | 610284.13 | 74.06 | 46360.07 |
| HG00182 | 38157.69 | 4454.96 | 40066.96 | 47081.45 | 2739.38 | 129387.51 | 50554.1 | 23450.79 | 2946.91 | 11580.13 | 621079.43 | 110.68 | 28389.99 |
| HG00183 | 34678.58 | 4661.33 | 80094.09 | 37348.38 | 1284.39 | 92974.09 | 42644.69 | 28025.72 | 2236.86 | 8442.35 | 624705.06 | 331.92 | 42572.54 |
| HG00185 | 19174.07 | 1305.13 | 61147.54 | 31178 | 1933.52 | 144578.9 | 25860.82 | 12213.4 | 2481.35 | 9506.47 | 664905.02 | 0 | 25715.8 |
| HG00186 | 15489.57 | 6985.41 | 48198.96 | 45564.32 | 1623.48 | 94752.28 | 19955.16 | 11312.02 | 2076.69 | 4693.2 | 726189.06 | 22.16 | 23137.68 |
| HG00187 | 42446.12 | 2854.24 | 39842.16 | 35811.09 | 1283.34 | 77559.63 | 20352.41 | 15703.63 | 1964.95 | 3546.5 | 721882.31 | 159.75 | 36593.87 |
| HG00188 | 43914.84 | 4733.34 | 73766.02 | 59828.98 | 2006.31 | 123251.78 | 28935.68 | 71467.53 | 1558.3 | 9369.28 | 539658.73 | 282.44 | 41226.77 |
| HG00189 | 23014.11 | 2330.28 | 62113.34 | 31654.71 | 1629.13 | 86570.98 | 43141.14 | 15590.2 | 3340.76 | 6207.21 | 702177.68 | 20.62 | 22209.85 |
| HG00231 | 76189.59 | 2642.12 | 35922.67 | 76274.27 | 4843.89 | 160203.92 | 17656.47 | 16657.21 | 3031.66 | 4149.48 | 538552.08 | 76.21 | 63791.95 |
| HG00232 | 33551.85 | 3470.22 | 53781.14 | 36049.82 | 1198.45 | 83540.29 | 22438.49 | 24772.82 | 2137 | 6762.35 | 689514.27 | 202.15 | 42504.14 |
| HG00233 | 40054.8 | 3853.07 | 53606.19 | 53229.44 | 1278.65 | 106807.09 | 29117.79 | 41270.66 | 2460.26 | 10954.13 | 621006.36 | 245.45 | 36104.69 |
| HG00234 | 48669.79 | 3607.76 | 79114.59 | 44503.41 | 2606.89 | 138886.95 | 24183.6 | 18341.36 | 3351.72 | 6191.37 | 588692.6 | 139.66 | 41687.03 |
| HG00235 | 44932.96 | 4134.86 | 34436.78 | 46290.86 | 1908.4 | 92924.25 | 53092.58 | 23952.83 | 3327.46 | 11193.48 | 650592.09 | 538.27 | 32675.18 |
| HG00236 | 27685.5 | 3029.07 | 48805.64 | 38346.78 | 1493.1 | 105424.46 | 32036.22 | 20084.25 | 2517.08 | 9918.3 | 674120.27 | 71.44 | 36467.9 |
| HG00237 | 39680.11 | 9002.32 | 46868.85 | 63875.66 | 3801.95 | 164037.35 | 106163.24 | 20677.65 | 1580.5 | 14515.87 | 495651.79 | 72.83 | 34071.87 |
| HG00238 | 26158.32 | 3225.61 | 53152.24 | 30782.59 | 1545.42 | 83201.05 | 51346.25 | 15049.87 | 2800.32 | 7592.32 | 689646.29 | 92.84 | 35406.87 |
| HG00239 | 75182.17 | 2333.18 | 73369.47 | 47650.67 | 2171.65 | 119602.28 | 14806.71 | 17103.99 | 2225.49 | 5456.05 | 604293.05 | 143.58 | 35661.73 |
| HG00240 | 34865.29 | 2795.01 | 65909.12 | 45722.44 | 2072.16 | 117939.6 | 30653.5 | 18692.8 | 2905.84 | 7493.51 | 642938.03 | 77.1 | 27935.6 |
| HG00242 | 46440.91 | 13033.7 | 49991.51 | 33977.4 | 1738.91 | 82533.3 | 12188.51 | 12584.82 | 2608.36 | 4076.32 | 694069.93 | 117.28 | 46630.97 |
| HG00244 | 27648.12 | 4326.12 | 59833.79 | 48099.6 | 1862.18 | 85603.46 | 59199.51 | 23622.88 | 2480.2 | 8270.04 | 644119.9 | 138.24 | 34763.45 |
| HG00245 | 57721.79 | 7424.79 | 15879.48 | 40776.49 | 1975.95 | 101623.87 | 32657.12 | 23783.29 | 1484.96 | 6849.97 | 645945.1 | 239.51 | 63625.69 |
| HG00246 | 50299.99 | 3650.99 | 116413.19 | 96972.23 | 5209.06 | 200618.58 | 25556.95 | 31393.89 | 3185.9 | 6999.67 | 429491.65 | 46.51 | 30161.39 |
| HG00250 | 44377.01 | 2339.93 | 46052.77 | 32687.55 | 1604.23 | 91696.81 | 22040.34 | 18791 | 1808.6 | 7428.52 | 689309.87 | 122.62 | 41740.75 |
| HG00251 | 34785.49 | 4085.91 | 41659.76 | 61537.19 | 2180.99 | 96847.22 | 54966.59 | 38843.8 | 3671.8 | 8144.22 | 620286.01 | 248.47 | 32742.53 |
| HG00252 | 27483.18 | 2559.52 | 76342.16 | 40914.33 | 1153.05 | 110857.69 | 18398.14 | 16725.59 | 3256.42 | 5359.79 | 661813.71 | 12.67 | 35123.73 |
| HG00253 | 28156.99 | 2515.58 | 93067.31 | 47105.68 | 1138.73 | 98661.19 | 24259.23 | 37052.66 | 2225.58 | 7590.65 | 615341.74 | 119.73 | 42758.28 |
| HG00255 | 43352.98 | 3959.07 | 37875.88 | 33565.41 | 1410.27 | 119901.23 | 28027.4 | 26870.13 | 3476.48 | 5781.65 | 639368.61 | 182.73 | 56214.1 |
| HG00256 | 60398.09 | 3607.71 | 25772.34 | 38150.53 | 2135.6 | 99066.97 | 28571.43 | 17271.41 | 2405.14 | 3607.71 | 677130.42 | 248.81 | 40908.15 |
| HG00257 | 51960.01 | 3683.24 | 12891.34 | 44922.39 | 3354.38 | 109839.52 | 42949.22 | 16508.81 | 1907.39 | 8353.06 | 664627.73 | 526.18 | 38476.72 |
| HG00258 | 33282.92 | 3189.58 | 32750.01 | 37530.47 | 1771.12 | 100389.49 | 32538.42 | 20744.03 | 1998.39 | 7915.17 | 694615.33 | 125.39 | 33149.69 |
| HG00259 | 61290.77 | 2364.51 | 45004.12 | 24429.97 | 1658.1 | 71965.39 | 34898.55 | 11194.62 | 2011.3 | 4748.64 | 700934.03 | 176.6 | 39323.42 |
| HG00260 | 34762.31 | 4418.94 | 78098.49 | 41570.21 | 1027.06 | 85709.69 | 27326.58 | 25525.2 | 1782.06 | 7830.13 | 638632.3 | 135.22 | 53162.48 |
| HG00261 | 18602.17 | 1799.75 | 38989.86 | 21127.51 | 960.34 | 54419.35 | 13330.96 | 12555.58 | 1451.18 | 5498.84 | 804780.37 | 64.02 | 26420.06 |
| HG00262 | 42568.93 | 3270.41 | 43934.67 | 36968.73 | 1010.92 | 74436.21 | 20030.86 | 36138.57 | 1703.83 | 5011.06 | 676851.7 | 187.45 | 57813.01 |
| HG00263 | 19204.19 | 1944.52 | 29264.29 | 26098.42 | 1767.75 | 72252.76 | 31064.19 | 15588.34 | 3760.49 | 5640.73 | 763860.77 | 32.14 | 29521.42 |
| HG00264 | 29315.92 | 2497.38 | 34428.15 | 25643.81 | 826.66 | 48168.08 | 17150.99 | 25652.52 | 2358.15 | 3498.07 | 752320.08 | 165.33 | 57970.51 |
| HG00265 | 49670.43 | 3506.43 | 25411.67 | 49948.72 | 2067.28 | 121794.72 | 15965.78 | 27391.49 | 1916.21 | 9167.6 | 635395.05 | 206.73 | 57549.95 |
| HG00266 | 21356.74 | 2868.3 | 43404.64 | 38347.67 | 1532.06 | 66362.56 | 21137.87 | 40352.03 | 3386.67 | 8224.76 | 719954.84 | 161.27 | 32910.58 |
| HG00267 | 47134.33 | 3911.15 | 45554.83 | 80679.94 | 3785.79 | 157849.87 | 35074.96 | 39913.75 | 2732.79 | 12360.23 | 539813.47 | 150.43 | 30587.17 |
| HG00268 | 39678.69 | 4008.58 | 80797.96 | 62555.78 | 2239.17 | 135258.29 | 35998.94 | 36296.45 | 3022.09 | 10820.04 | 558335.81 | 78.29 | 30909.92 |
| HG00269 | 20151.04 | 1872.5 | 66703.04 | 56869.33 | 1562.48 | 110018.48 | 27752.63 | 13405.09 | 3670.59 | 7849.61 | 669994.17 | 49.6 | 19307.8 |
| HG00271 | 37317.25 | 3125.99 | 69931.32 | 61984.58 | 3024.63 | 184311.67 | 26836.47 | 28296.07 | 2680 | 11125.44 | 528219.04 | 89.2 | 43058.36 |
| HG00272 | 20598.09 | 1773.81 | 88237.36 | 40836.74 | 1875.4 | 130699.44 | 46369.16 | 14120.17 | 3453.85 | 8947.2 | 623850.34 | 39.07 | 19199.36 |
| HG00273 | 19033.01 | 7368.34 | 45255.19 | 39398.59 | 2463.58 | 93686.89 | 24788.82 | 11817.71 | 2586.76 | 5307.89 | 728353.17 | 29.86 | 19910.19 |
| HG00274 | 25120.04 | 4165.19 | 59609.75 | 39742.52 | 2094.68 | 102542.62 | 24394.96 | 40741.52 | 3440.11 | 6968.84 | 634881.25 | 201.41 | 56097.13 |
| HG00275 | 77418.21 | 3076.92 | 29814.32 | 62422.63 | 4208.66 | 136657.82 | 21290.89 | 22528.74 | 2157.38 | 8417.33 | 594694.96 | 35.37 | 36887.71 |
| HG00276 | 31316.7 | 2961.97 | 55657.93 | 45887.08 | 1520.03 | 130040.29 | 28052.81 | 27641.57 | 2758.95 | 8302.88 | 627203.26 | 93.7 | 38339.01 |
| HG00277 | 63872.86 | 2136.18 | 36449.25 | 34380.17 | 2259.2 | 74889.28 | 20332.84 | 14394.04 | 2919.07 | 5871.7 | 694425.8 | 212.5 | 47857.11 |
| HG00278 | 23490.25 | 2181.64 | 40623.65 | 45645.18 | 1636.23 | 83842.7 | 17866.88 | 36109.91 | 3084.39 | 7748.58 | 708167.99 | 37.61 | 29564.99 |
| HG00280 | 31159.2 | 2133.78 | 41797.82 | 38120.46 | 1740.31 | 101180.39 | 28919.49 | 16631.36 | 3435.23 | 7687.65 | 691616.22 | 60.53 | 35517.55 |
| HG00281 | 34155.6 | 3671.31 | 67878.06 | 51171.52 | 3341.31 | 161970.96 | 43024.5 | 36878.15 | 2392.54 | 11550.2 | 556121.61 | 82.5 | 27761.74 |
| HG00282 | 19856.81 | 1565.14 | 51483.08 | 41369.14 | 1850.57 | 102457.13 | 25879.5 | 12844.6 | 3867.65 | 5932.3 | 713731.83 | 9.51 | 19152.73 |
| HG00284 | 57311.32 | 6027.88 | 95299.34 | 69723.48 | 2704.02 | 135015.15 | 40816.01 | 74883.59 | 1774.27 | 9367.23 | 451687.88 | 193.7 | 55196.14 |
| HG00285 | 33732.8 | 3153.96 | 48385.16 | 49319.86 | 1603.09 | 86279.73 | 28808.65 | 21132.61 | 2887.65 | 7738.7 | 686718.36 | 140.99 | 30098.43 |
| HG00306 | 36550.34 | 5645.83 | 47999.37 | 55769.76 | 3147.5 | 164437.19 | 19121.06 | 42392.89 | 2498.33 | 13711.3 | 556891.06 | 236.06 | 51599.32 |
| HG00308 | 35862.26 | 5804.41 | 59939.81 | 67854.91 | 2521.11 | 150621.48 | 47803.31 | 43503.75 | 3889.15 | 9888.99 | 539262.82 | 175.89 | 32872.11 |
| HG00309 | 36800.46 | 2192.06 | 53349.79 | 36597.23 | 1451.69 | 98221.67 | 17013.86 | 18610.73 | 2380.78 | 5603.54 | 693387.53 | 116.14 | 34274.52 |
| HG00310 | 20453.12 | 11513.45 | 84249.08 | 89727.45 | 4260.06 | 212849.56 | 126954.54 | 26092.85 | 1976.73 | 19630.15 | 385196.3 | 80.68 | 17016.02 |
| HG00311 | 25762.11 | 3609.98 | 52508.75 | 68261.38 | 3336.49 | 141901.25 | 64687.86 | 26874.27 | 3518.82 | 13619.46 | 570977.98 | 127.63 | 23556.01 |
| HG00313 | 43031.4 | 2894.74 | 49468.02 | 49029.64 | 1280.37 | 95512.46 | 13903.1 | 18273.04 | 2372.85 | 5392.84 | 673235.5 | 167 | 45439.05 |
| HG00315 | 28537.78 | 7705.63 | 73281.49 | 83427.94 | 3533.52 | 171893.63 | 88664.36 | 59232.56 | 4044.39 | 12757.56 | 438440.14 | 283.82 | 28197.2 |
| HG00319 | 55753.95 | 4955.29 | 32583.12 | 75381.34 | 3432.72 | 132048.83 | 43158.1 | 30589.93 | 2934.42 | 7862.03 | 578551.06 | 221.47 | 32527.75 |
| HG00320 | 21437.81 | 2809.09 | 49898.36 | 59175.75 | 4805.03 | 158750.69 | 51820.37 | 26686.38 | 4139.72 | 11864.72 | 586102.38 | 73.92 | 22435.78 |
| HG00321 | 34803.77 | 2709.05 | 65426.66 | 39283.6 | 1637.39 | 115744.25 | 29574.23 | 22606.12 | 3766.92 | 7763.81 | 640656.98 | 68.99 | 35958.22 |
| HG00323 | 27612.5 | 2765.66 | 56095.46 | 42498.57 | 2831.77 | 176021.42 | 40327.91 | 20351.27 | 3283.53 | 10820.22 | 590407.25 | 44.07 | 26940.37 |
| HG00324 | 48708.84 | 4397.9 | 49555.23 | 54650.16 | 2356.61 | 111258.63 | 43497.74 | 33357.67 | 3385.55 | 12247.74 | 604952.2 | 663.83 | 30967.87 |
| HG00325 | 44440.1 | 5046.38 | 93819.97 | 59650.31 | 2896.34 | 140303.49 | 45772.77 | 37137.07 | 2558.73 | 11265.5 | 514268.45 | 248.77 | 42592.13 |
| HG00326 | 16967.36 | 1151.39 | 58060.94 | 27517.76 | 1083.95 | 63128.99 | 30205.95 | 10694.93 | 3786.58 | 5024.69 | 761098.4 | 57.81 | 21221.25 |
| HG00327 | 27460.99 | 3580.64 | 74002.28 | 62654.19 | 3200.02 | 148533.21 | 40380.9 | 25945.56 | 4264.35 | 10847.65 | 570879.79 | 63.44 | 28186.98 |
| HG00328 | 33041.71 | 4471.04 | 71449.43 | 64073.54 | 1520.68 | 148663.05 | 25457.25 | 46394.16 | 2980.69 | 12635.71 | 553464.77 | 94.81 | 35749.36 |
| HG00329 | 48575.99 | 3560.02 | 46712.8 | 53599.95 | 2528.61 | 120142.4 | 29478.31 | 30809.16 | 4059.09 | 9415.76 | 611558.42 | 99.81 | 39459.68 |
| HG00330 | 18998.26 | 1673.55 | 70741.83 | 35323.95 | 1297.86 | 84813.35 | 37381.74 | 14225.21 | 2390.79 | 5037.74 | 710210.39 | 25.62 | 17879.71 |
| HG00331 | 28191.56 | 4992.05 | 67077.5 | 57883.73 | 1510.62 | 99390.75 | 26090.7 | 40786.72 | 2671.1 | 9243.79 | 636941.15 | 100.04 | 25120.3 |
| HG00332 | 24933.62 | 2689.09 | 55279.46 | 31020.95 | 907.71 | 86090.5 | 11022.99 | 28048.2 | 2728.8 | 7636.1 | 688173.69 | 153.18 | 61315.72 |
| HG00334 | 28131.6 | 3833.87 | 57521.6 | 51071.68 | 2016.17 | 118773.34 | 26994.97 | 24568.35 | 2940.81 | 7848.16 | 639444.67 | 112.76 | 36742.02 |
| HG00335 | 26826.38 | 2707.86 | 67648.92 | 41998.01 | 1903.59 | 110427.4 | 45924.17 | 20991.87 | 4026.1 | 10155.67 | 646264.91 | 61.87 | 21058.49 |
| HG00336 | 42734.26 | 3649.36 | 61548.37 | 48882.96 | 2054.68 | 151555.58 | 22724.14 | 30498.18 | 2668.02 | 12926.08 | 587668.86 | 92 | 32997.53 |
| HG00337 | 23057.89 | 6268.36 | 71733.42 | 59660.28 | 2786.5 | 130678.18 | 106461.35 | 47461.16 | 3582.64 | 13741.01 | 507031.75 | 211.63 | 27320.78 |
| HG00338 | 41658.14 | 2717.47 | 86588.94 | 62667.41 | 2220.73 | 121526.46 | 28772.07 | 36758.9 | 3564.85 | 8142.67 | 567805.28 | 146.1 | 36982.93 |
| HG00341 | 16677.1 | 2114.24 | 101348.04 | 95174.47 | 3230.55 | 172166.5 | 27840.27 | 39121.83 | 4160.82 | 11484.53 | 508668.37 | 50.74 | 17962.55 |
| HG00342 | 27130.31 | 1885.89 | 50985.13 | 33350.43 | 1869.34 | 109927.38 | 47759.27 | 15930.78 | 2944.63 | 11364.95 | 678985.59 | 66.17 | 17800.13 |
| HG00343 | 65852.5 | 4047.51 | 55418.62 | 43402.15 | 1750.65 | 135752.5 | 25181.37 | 29340.91 | 1960.73 | 10195.79 | 590487.66 | 112.04 | 36497.58 |
| HG00344 | 33319.99 | 27722.56 | 94694.99 | 101873.27 | 2035.43 | 163065.09 | 128259.23 | 65004.85 | 1940.44 | 20842.8 | 335893.45 | 468.15 | 24879.74 |
| HG00345 | 30915.1 | 1697.57 | 80993.42 | 32957.71 | 1290.43 | 73664.89 | 22606.67 | 15733.58 | 3222.62 | 5023.7 | 694264.83 | 103.51 | 37525.96 |
| HG00346 | 17397.56 | 1946.78 | 63916.96 | 45546.01 | 1946.78 | 144744.78 | 25257.33 | 11353.81 | 5883.93 | 7402.13 | 646070.48 | 21.79 | 28511.66 |
| HG00349 | 50250.54 | 5409.41 | 36869.38 | 83247.92 | 3473.41 | 172873.25 | 37040.2 | 38634.55 | 3160.23 | 15089.4 | 515943.51 | 0 | 37922.79 |
| HG00350 | 30397.07 | 4097.32 | 47705.22 | 48408.42 | 1837.7 | 105442.78 | 29965.78 | 28981.29 | 3150.34 | 8035.25 | 654352.82 | 93.76 | 37494.73 |
| HG00351 | 18260.5 | 3159.66 | 17210.08 | 21411.76 | 1504.2 | 44210.08 | 23294.12 | 18798.32 | 2630.25 | 3504.2 | 807974.79 | 100.84 | 37941.18 |
| HG00353 | 20866.28 | 2738.3 | 63670.81 | 48546.47 | 1507.13 | 99937.38 | 46317.62 | 27584.67 | 3099.16 | 5986.05 | 661151.15 | 63.68 | 18531.29 |
| HG00355 | 48401.96 | 2630.37 | 55880.56 | 43655.51 | 2212.51 | 107652.17 | 27878.67 | 25291.16 | 2619.65 | 7232.17 | 643180.87 | 96.43 | 33267.98 |
| HG00356 | 15371.29 | 1759.53 | 85913.08 | 53357.39 | 1589.83 | 137644.92 | 42442.97 | 15746.41 | 3072.47 | 15290.9 | 610751.86 | 17.86 | 17041.5 |
| HG00358 | 26453.32 | 3503.65 | 71813.91 | 41215.58 | 1986.38 | 114858.28 | 46126.56 | 24862.75 | 3159.15 | 10137.14 | 624350.39 | 109.95 | 31422.94 |
| HG00360 | 59006.65 | 5235.6 | 53346.54 | 82354.61 | 6296.87 | 184165.84 | 42238.57 | 35941.7 | 3466.82 | 7358.14 | 488113.77 | 353.76 | 32121.13 |
| HG00361 | 51826.53 | 2894.9 | 84382.41 | 47859.73 | 2331.57 | 119919.26 | 21547.44 | 22157.72 | 2206.38 | 5742.85 | 596482.31 | 133.01 | 42515.9 |
| HG00362 | 56172.38 | 3309.62 | 45636.27 | 52731.19 | 1578.9 | 101343.08 | 24017.49 | 22630.89 | 3299.49 | 4817.67 | 647956.03 | 172.06 | 36334.93 |
| HG00364 | 34160.89 | 3894.07 | 69098.93 | 50205.15 | 2389.93 | 121744.14 | 60567.06 | 32506.33 | 3593.24 | 10203.14 | 582013.72 | 91.92 | 29514.74 |
| HG00365 | 85166.76 | 4432.12 | 93680.25 | 73305.27 | 3698.74 | 172724.95 | 26975.32 | 39793.38 | 3921.94 | 9565.72 | 448472.67 | 127.54 | 38135.32 |
| HG00366 | 59976.37 | 4886.17 | 48848.26 | 81789.63 | 2684.71 | 157176.22 | 18564.76 | 38887.99 | 2778.67 | 6215.1 | 515960.59 | 107.39 | 62124.14 |
| HG00367 | 26967.48 | 2427.65 | 36933.64 | 65318.23 | 2729.66 | 123756.17 | 23959.05 | 26042.11 | 2826.46 | 8963.35 | 643119.32 | 54.21 | 36902.67 |
| HG00369 | 37496.49 | 2322.79 | 51203.51 | 37215.71 | 2705.67 | 116930.85 | 33948.49 | 21951.66 | 2475.94 | 8678.56 | 649079.82 | 76.58 | 35913.93 |
| HG00371 | 26173.71 | 2385.29 | 82985.48 | 41742.55 | 2530.34 | 142440.41 | 45481.65 | 18324.82 | 2546.46 | 13779.88 | 600931.55 | 48.35 | 20629.52 |
| HG00372 | 36143.82 | 12717.72 | 40323.05 | 47256.01 | 2442.35 | 98160.94 | 102262.33 | 26097.11 | 2534.79 | 14245.4 | 583594.43 | 335.7 | 33886.35 |
| HG00373 | 33689.09 | 3121.86 | 59140.07 | 42277.56 | 1904.56 | 148092.75 | 28995.08 | 20312.28 | 3467.73 | 10789.49 | 608200.37 | 103.31 | 39905.85 |
| HG00375 | 22747.24 | 3866.05 | 38699.6 | 66347.75 | 2596.9 | 133867.03 | 38426.24 | 27199.06 | 2538.32 | 12222.98 | 627491.95 | 78.1 | 23879.72 |
| HG00376 | 38238.09 | 4373.46 | 55343.18 | 49047.56 | 2818.45 | 124098.31 | 53863.76 | 21759.32 | 3455.57 | 7310.7 | 608127.08 | 118.79 | 31445.73 |
| HG00378 | 17857.34 | 2673.94 | 89772.17 | 46136.48 | 2210.39 | 147276.19 | 36406.18 | 23166.87 | 3222.98 | 10247.56 | 588296.02 | 54.79 | 32679.09 |
| HG00379 | 38743.58 | 2806.17 | 63485.19 | 43559.1 | 2246.09 | 109619.49 | 44142.27 | 22755.36 | 3423.99 | 8297.25 | 631531.84 | 80.84 | 29303.08 |
| HG00380 | 44489.75 | 5278.06 | 55113.83 | 64084.27 | 2650.36 | 120172.16 | 34658.51 | 40185.75 | 2673.01 | 7226.19 | 586317.82 | 226.53 | 36923.77 |
| HG00381 | 28661.52 | 2112.57 | 63030.11 | 73656 | 3216.14 | 113290.24 | 48967.37 | 36291.98 | 4099.01 | 9017.81 | 598202.74 | 94.59 | 19359.92 |
| HG00382 | 49850.86 | 4191.61 | 32421.58 | 48954.05 | 1910.59 | 121283.61 | 28463.92 | 31914.69 | 2066.56 | 10215.82 | 610278.21 | 116.98 | 58292.55 |
| HG00383 | 34359.84 | 7934.47 | 69679.37 | 65994.89 | 4404.23 | 211094.55 | 60408.21 | 38095.73 | 4729.83 | 17856.84 | 463438.04 | 137.1 | 21866.91 |
| HG00384 | 33679.84 | 4586.07 | 79387.69 | 57527.41 | 2821.13 | 151618.33 | 54386.65 | 35013.97 | 3800.88 | 9582.11 | 527245.44 | 97.28 | 40232.36 |
| HG01334 | 45290.39 | 1766.65 | 76017.88 | 31200.63 | 1098.19 | 100998.35 | 14762.57 | 10278.67 | 1692.86 | 4397.08 | 678834.97 | 73.79 | 33587.99 |
| HG01789 | 26252.66 | 2322.06 | 33628.23 | 19510.39 | 991.51 | 57789.12 | 20047.72 | 15390.82 | 2584.33 | 4944.76 | 769284.9 | 217.49 | 47036.02 |
| HG01790 | 21964.42 | 942.63 | 41174.77 | 25235.23 | 1220.88 | 88436.88 | 17501.12 | 5894.28 | 2163.51 | 5877.24 | 772099.28 | 0 | 17489.76 |
| HG01791 | 37986.08 | 2504.58 | 28719.15 | 29703.09 | 1091.28 | 64480.93 | 18897.63 | 14478.84 | 1675.68 | 4418.79 | 748373.52 | 166.97 | 47503.47 |
| HG02215 | 21244.66 | 2328.45 | 74414.04 | 40257.09 | 2501.64 | 148577.92 | 43085.86 | 14509.49 | 2424.66 | 10468.38 | 617942.5 | 0 | 22245.31 |
| NA06984 | 33589.86 | 3959.1 | 68791.15 | 62123.19 | 2333.79 | 122648.85 | 43119.5 | 23462.9 | 2444.92 | 7140.28 | 603589.59 | 166.7 | 26630.18 |
| NA06985 | 23332.37 | 3268.41 | 78019.56 | 61669.7 | 1993.89 | 83047.28 | 45796.81 | 59495.98 | 1485.64 | 6130.22 | 611098.51 | 164.2 | 24497.42 |
| NA06986 | 28079.75 | 3493.9 | 74528.16 | 51284.67 | 1195.78 | 99751.76 | 22036.57 | 49424.57 | 1746.09 | 8574.45 | 611565.52 | 182.58 | 48136.22 |
| NA06994 | 54026.26 | 1661.13 | 41132.73 | 67750.36 | 3045.4 | 93221.01 | 23097.61 | 13724.09 | 2056.64 | 3678.22 | 666429.36 | 79.1 | 29030.22 |
| NA07000 | 22247.48 | 5256.43 | 71241.18 | 56570.54 | 1960.51 | 120197 | 25770.71 | 35421.7 | 2500.36 | 6686.56 | 607955.68 | 66.3 | 44125.59 |
| NA07037 | 15152.13 | 3005.17 | 58502.36 | 33531.66 | 1717.24 | 79993.13 | 36476.22 | 17738.09 | 3646.61 | 9106.43 | 715776.4 | 35.35 | 25319.2 |
| NA07048 | 14917.94 | 1701.23 | 29951.1 | 17066.5 | 806.56 | 43832.03 | 17310.5 | 7733.47 | 2738.23 | 3690.51 | 824834.54 | 23.72 | 35393.67 |
| NA07051 | 41597.03 | 3451.05 | 44885.29 | 50040.15 | 2040.24 | 123857.79 | 24124.76 | 36680.92 | 1985.98 | 10602.74 | 610466 | 86.82 | 46654.22 |
| NA07056 | 22850.22 | 5384.03 | 95394.23 | 75427.4 | 1952.03 | 152219.95 | 38070.94 | 60181.17 | 2870.63 | 8752.23 | 510704.26 | 127.58 | 26065.32 |
| NA07347 | 35854.14 | 2910.01 | 67576.79 | 65061.97 | 4957.79 | 175318.84 | 22633.38 | 15915.21 | 3377.04 | 6466.68 | 567486.98 | 0 | 32441.17 |
| NA07357 | 34485.37 | 3427.71 | 45298.74 | 56301.49 | 3011.08 | 122317.96 | 39977.27 | 35223.94 | 2499.76 | 10775.49 | 617195.34 | 189.38 | 29296.47 |
| NA10851 | 53417.89 | 15738.97 | 51532.02 | 96677.68 | 3292.27 | 198392.86 | 79193.49 | 46807.78 | 1924.22 | 23237.68 | 400218.63 | 236.53 | 29329.98 |
| NA11829 | 14155.46 | 1321.87 | 48867.56 | 24667.97 | 1228.19 | 53187.06 | 12136.23 | 23699.99 | 1290.64 | 5714.23 | 788781.8 | 41.63 | 24907.37 |
| NA11830 | 30115.37 | 3704.33 | 44225.77 | 55932.59 | 3676.05 | 98801.04 | 47619.05 | 25421.33 | 4015.38 | 7832.82 | 646731.14 | 113.11 | 31727.18 |
| NA11831 | 31216.88 | 4964.21 | 124291.16 | 71553.41 | 3225.81 | 173449.85 | 37845.12 | 29617.92 | 3932.32 | 11183.42 | 480459.24 | 92.96 | 28167.7 |
| NA11832 | 28068.9 | 2797.82 | 40545.65 | 46474 | 1829.92 | 127761.9 | 19100.77 | 21701.98 | 3085.16 | 8106.11 | 666122.23 | 90.74 | 34314.84 |
| NA11840 | 15840.22 | 2001.55 | 68843.62 | 43528.32 | 1366.65 | 82165.76 | 18616.56 | 31287.66 | 3314.39 | 10050.79 | 688247.89 | 16.14 | 34720.43 |
| NA11843 | 43030.59 | 5203.53 | 44174.63 | 78920.18 | 1900.58 | 112115.73 | 32531.28 | 38712.77 | 1549.99 | 11606.45 | 593940.29 | 184.52 | 36111.01 |
| NA11881 | 27155.08 | 2555.68 | 56500.75 | 37686.1 | 1216.99 | 85091.88 | 27009.05 | 19260.88 | 2369.07 | 5297.96 | 705732.02 | 97.36 | 30027.18 |
| NA11892 | 38586.04 | 6665.17 | 67472.18 | 73328.09 | 3068.45 | 150219.18 | 85658.09 | 59109.81 | 3068.45 | 13172.98 | 474710.58 | 325.95 | 24615.04 |
| NA11893 | 24902.6 | 5118.68 | 121818.53 | 54138.71 | 1960.8 | 134286.99 | 49067.23 | 28030.45 | 2853.24 | 8860.08 | 538538.11 | 94.39 | 30321.62 |
| NA11894 | 34744.86 | 3454.93 | 46269.98 | 58512.16 | 1773.1 | 99932.21 | 43649.45 | 40794.24 | 2503.19 | 10130.11 | 626632.94 | 104.3 | 31498.53 |

| | | | | | | | | | | | | | | |
|---|---|---|---|---|---|---|---|---|---|---|---|---|---|---|
| NA11918 | 34783.38 | 4288 | 66681.46 | 33815.7 | 1260.65 | 124351.92 | 24014.56 | 25923.3 | 2565.7 | 11052.91 | 632581.68 | 17.76 | 38663 |
| NA11920 | 27516.03 | 2495.93 | 60519.38 | 45945.74 | 1963.09 | 138080.28 | 24501.28 | 20336.72 | 3622.38 | 10600.71 | 633354.83 | 46.74 | 31012.21 |
| NA11930 | 18876.52 | 18182.96 | 24524.75 | 27828.37 | 1152.35 | 61148.46 | 16643.93 | 16525.78 | 2284.76 | 4652.38 | 755337.12 | 90.53 | 52747.47 |
| NA11931 | 39443.5 | 6151.1 | 68206.57 | 83103.58 | 1702.85 | 129439.57 | 36524.34 | 70673.96 | 2096.7 | 13402.68 | 520839.61 | 266.43 | 28149.11 |
| NA11992 | 14431.33 | 2729.52 | 61702.48 | 41195.04 | 1162.07 | 73327.72 | 12643.18 | 50230.39 | 2288.11 | 9165.96 | 688280.63 | 45.04 | 42798.52 |
| NA11994 | 18380.4 | 3867.32 | 38864.45 | 51741.36 | 3017.36 | 98212.96 | 54907.46 | 31682.28 | 3697.33 | 8775.84 | 660801.94 | 63.75 | 25987.55 |
| NA12004 | 19680.22 | 5322.04 | 17823.17 | 69073.29 | 3079.99 | 85967.93 | 40017.21 | 24639.91 | 1517.35 | 16373.77 | 236796.81 | 90.59 | 10598.79 |
| NA12005 | 37135.52 | 20063.49 | 79348.33 | 104021.85 | 2408.58 | 117586.99 | 111315.04 | 97658.38 | 1329.54 | 19750.37 | 378662.85 | 337.2 | 30381.86 |
| NA12006 | 27945.4 | 4126.05 | 101174.21 | 75059.74 | 2845.26 | 167328.55 | 25847.99 | 86793.2 | 3292.24 | 15489.88 | 459126.31 | 77.36 | 30893.81 |
| NA12043 | 36613.65 | 3546.78 | 68520.99 | 91602.32 | 3219.38 | 156726.6 | 29397.32 | 65942.76 | 3287.59 | 9780.92 | 501978.01 | 109.13 | 28838.02 |
| NA12044 | 43586.81 | 910.23 | 64678.04 | 51388.74 | 1638.41 | 110059.29 | 9336.32 | 8009.99 | 754.19 | 4343.08 | 671018.41 | 78.02 | 34198.48 |
| NA12045 | 18647.11 | 2201.28 | 96966.66 | 53791.04 | 2413.76 | 182382.99 | 37268.72 | 21732.29 | 4742.52 | 11601.32 | 542865.4 | 25.5 | 25344.43 |
| NA12058 | 28573.18 | 2585.84 | 84583.26 | 47707.96 | 1267.33 | 89742.64 | 31013.66 | 18905.47 | 1261.18 | 10136.56 | 642276.27 | 81.9 | 41825.85 |
| NA12154 | 44339.33 | 4160.73 | 60205.14 | 81477.01 | 2041.75 | 123644.26 | 19925.18 | 62748.88 | 2205.86 | 11700.25 | 542020.03 | 91.71 | 45439.85 |
| NA12155 | 33109.56 | 4726.67 | 53249.3 | 107685.98 | 3539.3 | 207288.67 | 48294.29 | 27286.84 | 3082.61 | 17171.3 | 478056.35 | 91.34 | 16417.77 |
| NA12156 | 59465.44 | 3737.14 | 37311.65 | 90408.99 | 3916.53 | 188082.99 | 27654.87 | 22751.73 | 2451.57 | 9477.4 | 513334.13 | 119.59 | 41287.97 |
| NA12234 | 36280.13 | 3534.11 | 48111.73 | 46267.84 | 1621.94 | 74557.81 | 17653.49 | 45311.75 | 1621.94 | 5958.48 | 681605.55 | 119.51 | 37355.73 |
| NA12249 | 16003.6 | 1247.47 | 30770.96 | 32467.97 | 1180.04 | 49696.56 | 13328.84 | 12969.21 | 1584.63 | 3967.18 | 801798.16 | 22.48 | 34962.91 |
| NA12272 | 32894.36 | 3928.89 | 42480.38 | 43667.12 | 1820.42 | 88359.66 | 31154.59 | 25393.75 | 3053.24 | 7224.08 | 683361.56 | 380.21 | 36281.73 |
| NA12273 | 49178.69 | 8032.17 | 187156.92 | 76389.51 | 1840.27 | 182181.37 | 30078.7 | 56377.24 | 2044.74 | 10837.14 | 356776.23 | 262.15 | 38844.88 |
| NA12275 | 55374.15 | 6530.61 | 74931.97 | 96700.68 | 5102.04 | 209013.61 | 38537.41 | 50918.37 | 3095.24 | 7789.12 | 422585.03 | 102.04 | 29319.73 |
| NA12282 | 51250.34 | 2087.12 | 79696.98 | 76914.16 | 3246.63 | 167046.73 | 17044.8 | 16329.76 | 3169.33 | 7382.21 | 540157.69 | 0 | 35674.26 |
| NA12283 | 45967.94 | 3613.91 | 51220.84 | 52971.81 | 1277.59 | 108432.63 | 24701.85 | 25592.61 | 2707.89 | 6937.69 | 623267.49 | 142.52 | 53165.23 |
| NA12286 | 29366.45 | 5972.84 | 55011.73 | 57073.78 | 3270.84 | 135858.36 | 80633.31 | 41478.04 | 5261.79 | 13581.1 | 546135.43 | 426.63 | 25929.7 |
| NA12287 | 48709.37 | 2446.68 | 39585.29 | 38810.5 | 1926.76 | 102556.78 | 20786.61 | 19267.63 | 3129.71 | 5046.28 | 680697.71 | 152.92 | 36863.35 |
| NA12340 | 18461.12 | 4232.25 | 66998.03 | 63463.57 | 3038.93 | 115540 | 92912.37 | 30424.69 | 3281.64 | 12388.32 | 562874.49 | 75.85 | 26288.51 |
| NA12341 | 35836.28 | 2816.56 | 38033.6 | 31092.07 | 1198.54 | 75048.44 | 26907.17 | 25998.28 | 1797.81 | 7690.62 | 728211.58 | 249.7 | 25119.35 |
| NA12342 | 17954.5 | 4070.65 | 127014.13 | 107872.36 | 4700.64 | 229677.01 | 101693.68 | 49647.45 | 4143.35 | 20644.04 | 315693.83 | 24.23 | 16864.14 |
| NA12347 | 29655.97 | 2131.63 | 41346.95 | 31652.08 | 901 | 52587.44 | 15001.96 | 22671.42 | 2311.09 | 3776.13 | 748889.32 | 69.59 | 49005.42 |
| NA12348 | 57334.56 | 4775.36 | 66839.95 | 118945.79 | 5546.07 | 207501.55 | 25115.98 | 50216.86 | 3128.16 | 9988.97 | 411210.01 | 151.12 | 39245.61 |
| NA12383 | 35888.07 | 4902.74 | 64875.53 | 77892.31 | 3199.04 | 155907.19 | 48512.63 | 49137.73 | 2046.89 | 13887.02 | 512201.7 | 98.05 | 31451.09 |
| NA12399 | 48773.67 | 4141.03 | 77827.49 | 51436.71 | 1571.2 | 120023.43 | 16763.87 | 46177.2 | 2210.33 | 7589.68 | 567534.82 | 173.1 | 55764.16 |
| NA12400 | 28699.66 | 5323.29 | 77060.43 | 71602.75 | 1704.03 | 141482.26 | 28738.06 | 56012.1 | 2784.04 | 9278.55 | 530888.49 | 148.8 | 46272.74 |
| NA12413 | 29503.01 | 3912.26 | 62714.62 | 78302.1 | 1908.77 | 130961.35 | 34713.05 | 43982.17 | 2609.75 | 11040.54 | 546087.5 | 61.57 | 54203.31 |
| NA12489 | 28289.35 | 4271.88 | 62381.34 | 62238.94 | 2622.46 | 113145.53 | 50942.19 | 43347.73 | 1993.54 | 14524.4 | 591904.78 | 59.33 | 24278.53 |
| NA12546 | 121294.67 | 2918.86 | 39826.17 | 98851.92 | 9859.25 | 146331.97 | 26788.61 | 26529.16 | 4280.99 | 7070.12 | 484724.65 | 129.73 | 31393.92 |
| NA12716 | 101582.44 | 0 | 1020.93 | 1327.21 | 102.09 | 2041.86 | 408.37 | 918.84 | 0 | 306.28 | 836242.98 | 306.28 | 55742.73 |
| NA12717 | 24240.85 | 2594.15 | 95709.57 | 40713.68 | 1787.08 | 141611.54 | 54650.01 | 19715.51 | 2262.67 | 12307.78 | 589404.36 | 28.82 | 14973.99 |
| NA12718 | 14018.36 | 1352.02 | 40959.23 | 36533.12 | 1650.89 | 66363.05 | 36803.53 | 14303 | 3031.38 | 6404.33 | 760592.04 | 71.16 | 17917.88 |
| NA12749 | 34279.7 | 5021.06 | 74730.91 | 54022.7 | 2310.66 | 156744.79 | 41426.18 | 29853.37 | 3080.88 | 15550.66 | 554900.16 | 185.24 | 27893.69 |
| NA12750 | 30005.21 | 2789.08 | 62900.43 | 37931.51 | 1275.01 | 111457.02 | 23762.98 | 23826.73 | 3548.77 | 8128.18 | 659386.72 | 58.44 | 32810.23 |
| NA12751 | 42936.32 | 3612.36 | 85488.05 | 61602.49 | 2259.44 | 154349.6 | 32518.15 | 34791.33 | 2280.05 | 7822.21 | 505895.85 | 137.35 | 45271.31 |
| NA12760 | 41614.38 | 2827.98 | 41296 | 44348.72 | 1929.02 | 99971.91 | 38917.5 | 18409.96 | 3127.63 | 7716.08 | 673284.02 | 56.19 | 26500.61 |
| NA12761 | 20352.09 | 2727.74 | 82904.64 | 38890.1 | 1807.46 | 107679.37 | 18882.29 | 23404.24 | 3330.22 | 9010.8 | 653087.57 | 52.97 | 37870.51 |
| NA12762 | 39135.17 | 23173.85 | 95207 | 95399.56 | 2695.8 | 154656.96 | 105705.55 | 48114.19 | 1808.36 | 17874.34 | 372677.8 | 531.63 | 43019.8 |
| NA12763 | 40294.56 | 3096.6 | 46458.74 | 44842.7 | 1616.04 | 99817.11 | 28924.22 | 22972.93 | 3164.34 | 5157.78 | 657602.65 | 125.8 | 45926.51 |
| NA12775 | 16757.95 | 3903.38 | 106842.15 | 79682.01 | 4312.11 | 171625.93 | 49088.53 | 30511.73 | 3412.9 | 14326 | 505763.1 | 61.31 | 13712.91 |
| NA12776 | 14589.28 | 2690.07 | 73608.05 | 46311.46 | 1648.75 | 84097.15 | 23641.14 | 24552.29 | 3617.49 | 7630.9 | 688348.1 | 65.08 | 29194.82 |
| NA12778 | 18070.23 | 2423.87 | 51243.01 | 40568.68 | 1926.66 | 83312.62 | 62476.69 | 20898.07 | 3760.1 | 6370.42 | 687259.17 | 15.54 | 21674.95 |
| NA12812 | 37807.84 | 4949.11 | 84913.24 | 62350.36 | 2715.67 | 197769.94 | 31073.66 | 24914.77 | 2461.87 | 13823.68 | 506247.73 | 135.36 | 30836.78 |
| NA12813 | 27610.17 | 4201.17 | 37992.08 | 52618.38 | 2800.78 | 124799.02 | 26270.29 | 33497 | 4304.9 | 8549.3 | 643341.23 | 164.24 | 33851.42 |
| NA12814 | 30516.83 | 2027.52 | 104072.13 | 49574.07 | 2294.89 | 146182.24 | 16598.96 | 13761.91 | 2837.05 | 7731.33 | 594755.17 | 22.28 | 29625.61 |
| NA12815 | 40447.17 | 4008.46 | 96102.33 | 50478.4 | 1752.44 | 145895.86 | 24211.9 | 32208.68 | 1792.73 | 10877.23 | 555705.51 | 120.86 | 36398.43 |
| NA12827 | 67650.65 | 4706.33 | 77643.02 | 72776.51 | 2189.17 | 126376.81 | 22608.7 | 41151.79 | 2456.14 | 7429.44 | 534149.5 | 205.95 | 40655.99 |
| NA12829 | 77304.45 | 3351.43 | 94227.79 | 88522.05 | 5124.09 | 182085.09 | 20551.74 | 38638.38 | 2769.78 | 7866.16 | 442942.61 | 0 | 36616.44 |
| NA12830 | 29865.89 | 3649.25 | 72787.39 | 68624.35 | 2397.7 | 123626.59 | 26967.57 | 68426.74 | 1291.07 | 12950.23 | 566134.43 | 105.39 | 23173.4 |
| NA12842 | 24022.85 | 2518.26 | 56998.77 | 39583.03 | 1391.67 | 88178.77 | 19907.49 | 15480.66 | 3180.96 | 7064.37 | 697550.66 | 33.13 | 44089.38 |
| NA12843 | 23830.98 | 2794.01 | 46105.19 | 36395.81 | 942.26 | 66224.49 | 16256.03 | 39517.56 | 2707.97 | 8128.02 | 720886.22 | 110.61 | 36096.75 |
| NA12872 | 29026.04 | 3228.74 | 76213.73 | 51015.71 | 1296.39 | 132231.54 | 23681.5 | 22348.42 | 3139.05 | 8679.27 | 617948.85 | 53 | 31137.76 |
| NA12873 | 67185.31 | 4628.22 | 69560.32 | 52691.68 | 1994.4 | 135284.09 | 14752.45 | 26231.65 | 2207.54 | 6333.35 | 556863.16 | 228.37 | 62039.46 |
| NA12874 | 102523.88 | 3964.07 | 51333.24 | 78254.67 | 3194.07 | 113018.68 | 26008.84 | 24525.88 | 2338.51 | 5447.03 | 554370.45 | 199.63 | 34821.05 |
| NA12889 | 27846.98 | 2340.97 | 67376.56 | 49738.66 | 1245.55 | 70690.61 | 17815.84 | 25528.25 | 2535.59 | 5243.55 | 696346.75 | 44.48 | 33246.22 |
| NA12890 | 41363.97 | 2309.54 | 30066.78 | 31117.68 | 1503.03 | 93010.89 | 27800.01 | 16331.74 | 1967.39 | 6775.87 | 712279.05 | 79.43 | 35394.61 |
| NA18486 | 33337 | 2288.55 | 94675.14 | 40508.62 | 2545.56 | 148413.31 | 24990.52 | 9019.59 | 3328.81 | 5703.03 | 602477.02 | 134.62 | 31843.94 |
| NA18488 | 51621.78 | 7076.86 | 106388.83 | 122469.04 | 4993.12 | 320503.24 | 37703.95 | 36327.89 | 2791.43 | 17181.05 | 274346.37 | 471.79 | 18124.63 |
| NA18489 | 13299.36 | 1337.12 | 34964.91 | 22195.35 | 1137.23 | 101651.89 | 18236.4 | 6918.46 | 3260.31 | 6817.55 | 767464.02 | 19.41 | 22666.94 |
| NA18498 | 24192.13 | 5157.61 | 57607.69 | 48807.84 | 2073.18 | 140603.7 | 29782.27 | 26814.19 | 3433.43 | 7863.19 | 599934.97 | 71.59 | 53658.2 |
| NA18499 | 32455.4 | 8296.11 | 91676.23 | 52039.25 | 2405.03 | 170765.84 | 63025.32 | 33896.74 | 3787.72 | 15067.08 | 494506.97 | 142.46 | 31567.13 |
| NA18502 | 20552.57 | 3083.98 | 109834.53 | 46123.75 | 1684.57 | 164622.38 | 14998.77 | 11598.93 | 1952.17 | 7440.16 | 585697.87 | 39.48 | 32370.85 |
| NA18505 | 23965.27 | 15216.63 | 45076.28 | 35614.07 | 846.64 | 81376.38 | 12029.67 | 17139.36 | 1791.95 | 3905.11 | 699723.98 | 110.13 | 63204.53 |
| NA18508 | 14059.81 | 1448.79 | 45118.17 | 25806.5 | 1094.82 | 92327.2 | 18200.38 | 7301.55 | 2049.7 | 6437.22 | 743359.04 | 0 | 28399.5 |
| NA18510 | 27894.97 | 3100.04 | 49300.75 | 32745.81 | 1713.32 | 86843.85 | 27777.18 | 14520.38 | 3164.29 | 6264.32 | 711214.74 | 85.67 | 35374.68 |
| NA18511 | 33475.29 | 3022.39 | 114255.62 | 71392.59 | 3686.4 | 209621.28 | 16806.34 | 15478.32 | 2221 | 8403.17 | 486078.67 | 160.28 | 35375.74 |
| NA18517 | 30815.97 | 5837.88 | 65122.77 | 67432.67 | 1789.98 | 210772.59 | 33512.08 | 29880.12 | 3438.85 | 11972.85 | 498113.46 | 349.08 | 30140.08 |
| NA18519 | 29046.71 | 4867.98 | 93071.99 | 54869.6 | 3030.4 | 175311.91 | 46777.78 | 40491.31 | 2353.4 | 16699.44 | 512331.15 | 193.43 | 20954.9 |
| NA18520 | 24044.94 | 2865.8 | 52000.75 | 26053.45 | 1820.72 | 117922.26 | 43754.44 | 15267.92 | 2261.61 | 8327.96 | 669722.97 | 16.33 | 35940.86 |
| NA18858 | 30476.05 | 3416.05 | 50500.61 | 60089.96 | 2139.1 | 158553.55 | 26238.52 | 22708.6 | 3212.71 | 9898.41 | 600346.49 | 154.54 | 32037.67 |
| NA18861 | 44855.7 | 6799.83 | 44636.76 | 71977.75 | 1674.2 | 121199.24 | 18532.11 | 26812.97 | 1687.08 | 9761.88 | 596530.54 | 231.81 | 55300.13 |
| NA18867 | 17726.53 | 2914.11 | 48146.14 | 28438.48 | 2015.69 | 121275.3 | 45761.87 | 18060.56 | 3616.72 | 8247.04 | 668379.04 | 0 | 30903.37 |
| NA18868 | 53704.08 | 3230.89 | 72707.85 | 48755.98 | 1488.25 | 99038.36 | 15060.55 | 20377.53 | 1246.57 | 6550.83 | 599712.53 | 216.24 | 77910.35 |
| NA18870 | 18205.07 | 3304.43 | 40732.5 | 27478.96 | 1007.7 | 76467.43 | 12649.94 | 17795.85 | 2803.14 | 7504.03 | 732248.91 | 153.46 | 59643.47 |
| NA18873 | 58070.85 | 4498.51 | 54673.5 | 61792.57 | 1306.02 | 155953.91 | 15697.82 | 20947.5 | 2944.94 | 8664.11 | 567153.22 | 213.4 | 48083.65 |
| NA18907 | 11372.46 | 1102.6 | 37700.45 | 17257.23 | 1007.69 | 61981.43 | 12239.36 | 8020.36 | 2121.36 | 5112.78 | 806949.72 | 7.91 | 35126.66 |
| NA18908 | 16255.39 | 2656.72 | 51933.79 | 31659.26 | 1387.97 | 109811.13 | 17345.32 | 14714.15 | 2401.27 | 6982.41 | 709387.08 | 42.58 | 34537.37 |
| NA18909 | 37718.35 | 5251.32 | 62355.55 | 80971.02 | 3223.12 | 196893.23 | 22058.71 | 20392.12 | 2704.27 | 8977.56 | 526248.76 | 78.61 | 33127.37 |
| NA18910 | 22254.34 | 1619.52 | 27979.17 | 33487.44 | 1803.13 | 97628.15 | 16731.95 | 7400.85 | 1854.92 | 4594.93 | 744656.51 | 56.5 | 39932.58 |
| NA18912 | 47747.72 | 2272.24 | 123867.72 | 73919.41 | 4042.95 | 156917.53 | 31739.7 | 23213.68 | 4094.12 | 9017.31 | 474396.37 | 122.82 | 45557.36 |
| NA18916 | 47930.83 | 6089.6 | 86277.02 | 84613.45 | 2663.25 | 183756.48 | 31012.72 | 27456.64 | 2899.81 | 8462.87 | 484497.46 | 190.78 | 34141.47 |
| NA18917 | 24932.48 | 2522.55 | 66388.93 | 34589.51 | 955.51 | 88837.08 | 29302.35 | 14702.14 | 2649.95 | 5032.36 | 696886.31 | 63.7 | 33137.13 |
| NA18923 | 19563.45 | 3403.65 | 39192.16 | 37490.34 | 1752.03 | 93786.08 | 33569.61 | 20261.25 | 3750.04 | 8765.15 | 493017 | 105.42 | 19292.36 |
| NA18933 | 17632.85 | 2135.77 | 59191.46 | 41927.28 | 1792.52 | 152453.6 | 26697.18 | 9242.31 | 3089.24 | 11861.17 | 644215.61 | 63.56 | 29328.76 |
| NA18934 | 20666.34 | 27630.93 | 37127.64 | 34517.18 | 1341.87 | 84403.97 | 21270.3 | 14770.69 | 2527.06 | 4412.26 | 703499.48 | 88.45 | 47743.84 |
| NA19092 | 34138.63 | 4434.54 | 54900.69 | 39586.54 | 1629.51 | 142456.42 | 39578.44 | 20186.46 | 2432.1 | 9233.89 | 580308.07 | 202.68 | 36141.06 |
| NA19093 | 32843.6 | 4367.33 | 66901.24 | 39429.51 | 1633.05 | 123010.39 | 17528.4 | 20450.7 | 1890.9 | 6639.63 | 640305.12 | 85.95 | 44908.81 |
| NA19095 | 24394.67 | 3538.85 | 52408.55 | 50909.87 | 1449.78 | 110368.17 | 20971.11 | 16572.87 | 2389.51 | 6581.64 | 669678.01 | 27.95 | 40132.61 |
| NA19096 | 46719.45 | 3699.32 | 17228.84 | 37346.47 | 3055.05 | 103310.68 | 44578.84 | 15233.7 | 2078.27 | 8666.38 | 672465.03 | 290.96 | 43082.49 |
| NA19098 | 42953.19 | 3853.19 | 50564.87 | 24685.15 | 1325.67 | 85367.36 | 13387.82 | 16068.29 | 1347.52 | 6628.35 | 668851.84 | 167.53 | 73305.22 |
| NA19099 | 17187.24 | 1605.97 | 37286.49 | 19207.04 | 960.54 | 72127.96 | 22988.46 | 6951.59 | 1788.2 | 6347.93 | 778551.44 | 60.75 | 22119.04 |
| NA19102 | 32408.44 | 4661.62 | 45472.61 | 37554.64 | 1666.94 | 99212.08 | 24713.37 | 15554.89 | 1725.09 | 8305.63 | 684240.62 | 155.06 | 44329.01 |
| NA19107 | 30211.08 | 2211.97 | 27341.28 | 21363.2 | 752.4 | 64969.45 | 11894.48 | 10883.06 | 1924.17 | 4415.72 | 770575.85 | 102.79 | 53350.44 |
| NA19108 | 12449.42 | 3678.4 | 68657.21 | 32998.66 | 662.41 | 78824.84 | 26843.56 | 17745.74 | 2101.53 | 10695.22 | 382255.14 | 61.55 | 28253.37 |
| NA19113 | 34015.76 | 4523.14 | 79633.58 | 53214.94 | 1422.81 | 127789.63 | 17767.59 | 25663.32 | 1835.6 | 7465.37 | 587437.09 | 131.74 | 59081.85 |
| NA19114 | 8845.13 | 1381.23 | 23523.98 | 14201.28 | 434.48 | 36411.06 | 7042.59 | 7230.5 | 924.01 | 3170.6 | 195160.19 | 22.74 | 13101.33 |
| NA19116 | 56872.76 | 3730.2 | 51814 | 65482.88 | 3755.75 | 128206.44 | 21435.87 | 14103.22 | 2299.44 | 7153.81 | 606591.72 | 127.75 | 37659.68 |
| NA19117 | 26357.63 | 3823.61 | 63786.6 | 32129.73 | 1092.46 | 94726.03 | 21743.21 | 20593.68 | 2152.31 | 5144.34 | 686178.76 | 32.61 | 42067.85 |
| NA19118 | 53495.08 | 5228.33 | 98671.13 | 75347.87 | 3444.71 | 204354.22 | 16951.23 | 19265.86 | 2695.86 | 5323.64 | 477126.05 | 217.85 | 37810.09 |
| NA19119 | 21982.08 | 9438.3 | 36320.65 | 24498.08 | 768.96 | 75857.1 | 12629.4 | 16018.1 | 1910.05 | 8305.44 | 607296.4 | 77.39 | 42421.29 |
| NA19121 | 30086.68 | 3537.83 | 57022.64 | 57362.62 | 2538.08 | 140462.85 | 23593.37 | 19187.07 | 3352.69 | 7570.48 | 616083.48 | 30.3 | 39171.93 |
| NA19129 | 15553.77 | 3350.22 | 47099.76 | 24637.58 | 860.04 | 73474.27 | 19741.54 | 23271.63 | 2833.07 | 7065.81 | 739813.04 | 101.18 | 41883.32 |
| NA19130 | 18920.95 | 4668.87 | 106746.11 | 41597.65 | 1158.61 | 113773.5 | 34611.55 | 22201.78 | 2521.42 | 11173.16 | 605585.2 | 117.01 | 36155.61 |
| NA19131 | 27701.83 | 3972.29 | 86838.72 | 45993.99 | 1599.95 | 124753.26 | 34150.68 | 23294.31 | 3077.3 | 8183.66 | 597376.33 | 91.95 | 42892.17 |
| NA19137 | 28952.49 | 2363.03 | 32922.82 | 33369.88 | 1468.91 | 79949.33 | 26238.2 | 15359.72 | 1862.75 | 8600.59 | 742759.22 | 106.44 | 26046.6 |
| NA19138 | 16866.47 | 54088.25 | 84977.44 | 68138.76 | 2105.02 | 178503.67 | 19316.71 | 28294.12 | 2205.95 | 7714.98 | 481065.82 | 117.03 | 41926.26 |
| NA19141 | 21488.15 | 3903.41 | 71329.39 | 40665.33 | 1602.14 | 124753.61 | 11486.89 | 27304.42 | 1602.14 | 9292.43 | 633109.03 | 155.36 | 53307.7 |
| NA19143 | 29377.51 | 3893 | 43846.87 | 32848.58 | 1468.31 | 95946.67 | 18007.93 | 16331.47 | 2745.35 | 4888.75 | 687316.81 | 219.4 | 63109.34 |
| NA19144 | 16352.26 | 11822.57 | 62005.7 | 53861.99 | 3573.51 | 164576.04 | 146222.29 | 13848.37 | 2382.34 | 23029.3 | 482942.76 | 145.86 | 18426.68 |
| NA19146 | 22283.09 | 3084.48 | 93223.76 | 41725.91 | 1554.27 | 114308.67 | 27168.46 | 17622.69 | 2371.1 | 10331.33 | 635643.81 | 75.79 | 30332.34 |
| NA19147 | 25741.1 | 2826.23 | 47319.99 | 35966 | 1272.85 | 98612.84 | 23851.1 | 16077.23 | 3043.63 | 6739.46 | 703698.64 | 119.22 | 34475.75 |
| NA19149 | 22935.09 | 2556.15 | 41697.78 | 28583.22 | 1431.8 | 72512.14 | 35215.16 | 13237.53 | 2415.61 | 7633.32 | 744481.43 | 61.49 | 26782.5 |
| NA19152 | 36557.63 | 5472.11 | 39733.21 | 45018.52 | 2131.7 | 116958.04 | 41414.4 | 21811.51 | 2384.43 | 10065.16 | 638676.15 | 76.92 | 39656.29 |
| NA19153 | 20327.8 | 2237.34 | 41141.43 | 27193.23 | 1559.74 | 115063.03 | 18346.16 | 13436.8 | 1789.87 | 8553.02 | 719911.02 | 51.14 | 30018.67 |
| NA19159 | 20521.4 | 9198.6 | 52544.57 | 40312.74 | 1474.22 | 88001.32 | 24336.48 | 18383.06 | 2957.87 | 6772.96 | 698179.59 | 127.17 | 37180.6 |
| NA19160 | 27036.02 | 1177.15 | 51973.64 | 48250.27 | 3723.37 | 132429.15 | 19013.5 | 7625.87 | 3493.06 | 6896.55 | 678881.71 | 0 | 19499.71 |
| NA19171 | 24922.99 | 4052.3 | 29269.63 | 30227.94 | 965.16 | 83831.88 | 17194.88 | 17010.06 | 2347.87 | 4764.19 | 726921.76 | 75.3 | 58416.04 |
| NA19172 | 19767.95 | 2791.12 | 86049.6 | 36314.24 | 1610.83 | 117739.19 | 16709.6 | 19998.07 | 3770.98 | 13725.48 | 637585.09 | 51.96 | 43885.89 |
| NA19175 | 19541.55 | 2693.41 | 75300.86 | 85616.05 | 3209.17 | 290659.03 | 37363.9 | 18681.95 | 3266.48 | 15300.86 | 432779.37 | 0 | 15587.39 |
| NA19184 | 17638.36 | 1176.98 | 45853.19 | 23188.15 | 1315.93 | 112883.85 | 27757.12 | 7478.73 | 2427.52 | 7764.8 | 726883.37 | 40.87 | 25591.15 |
| NA19185 | 13220.13 | 2938.08 | 108484.11 | 53594.64 | 1583.94 | 157418.34 | 17559.29 | 22513.75 | 3195.07 | 12646.84 | 575317.65 | 88.96 | 31431.78 |
| NA19189 | 37194.08 | 3026.08 | 95693.04 | 36972.11 | 1023.79 | 108669.41 | 34498.69 | 20514.39 | 2074.77 | 6507.43 | 597460.9 | 140.43 | 56215.81 |
| NA19190 | 32829.77 | 21866.82 | 72740.32 | 72259.26 | 2692.21 | 159591.53 | 105949.87 | 26052.83 | 1738.54 | 13807.07 | 443379.19 | 295.38 | 46307.71 |
| NA19197 | 34616.27 | 6084.35 | 59526.55 | 62088.04 | 1567.18 | 151740.03 | 47298.59 | 24185.95 | 3733.58 | 8830.21 | 556915.68 | 111.94 | 43301.62 |
| NA19198 | 36159.38 | 5677.63 | 61292.23 | 47051.88 | 1582.97 | 142968.39 | 26817.94 | 29537.08 | 2808.5 | 11891.43 | 576871.4 | 159.57 | 55375.24 |
| NA19200 | 32217.33 | 3210.98 | 45873.94 | 38786.68 | 1968.02 | 123734.13 | 29313.11 | 18955.12 | 2529.74 | 6230.73 | 632128.09 | 79.68 | 54574.65 |
| NA19201 | 41938.02 | 6067.44 | 125799.23 | 69812.64 | 2284.56 | 192007 | 29239.42 | 27933.96 | 1364.8 | 10844.25 | 452832.71 | 59.34 | 39386.43 |
| NA19204 | 24341.71 | 2560.06 | 18338.66 | 22068.92 | 633.68 | 51408.25 | 11363.97 | 10823.23 | 2382.63 | 3565.5 | 795334.43 | 97.16 | 56760.72 |
| NA19206 | 36042.37 | 4489.79 | 84851.1 | 64434.38 | 2002.68 | 181062.54 | 26820.58 | 25893.08 | 3125.13 | 10373.77 | 526055.54 | 460.79 | 34388.24 |
| NA19207 | 30652.76 | 3587.29 | 32928.84 | 47574.87 | 2325.55 | 124639.72 | 34870.92 | 16996.33 | 2956.42 | 9413.54 | 659813.71 | 49.48 | 34178.2 |
| NA19209 | 16248.37 | 4512.13 | 45392.6 | 27025.69 | 987.42 | 66340.17 | 14136.02 | 22926.12 | 1644.04 | 7774.19 | 737352.54 | 211.85 | 55443.92 |
| NA19210 | 31805.79 | 19045.69 | 89221.07 | 51332.34 | 2208.54 | 136095.1 | 123681.91 | 22878.87 | 1573.12 | 22590.35 | 474208.46 | 264.48 | 22246.88 |
| NA19213 | 30045.86 | 5134.78 | 67267.08 | 55014.16 | 1625.78 | 161976.87 | 38498.95 | 25216.21 | 3413.65 | 9301.73 | 548787.58 | 123.96 | 53593.39 |
| NA19214 | 13633.78 | 3037.81 | 60087.64 | 28052.24 | 1154.13 | 90921.99 | 17893.75 | 17944.19 | 2697.67 | 7744.66 | 716920.72 | 35.19 | 39876.24 |
| NA19222 | 26121.88 | 3589.27 | 63980.1 | 52015.88 | 1661.7 | 140104.83 | 20111.29 | 18554.04 | 3228.44 | 8906.7 | 628558.41 | 170.92 | 32996.56 |
| NA19223 | 51061.16 | 2998.21 | 52619.18 | 37353.2 | 1976.98 | 88951.15 | 12660.55 | 10212.23 | 1571.11 | 3875.41 | 695164.9 | 117.83 | 41438.09 |
| NA19225 | 22161.57 | 2972.2 | 62975.71 | 27625.68 | 966.25 | 76627.51 | 15250.91 | 13431.43 | 2593.62 | 6323 | 715493.32 | 50.86 | 53510.99 |
| NA19235 | 38793.62 | 5022.17 | 41919.28 | 30140.56 | 999.16 | 88185.84 | 13950.47 | 14233.25 | 1519.47 | 3295.33 | 710648.36 | 154.59 | 51134.14 |
| NA19236 | 61573.12 | 4208.86 | 40479.31 | 57537.57 | 1435.96 | 116511.1 | 16191.73 | 20153 | 1634.03 | 4976.36 | 624495.56 | 99.03 | 50704.36 |
| NA19247 | 55117.83 | 4185.72 | 60604.99 | 74322.9 | 2884.28 | 197502.64 | 23355.61 | 26275.06 | 4502.29 | 9250.79 | 503587.76 | 175.87 | 38234.26 |
| NA19248 | 28916.03 | 4450.27 | 59422.97 | 41836.87 | 1913.4 | 157070.99 | 30743.43 | 24207.77 | 3181.84 | 11136.43 | 584125.21 | 43 | 50866.41 |
| NA19256 | 17008.96 | 3370.05 | 31198.66 | 30927.93 | 914.86 | 51671.02 | 15029.87 | 19856.24 | 3183.35 | 3416.73 | 757682.97 | 252.05 | 62005.23 |
| NA19257 | 11781.69 | 1815.05 | 61893.24 | 25410.76 | 1042.54 | 97893.04 | 13944.95 | 11233.32 | 3809.42 | 8938.7 | 728644.7 | 17.72 | 32393.66 |
| NA20502 | 47612.29 | 6425.33 | 56449.65 | 67881.47 | 3648.45 | 198556.83 | 57706.34 | 31761.8 | 7742.82 | 12789.85 | 482163.13 | 101.35 | 27160.69 |
| NA20504 | 39609.01 | 2900.79 | 36198.38 | 44408.5 | 1564.67 | 84667.99 | 27056.49 | 23100.86 | 1634.99 | 9089.15 | 699389.95 | 158.23 | 30220.99 |
| NA20505 | 14947.1 | 825 | 26545.67 | 18611.08 | 994.86 | 77404.64 | 25623.6 | 8371.35 | 2208.09 | 3300.01 | 791347.18 | 0 | 29821.41 |
| NA20506 | 22598.61 | 4518.19 | 69138.25 | 43317.84 | 2377.99 | 81450.12 | 60723.22 | 33790.52 | 5492.4 | 7272.06 | 639925.75 | 107.39 | 29287.68 |
| NA20507 | 26305.73 | 3670.57 | 41003.29 | 45667.97 | 2798.81 | 138961.54 | 55119.68 | 31582.17 | 3349.39 | 12846.98 | 611057.58 | 30.59 | 27605.72 |
| NA20508 | 38605.64 | 3111.26 | 29223 | 35282.62 | 2361.95 | 137416.52 | 23456.59 | 23065.65 | 3290.44 | 10278.55 | 651409.02 | 32.58 | 42466.2 |
| NA20509 | 44032.97 | 5871.06 | 126152.57 | 90022.96 | 4930.19 | 225772.46 | 43807.16 | 41059.8 | 3123.71 | 10838.88 | 381468.52 | 225.81 | 22693.91 |
| NA20510 | 31728.67 | 6741.84 | 48173.17 | 64284.24 | 3887.73 | 160837.3 | 46412.69 | 43831.98 | 4414.54 | 10149.44 | 536153.22 | 66.68 | 41251.28 |
| NA20512 | 11356.57 | 1399.62 | 24534.28 | 29007.33 | 1383.25 | 45962.38 | 31949.81 | 13280.02 | 3539.98 | 4473.06 | 788321.77 | 20.46 | 21141.63 |
| NA20513 | 48354.74 | 20888.06 | 38355.46 | 51829.12 | 2883.38 | 125638.76 | 119155.64 | 28200.96 | 1235.73 | 11742.44 | 482843.02 | 752.18 | 68120.49 |
| NA20514 | 32252.48 | 7440.29 | 88201.34 | 35595.08 | 1577.51 | 103755.25 | 42405.33 | 19723.74 | 2722.17 | 7459.53 | 608574.37 | 216.43 | 50076.47 |
| NA20515 | 30806.12 | 2536.43 | 74248.29 | 70835.64 | 3873.82 | 215919.57 | 50036.89 | 30483.31 | 5349.57 | 16878.8 | 473621.1 | 0 | 23750.23 |
| NA20516 | 35232.3 | 3107.46 | 64257.5 | 50491.39 | 2456.97 | 127272.8 | 38363.27 | 23923.16 | 3522.84 | 8918.77 | 611653.19 | 39.19 | 30761.15 |
| NA20517 | 18594.61 | 5765.04 | 63208.89 | 30725.61 | 777.44 | 57342.45 | 18410.58 | 18342.97 | 2963.27 | 2967.02 | 729704.05 | 202.81 | 50991.51 |
| NA20518 | 34472.43 | 12778.37 | 77750.19 | 64096.8 | 2719.64 | 161822.55 | 138126.21 | 29805.68 | 2309.72 | 17878.68 | 434291.12 | 134.01 | 23814.59 |
| NA20519 | 43430.12 | 4306.1 | 32504.92 | 35802.17 | 1943.9 | 118061.02 | 53715.55 | 21136.81 | 2140.75 | 8193.9 | 641633.86 | 221.46 | 36909.45 |
| NA20520 | 21870.06 | 3095.22 | 52654.58 | 52780.31 | 1879.88 | 141805.16 | 35651.85 | 24959.29 | 4454.24 | 8423.54 | 614187.7 | 53.88 | 38172.33 |
| NA20521 | 50896.91 | 5100.98 | 45457.11 | 63169.39 | 2296.38 | 150996.67 | 29852.99 | 32281.14 | 4103.37 | 9166.71 | 570162.06 | 94.11 | 36045.7 |
| NA20525 | 24505.12 | 3708.76 | 34334.47 | 42161.55 | 1478.95 | 106985.21 | 59021.62 | 24414.11 | 4505.12 | 9579.07 | 656200.23 | 523.32 | 32582.48 |
| NA20527 | 25073.57 | 3169.85 | 63766.48 | 36994.54 | 2515.14 | 146597.44 | 60091.01 | 17638.36 | 5056.2 | 8815.94 | 597779.16 | 45.38 | 32456.93 |
| NA20528 | 48112.08 | 6318.11 | 89498.2 | 59853.89 | 2339.78 | 172520.88 | 36227.17 | 44105.13 | 3992.64 | 12543.2 | 478387.49 | 279.06 | 45815.24 |
| NA20529 | 13848.73 | 3528.34 | 156658.27 | 129529.26 | 2656.06 | 238917.58 | 39301.78 | 65421.29 | 3939.98 | 24041.71 | 309131.54 | 78.41 | 12947.05 |
| NA20530 | 25014.16 | 7020.22 | 111116.58 | 98073.75 | 2894.3 | 221481.88 | 58292.48 | 61026.68 | 4926.47 | 16146.51 | 367440.94 | 98.53 | 26467.47 |
| NA20531 | 44398.36 | 14383.55 | 54676.31 | 60426.94 | 2296.77 | 132909.3 | 122934.56 | 22155.62 | 2659.23 | 12724.58 | 486182.8 | 226.54 | 44025.44 |
| NA20532 | 32334.64 | 5634.82 | 57697.59 | 57635.12 | 2836.15 | 117256.8 | 44978.64 | 26325 | 4297.96 | 8408.51 | 600314.85 | 237.39 | 41917.59 |
| NA20534 | 28375.34 | 3846.72 | 66123.02 | 43642.3 | 2463.01 | 147180.92 | 59370.5 | 20958.64 | 4390.98 | 10184.13 | 521281.5 | 83.02 | 28864.25 |
| NA20535 | 40140.08 | 7830.53 | 53906.65 | 53791.84 | 1814.11 | 131500.09 | 35489.98 | 43240.14 | 2870.43 | 6579.02 | 569665.31 | 172.23 | 52850.34 |
| NA20536 | 21004.31 | 2415.42 | 44833.67 | 38717.42 | 1892.79 | 98297.9 | 45017.3 | 21541.07 | 4774.35 | 6822.52 | 664877.46 | 42.38 | 36499.75 |
| NA20538 | 55036.56 | 4874.09 | 67250.78 | 83990.95 | 4932.11 | 227080.19 | 39630.96 | 32958.11 | 7456.19 | 9371.01 | 420825.11 | 29.01 | 46564.93 |
| NA20539 | 42482.43 | 6770.98 | 40774.79 | 58883.68 | 1935.98 | 134804.02 | 43326.32 | 40546.44 | 3732.97 | 11735.04 | 576357.17 | 436.84 | 38173.62 |
| NA20540 | 38192.37 | 8509.28 | 50131.22 | 66463.88 | 3350.03 | 149121.24 | 57636.49 | 48699.75 | 3538.91 | 12922.98 | 524553.66 | 178.93 | 36701.26 |
| NA20541 | 29668.99 | 5176.88 | 83653.62 | 50258.84 | 1706.02 | 152953.17 | 30433.76 | 30257.28 | 3804.22 | 7726.1 | 562338.22 | 215.7 | 41807.2 |
| NA20542 | 34722.45 | 3089.36 | 109918.82 | 87077.27 | 2612.81 | 198968.02 | 17961.02 | 34607.42 | 3253.69 | 11913.76 | 475268.68 | 0 | 20606.7 |
| NA20543 | 47837.35 | 3729.6 | 57005.96 | 69360.27 | 2615.9 | 137192.44 | 55426.06 | 28205.13 | 4066.3 | 9712.51 | 552084.95 | 103.6 | 32659.93 |
| NA20581 | 45956.08 | 3051.12 | 49716.77 | 38399.22 | 1904 | 119443.23 | 33668.8 | 15977.01 | 2637.21 | 8041.72 | 644544.05 | 118.26 | 35643.75 |
| NA20582 | 19998.9 | 12473.78 | 63382.22 | 68299.47 | 3464.07 | 124627.87 | 129207.35 | 28906.5 | 2442.91 | 38505.35 | 492612.35 | 70.7 | 16008.55 |
| NA20585 | 33057.85 | 2831.57 | 84088.42 | 71878.42 | 5999.2 | 238356.32 | 38366.28 | 16055.96 | 6117.44 | 9540.23 | 471267.3 | 37.34 | 22210.74 |
| NA20586 | 25711.02 | 5432.56 | 60955.78 | 72489.95 | 3438.84 | 187430.17 | 52472.42 | 42089 | 4469.15 | 11975.73 | 501649.17 | 66.9 | 31819.31 |
| NA20588 | 29104.85 | 6158.32 | 105363.22 | 48652.52 | 890.65 | 133079.17 | 33319.55 | 26763.19 | 3571.06 | 11718.16 | 555666.91 | 214.55 | 45496.44 |
| NA20589 | 26948.77 | 2997.13 | 76130.49 | 43009.66 | 2074.28 | 170074.42 | 37650.39 | 16560.41 | 4275.57 | 13800.34 | 582154.38 | 33.87 | 24281.83 |
| NA20752 | 15746.08 | 1949.43 | 74356.99 | 36624.35 | 1068.49 | 84112.68 | 51628.74 | 20787.33 | 4015.38 | 11761.96 | 677129.96 | 73.89 | 20642.4 |
| NA20754 | 38918.36 | 14197.04 | 81122.88 | 80585.92 | 3329.11 | 167207.2 | 165198.99 | 33924.7 | 2308.9 | 15152.82 | 363109.17 | 257.74 | 34558.3 |
| NA20756 | 18420.68 | 1503.44 | 43971.94 | 33755.73 | 987.97 | 82538.66 | 15871.99 | 8655.5 | 3200.17 | 5841.92 | 765664.38 | 14.32 | 19573.31 |
| NA20757 | 38880.29 | 3488.27 | 56062.19 | 59906.02 | 3084.67 | 165265.27 | 59089.21 | 38697.71 | 3824.61 | 12271.41 | 527920.59 | 134.53 | 31375.22 |
| NA20758 | 18195.76 | 8195.61 | 81535.06 | 89625.41 | 3684.27 | 202800.04 | 86678 | 75414.67 | 5548.96 | 16120.54 | 388411.86 | 150.38 | 23639.45 |
| NA20759 | 44391 | 30105.01 | 74735.55 | 87900.51 | 4005.06 | 163287.6 | 150381.34 | 49775.79 | 2366.63 | 18885.1 | 337967.58 | 594.05 | 35604.78 |
| NA20760 | 22028.92 | 6749.35 | 40601.49 | 45627.94 | 1917.85 | 91735.34 | 31186.12 | 18050.96 | 3925.27 | 5795.7 | 701769.27 | 68.49 | 30543.32 |
| NA20761 | 23272.44 | 5687.91 | 72421.29 | 69434.37 | 2027.02 | 127998.41 | 36731.44 | 46938.03 | 4942.46 | 7863 | 564726.79 | 127.65 | 37829.2 |
| NA20765 | 43052.7 | 4889.47 | 66996.56 | 72924.7 | 3306.51 | 177662.67 | 38367.34 | 27037.2 | 4009.54 | 7946.52 | 508395.55 | 158.75 | 45252.5 |
| NA20766 | 34974.45 | 5621.05 | 48299.77 | 32783.5 | 1921.57 | 106566.58 | 42400.35 | 29227.69 | 2963.18 | 9877.25 | 639058.25 | 296.32 | 46010.04 |
| NA20768 | 45584.98 | 4727.94 | 67093 | 42742.75 | 1667.08 | 129376.08 | 44765.11 | 36211.09 | 4072.04 | 12380.09 | 579678.06 | 109.32 | 31592.47 |
| NA20769 | 19844.57 | 3697.3 | 52554.85 | 36435.29 | 1319.28 | 102654.07 | 25188.19 | 27222.54 | 5132.98 | 7538.72 | 658839.7 | 55.43 | 59511.54 |
| NA20770 | 17964.61 | 12176.29 | 59967.08 | 44937.03 | 1577.86 | 100004.68 | 32650.16 | 21196.87 | 3942.52 | 7200.3 | 674191.61 | 89.31 | 24093.16 |

| | | | | | | | | | | | | |
|---|---|---|---|---|---|---|---|---|---|---|---|---|
| NA20771 | 43079.2 | 4684.36 | 55102.04 | 67865.07 | 3352.01 | 181103.94 | 42169.82 | 30104.68 | 3732.68 | 8670.83 | 524109.13 | 31.72 | 35994.5 |
| NA20772 | 74472.47 | 6124.55 | 108594.96 | 145033.45 | 4837.88 | 294750.39 | 30211.01 | 52702.01 | 3757.08 | 8594.96 | 248018.53 | 205.87 | 22696.86 |
| NA20773 | 17212.6 | 2077.59 | 43351.13 | 28701.48 | 1260.76 | 80250.49 | 15442.8 | 18804.83 | 5238.36 | 7019.99 | 733246.13 | 35.51 | 46434.96 |
| NA20774 | 47262.9 | 3125.11 | 83675.69 | 83781.03 | 6531.13 | 202675.66 | 31496.89 | 24755.08 | 4845.68 | 7724.99 | 479124.97 | 70.23 | 24930.65 |
| NA20778 | 38446.75 | 18957.91 | 29045.61 | 63995.53 | 3451.05 | 162043.9 | 112667.29 | 24532.69 | 2041.34 | 17246.11 | 480465.39 | 173.93 | 46923.34 |
| NA20783 | 16153.24 | 1428.02 | 23578.95 | 34876.92 | 1268.61 | 68359.04 | 23525.82 | 11523.8 | 1633.92 | 6382.92 | 346311.72 | 26.57 | 12825.62 |
| NA20785 | 48790.16 | 3133.68 | 15271.72 | 75009.92 | 5394.68 | 116184.05 | 30702.1 | 19674.73 | 2697.34 | 5989.69 | 641253.47 | 119 | 35779.45 |
| NA20786 | 25623.03 | 2217.08 | 45701.13 | 43030.87 | 1821.65 | 123494.36 | 19531.61 | 12022.87 | 1675.03 | 7322.13 | 682380.23 | 71.09 | 35108.92 |
| NA20787 | 22223.28 | 2437.64 | 46049.51 | 33045.62 | 1062.32 | 83534.1 | 22356.07 | 20696.2 | 4125.96 | 6848.15 | 716816.85 | 94.85 | 40709.48 |
| NA20790 | 33433.56 | 3582.17 | 68333.4 | 89226.28 | 3656.41 | 204511.43 | 33142.78 | 26937.4 | 4615.37 | 8018.12 | 494951.56 | 43.31 | 29548.24 |
| NA20792 | 13570.97 | 2011.52 | 50901.37 | 37262.26 | 1512.73 | 58034.38 | 22693.7 | 20894.77 | 3570.59 | 5295.92 | 752732.46 | 10.9 | 31508.42 |
| NA20795 | 45004.75 | 4423.01 | 74041.68 | 45079.71 | 2224 | 127842.47 | 43330.5 | 28112.35 | 3873.26 | 9745.61 | 575965.82 | 174.92 | 39832.08 |
| NA20796 | 23291.57 | 2261.32 | 25950.88 | 27009.18 | 1085.43 | 63597.3 | 25779.02 | 15187.01 | 4414.09 | 4079.42 | 769942.56 | 63.32 | 37338.88 |
| NA20797 | 26968.42 | 2740.51 | 74279.86 | 59568.44 | 2665.22 | 179548.57 | 64161.06 | 20674.29 | 5458.43 | 13311.05 | 535506.17 | 30.12 | 15087.86 |
| NA20798 | 9078.02 | 1268.87 | 27112.76 | 18930.42 | 932.99 | 37954.13 | 24547.03 | 6913.47 | 3731.97 | 4469.03 | 839590.6 | 9.33 | 25461.36 |
| NA20799 | 35268.3 | 4013 | 35098.57 | 39414.66 | 1782.21 | 99597.49 | 37002.01 | 31073.45 | 4231.23 | 5989.19 | 669285.42 | 133.36 | 35219.81 |
| NA20800 | 31433.32 | 3515.02 | 63044.77 | 43735.9 | 1543.76 | 125080.16 | 28048.93 | 20496.38 | 3313.15 | 6661.92 | 640137.75 | 47.5 | 32573.33 |
| NA20801 | 37291.55 | 5140.62 | 78487.14 | 57677.36 | 3197.43 | 142700.68 | 33935.13 | 44446.01 | 3480.07 | 7189.8 | 556758.76 | 105.99 | 29589.46 |
| NA20802 | 26440.39 | 3988.94 | 61903.15 | 65484.24 | 3402.04 | 167406.1 | 30916.76 | 39382.06 | 2317.76 | 11230.7 | 561296.36 | 149.21 | 26082.29 |
| NA20803 | 45634.52 | 3604.9 | 62429.82 | 47230.13 | 1867.46 | 111657.43 | 19951.07 | 31864.98 | 3132.13 | 6157.88 | 611935.18 | 82.74 | 54451.76 |
| NA20804 | 46926.11 | 3983.9 | 34974.42 | 91336.07 | 4319.38 | 165352.68 | 27593.73 | 26084.04 | 2641.95 | 6877.46 | 560555.23 | 83.87 | 29271.16 |
| NA20805 | 54587.16 | 3588.77 | 34025.9 | 61171.07 | 4074.47 | 153939.56 | 30841.88 | 18699.41 | 3642.74 | 6880.73 | 593254.18 | 26.98 | 35267.13 |
| NA20806 | 36129.57 | 5541.05 | 68758.9 | 63550.07 | 1981.49 | 136983.86 | 29318.94 | 40187.47 | 4841.01 | 10631.23 | 558519.22 | 142.38 | 41623.16 |
| NA20807 | 42672.19 | 4673.99 | 63030.47 | 47659.09 | 2229.44 | 141960.34 | 36003.44 | 30292.96 | 3559.28 | 8546.17 | 564399.42 | 117.34 | 54836.31 |
| NA20808 | 33202.88 | 3525.28 | 53981.86 | 83115.92 | 4332.84 | 153326.5 | 74885.08 | 24847.81 | 5668.41 | 15514.35 | 520856.63 | 124.24 | 26509.5 |
| NA20809 | 17535.73 | 2496.08 | 50711.43 | 36514.34 | 2085.33 | 94682.41 | 37483.28 | 26214.07 | 4560.34 | 7667.27 | 693888.3 | 42.13 | 25834.92 |
| NA20810 | 42123.91 | 7110.69 | 53273.62 | 45044.82 | 2198.53 | 120460.81 | 50597.69 | 23649.94 | 3178.45 | 7914.72 | 603893.29 | 383.17 | 40164.07 |
| NA20811 | 22414.9 | 2814.3 | 46478.57 | 33828.44 | 1279.23 | 103404.16 | 31071 | 19188.4 | 2672.16 | 4718.93 | 701584.82 | 71.07 | 30474.02 |
| NA20812 | 19653.93 | 1402.97 | 15826.52 | 17512.55 | 1156.84 | 60955.5 | 25573.5 | 6337.99 | 4455.06 | 5291.92 | 819274.88 | 36.92 | 22496.8 |
| NA20814 | 23974.23 | 4399.8 | 56375.04 | 61393.69 | 2729.74 | 128755.51 | 51712.44 | 32129.54 | 4391.32 | 9342.15 | 595422.18 | 59.34 | 29298.07 |
| NA20815 | 22872.3 | 3067.19 | 73009.68 | 33205.55 | 1587.65 | 108182.46 | 38612.5 | 16790.8 | 3443.24 | 8290.81 | 651483.88 | 49.36 | 39349.33 |
| NA20819 | 25772.54 | 3147.86 | 23630.13 | 24885.88 | 1438.17 | 60725.62 | 37112.46 | 14610.8 | 3389.67 | 4798.15 | 759706.6 | 59.39 | 40722.73 |
| NA20826 | 39508.34 | 4341.04 | 23509.9 | 45605.31 | 3658.18 | 100965.76 | 36191.59 | 29753.19 | 3072.87 | 9413.72 | 668471.37 | 195.1 | 35313.63 |
| NA20828 | 31477.95 | 4054.27 | 47285.22 | 50913.3 | 1639.19 | 105645.92 | 21418.78 | 34302.82 | 3387.66 | 7545.75 | 641421.29 | 163.92 | 50743.92 |
